# Artificial Intelligence Chatbots as Sources of Implant Dentistry Information for the Public: Validity and Reliability Assessment

**DOI:** 10.1055/s-0045-1809155

**Published:** 2025-05-20

**Authors:** Tahani Mohammed Binaljadm, Ahmed Yaseen Alqutaibi, Esam Halboub, Muhammad Sohail Zafar, Samah Saker

**Affiliations:** 1 Department of Substitutive Dental Sciences (Prosthodontics), College of Dentistry, Taibah University, Al Madinah, Saudi Arabia; 2 Department of Prosthodontics, College of Dentistry, Ibb University, Ibb, Yemen; 3 Department of Maxillofacial Surgery and Diagnostic Science, College of Dentistry, Jazan University, Jazan, Saudi Arabia; 4 Department of Clinical Sciences, College of Dentistry, Ajman University, Ajman, United Arab Emirates; 5 Centre of Medical and Bio-allied Health Sciences Research, Ajman University, Ajman, United Arab Emirates; 6 School of Dentistry, Jordan University, Amman, Jordan

**Keywords:** artificial intelligence, dental education, ChatGPT, implantology, prosthodontics

## Abstract

**Objectives:**

This study assessed the reliability and validity of responses from three chatbot systems—OpenAI's GPT-3.5, Gemini, and Copilot—concerning frequently asked questions (FAQs) in implant dentistry posed by patients.

**Materials and Methods:**

Twenty FAQs were prompted to three chatbots in three different times utilizing their respective application programming interfaces. The responses were assessed for validity (low and high threshold) and reliability by two prosthodontic consultants using a five-point Likert scale.

**Statistical Analysis:**

The test of normality was utilized using the Shapiro–Wilk test. Differences between different chatbots regarding the quantitative variables in a given (fixed) time point and between the same chatbots in different time points were assessed using Friedman's two-way analysis of variance by ranks, followed by pairwise comparisons. All statistical analyses were conducted using the SPSS (Statistical Package for Social Sciences) Version 26.0 software program.

**Results:**

GPT-3.5 provided the longest responses, while Gemini was the most concise. All chatbots advised consulting dental professionals more frequently. Validity was high under the low-threshold test but low under the high-threshold test, with Copilot scoring the highest. Reliability was high for all, with Gemini achieving perfect consistency.

**Conclusion:**

Chatbots showed consistent and generally valid responses with some variability in accuracy and details. While the chatbots demonstrated a high degree of reliability, their validity—especially under high-threshold criterion—remains limited. Improvements in accuracy and comprehensiveness are necessary for more effective use in providing information about dental implants.

## Introduction

Artificial intelligence (AI) refers to hardware and software systems designed to replicate human intelligence. It is a tool created to mimic human behavior, enabling machines to simulate cognition, make decisions, and manage complex situations. Over the past two decades, AI has advanced rapidly due to three key components: large datasets generated by digital devices, increased processing power, and improved AI algorithms. These developments have significantly enhanced various aspects of life. 1 AI has permeated all dental specialties, including prosthodontics, dental implants, periodontics, orthodontics, and oral and maxillofacial surgery. 2 3 Most AI applications in dentistry have demonstrated remarkable success, particularly in diagnostic tasks based on optical and radiographic imaging. 4 5

AI-driven chatbots are increasingly utilized to seek information on a wide range of medical and dental topics. For instance, ChatGPT is a prime example of how AI chatbots have rapidly expanded within the healthcare industry. 6 Just 5 days after its launch, ChatGPT reached one million users, and within 2 months, this figure had skyrocketed to 100 million. 7 Studies have shown that ChatGPT's accuracy has improved in various medical education contexts, 8 9 10 but questions remain about its reliability for routine inquiries. 11 12 These AI-powered technologies provide instant access to information about symptoms, treatments, and preventive care, greatly enhancing patient education and engagement. However, the way consumers interact with these AI-powered technologies through well-crafted prompts determines their accuracy in addition to the underlying algorithms and training data. A prompt, which is the specific way a question or instruction is presented to the AI system, significantly influences the quality and accuracy of the response. Biased or poor-quality information, along with inadequately structured prompts, can lead to misleading or unfavorable outcomes. 13

Limited studies evaluated these chatbots in dentistry. One study 14 assessed the accuracy and repeatability of only one chatbot (ChatGPT) in generating answers related to removable dental prostheses and tooth-supported fixed dental prostheses. The results indicated that ChatGPT's reliability in providing correct answers was limited, achieving an accuracy of only 25.6% and a repeatability ranging from substantial to moderate. Another study 15 evaluated the validity and reliability of responses from three AI chatbots—GPT-3.5, Google Bard, and Bing—in the context of the frequently asked questions (FASs) in endodontics. GPT-3.5 demonstrated significantly higher validity compared with the others. All chatbots were found to have acceptable reliability. This study highlighted the potential of AI chatbots to provide credible information on dental topics, particularly endodontics, while also highlighting the need for ongoing assessment of their performance.

In the rapidly evolving field of implant dentistry, AI chatbots have the potential to provide prompt and tailored responses to patient inquiries. However, concerns about the validity and reliability of the information they offer persist, as misinformation could lead to poor decision-making and compromised oral health outcomes. 16 17 18 Despite the growing reliance on these chatbots, there is a significant gap in evaluating the quality of the information they generate, particularly in implant dentistry. Assessing the reliability of AI chatbots is essential to prevent misleading advice. 13 19 20 21 As AI becomes integral to healthcare decision-making, evaluating chatbots' performance will enhance patient education and guide dental professionals in advising patients on the use of these platforms. 20 22 This study aimed at assessing and comparing the reliability and validity of responses provided to frequently asked questions (FAQs) in the field of implant dentistry across three chatbot systems: OpenAI's GPT-3.5, Gemini, and Copilot. The null hypothesis tested was that there were no significant differences in the validity and reliability of the responses provided by these AI systems.

## Materials and Methods

### Data Collection

Twenty FAQs about implant-supported dental prostheses covering a wide range of patient concerns were obtained from two main sources: 10 were selected from a compilation of FAQs encountered during patient consultations with two prosthodontic consultants who specialized exclusively in prosthodontics and implant dentistry, and the remaining 10 questions were derived from the most FAQs regarding implant dentistry as generated by GPT-3.5 upon inquiry. The final list of the 20 FAQs is presented in Table 1 . These FAQs cover a variety of topics such as diagnosis, technical and procedural requirements for treatments, postoperative care, prognosis and treatment outcomes, risks and side effects, preventive actions, and different treatment options.

**Table 1:** The list of 20 frequently asked questions (FAQs)

No.	Question
1.	What is a dental implant?
2.	Is everyone eligible to get a dental implant?
3.	As a diabetic patient, will a dental implant be successful for me?
4.	What impact does smoking have on dental implants?
5.	How soon after a tooth extraction can an implant be placed?
6.	Will the crown be attached right after the implant procedure?
7.	How is the recovery after dental implant placement procedure?
8.	Which is better option to restore a missing tooth FPD or dental implant?
9.	Which is the better option: root canal treatment (RCT) or tooth extraction followed by an implant?
10.	Can the body reject a dental implant?
11.	How long do dental implants typically last?
12.	What are the potential risks associated with dental implants?
13.	Can I get Dental implant during pregnancy?
14.	What is peri-implantitis?
15.	How many dental implants do I need if I want to restore my edentulous upper and lower Jaw?
16.	Do I need to replace my dental implants in the future?
17.	Can I replace my complete denture with implant supported dental prosthesis?
18.	How should I care of my dental implant?
19.	How long does the entire dental implant process take?
20.	Is it possible to get “metal-free” implants?

Each question was submitted to three different AI chatbots at three times (three rounds). The major application programming interface (API) of each chatbot was used to ensure standardized and reproducible interactions. For OpenAI's GPT-3.5, the official OpenAI API ( https://chat.openai.com/ ) was employed. Google's Gemini was accessed through the Google Cloud API ( https://gemini.google.com/ ), while Microsoft's Copilot was interfaced via the Azure OpenAI Service ( https://copilot.microsoft.com ).

A new chat was initiated for each inquiry, and the “More balanced” conversation style was selected for the responses. All questions were submitted to the three chatbots on October 9, 2024, during the initial round of data collection. This procedure was repeated twice, with a 1-week interval between each round. Consequently, each question was posed three times to each chatbot.

### Scoring

The text of each response was assessed by two prosthodontic consultants independently using a 5-point Likert scale. The scoring system was defined as follows: a score of 1 (strongly disagree) was given to the response that was entirely incorrect or irrelevant. A score of 2 (disagree) indicated that the response was mostly incorrect but contained some correct elements. The response that was partially correct but had several inaccuracies, missing elements, or irrelevant details received a score of 3 (neutral). A score of 4 (agree) was assigned to the response that were predominantly correct, though lacking some details or containing minor errors. Finally, the response that was completely accurate and thorough received a score of 5 (strongly agree). When the evaluations were completed, the two reviewers shared their score sheets, each with 180 scores. Any responses that were inconsistent between the evaluators were resolved via discussions supported by evidence, paying particular attention to the responses' context and content. Accordingly, a single consolidated score sheet was prepared for all 180 responses to facilitate the later-on statistical analyses. The methodology for assessing chatbot responses was adapted from Mohammad-Rahimi et al, who evaluated the validity and reliability of AI chatbot responses in endodontics. 15

### Statistical Analysis

#### Analysis of Validity

To evaluate the validity of the chatbot—defined as the extent to which a response answered the intended question—the responses were classified as either “valid” or “invalid.” Two levels of validity, namely a low-threshold test and a high-threshold test, were applied. With regard to the low-threshold test, a score of 4 was established, and a response was considered valid if all three answers to a given question received a score of 4 or higher. Hence, any score that fell below 4 made the response invalid. With regard to the high-threshold test, all three answers must have had a score of 5 to be considered valid. Any score below 5 rendered the response invalid.

#### Analysis of Reliability

Internal consistency, expressed as Cronbach's *α* , was calculated for each of the 20 questions, considering all three responses, to measure the degree of consistency. The values of Cronbach's *α* range from 0 to 1, where a value of 0 signifies a lack of consistency, and a value of 1 denotes a perfect consistency. The higher the *α* value, the more consistent the response of the chatbots, while the lower the *α* value, the less consistent the response of those chatbots. A Cronbach's *α* of ≥0.70 is generally regarded acceptable reliability.

Furthermore, the number of words for each question (response) in each round per each chatbot was obtained. Frequencies and proportions were presented, expressing the qualitative variables. Means and standard deviations, medians and interquartile range, and the range were presented, expressing the quantitative variables. The test of normality was utilized using the Shapiro–Wilk test. Differences between different chatbots regarding the quantitative variables at a given (fixed) time point and between the same chatbots in different time points were assessed using Friedman's two-way analysis of variance by ranks, followed by pairwise comparisons. Differences between different chatbots regarding the qualitative variables (low and high validity, which were taken from the average of the three rounds) were assessed using the McNemar test. A *p* -value of < 0.05 was considered significant.

All statistical analyses were conducted using the SPSS (Statistical Package for Social Sciences) Version 26.0 software program.

## Results

Supplementary Table S1 (available in the online version only) presents the responses of the chatbots to the 20 questions.

The intraclass correlation (ICC) between different chatbots in each round to the 20 questions is presented in Supplementary Table S2 (available in the online version only). Generally, the between-chatbots agreement was weak. Based on ICC results shown in the table, there were weak agreements between different chatbots across the three rounds of evaluation. In round 1, the ICC value was 0.393 (95% CI: 0.117–0.663). This value slightly declined in subsequent rounds, with round 2 showing an ICC of 0.358 (95% CI: 0.083–0.637) and round 3 further decreasing to 0.344 (95% CI: 0.069–0.626).

Table 2 highlights several deficiencies in chatbot responses to FAQs. GPT-3.5 inaccurately linked oral hygiene to implant success in diabetic patients without clarifying the comparable success rates to nondiabetic patients (Question 3). It also failed to detail prosthetic part replacements (Question 11) and suggested antimicrobial mouthwashes without mentioning side effects like dry mouth (Question 18). Copilot lacked comprehensive information about implant materials (Question 1) and did not clarify that there are no absolute contraindications for implants (Question 2). It also provided incorrect information regarding gum recession and overlooked potential damage to adjacent teeth (Question 12). Gemini omitted critical information such as implant materials (Question 1), factors influencing success rates (Question 2), and diabetes-related risks (Question 3). It also included inaccuracies in cost comparisons (Question 9), irrelevant details about procedures like abutment placement (Question 7), and wrong information regarding dental implant placement in pregnant (Question 13).

**Table 2:** List of incorrect, irrelevant, or missing information provided by three chatbots in response to 20 frequently asked questions (FAQs)

Chatbot	Question number	Wrong/irrelevant statement/missing information
GPT-3.5	Q3	**Missing information** It should be clear in the answer that the success of dental implants in well-controlled diabetic patients is similar to nondiabetic patients **Irrelevant information** “Good oral hygiene is critical for reducing the risk of infection and improving healing after the implant procedure”
	Q6	**Missing information** It should be clear at the beginning of the answer that immediate loading is an option that can be done in some cases. In addition, answers should elaborate more about different loading protocols and factors that should be considered. **Irrelevant information:** “Implant placement: A dental implant (a titanium post) is inserted into the jawbone during the surgical procedure. Healing period: After the implant is positioned, it requires a healing period for osseointegration, where the bone integrates with the implant. This phase can last several weeks to months. Abutment placement: Once the implant has healed adequately, an abutment is attached to the implant, which acts as a connector for the crown”
	Q7	**Missing information** Response C. Pain and bleeding are common symptoms after procedures. **Incorrect information** Implant location will affect recovery
	Q8	**Missing information** Explanation of FPD and dental implant in the answer. **Incorrect information** • Fixed partial denture is a less invasive option. • Implant is expensive. *** Examiner comments*:** Implant has a higher initial cost compared with FPD; however, the cost of managing complications from FPD such as loss of vitality of abutment teeth and need for bridge replacement are factors that should be considered when comparing cost of dental implants to FPD
	Q10	**Irrelevant information.** “Systemic Conditions: Conditions such as uncontrolled diabetes, autoimmune diseases, or osteoporosis can affect the body's ability to heal and integrate the implant. Smoking and Poor Oral Hygiene: These factors can increase the risk of complications and may lead to implant failure”
	Q11	**Missing information** Need for prosthetic parts replacement
	Q12	**Incorrect information** “Over time, the bone surrounding the implant may recede” ***Examiner comments*:** After successful osteointegration, dental implants stimulate bone remodelling, so normally bone does not recess with time. “The gums around the implant may recede over time, exposing the metal abutment and compromising aesthetics or causing sensitivity” ***Examiner comments*:** Gingival recession around the implant does not cause sensitivity
	Q14	**Missing information** Response B. missing signs of peri-implantitis
	Q18	**Incorrect information** “Consider using an antimicrobial or antiseptic mouthwash to help reduce bacteria in your mouth, especially after meals” ***Examiner comments*:** Any antimicrobial mouthwashes contain alcohol, which can lead to dry mouth Long-term use of certain mouthwashes cause staining
	Q20	**Incorrect information** “Reduced Sensitivity a : Zirconia implants have lower thermal conductivity than metal implants, which may result in less sensitivity to temperature change” ***Examiner comments*:** Titanium is highly biocompatible and integrates well with bone. This integration creates a stable environment that does not react to temperature fluctuations
Copilot	Q1	**Missing information.** Dental implants can be made from various materials, not only titanium, and did not discuss the complete implant system, abutment, and prosthetic part **Irrelevant information** Benefits of dental implants
	Q2	**Missing information** It should be clear at the beginning of the answer that there are no absolute contraindications to dental implants; however, there are factors that affect success and should be managed before dental implant placement. In addition, there are factors that need to be mentioned that affect dental implant success, such as endodontic infection next to the dental implant or parafunctional habits
	Q11	Missing information, other factors that might affect the survival of dental implants
	Q12	**Incorrect information** Gum Recession: The gum tissue around the implant can recede, exposing the metal post and causing inflammation. ***Examiner comments*:** Gingival recession is typically a consequence of inflammation around a dental implant, rather than the other way around. **Missing information.** Other major risks such as damage to neighboring teeth should be mentioned
	Q13	**Incorrect information** Dental implant requires stronger anaesthetics, which could pose risks to both the mother and the baby ***Examiner comments*:** Anesthesia used for dental implant placement is similar to that used for other dental procedures
	Q14	**Irrelevant information** Peri-implantitis treatment
Gemini	Q1	**Missing information** Dental implants can be made from various materials, not only titanium, and did not discuss the complete implant system, abutment, and prosthetic part
	Q2	**Missing information** It should be clear at the beginning of the answer that there are no absolute contraindications to dental implants, however, there are some factors that affect success and should be managed before dental implant placement. In addition, there are factors that need to be mentioned that affect dental implant success such as endodontic infection next to the dental implant or parafunctional habits
	Q3	**Missing information** It should be clear in the answer that the success of dental implants in well-controlled diabetic patients is similar to nondiabetic patients Other information important to patients such as how uncontrolled diabetes may affect healing or its effect on implant infection **Irrelevant information** “Practicing excellent oral hygiene, such as brushing twice daily, flossing, and using a mouthwash, can help prevent complications and promote successful healing”
	Q5	**Missing information** No specific timeline provided
	Q6	**Missing information** “Dental implant immediate loading is an option that should be considered. Different loading protocols and considerations for each should be mentioned”
	Q7	**Irrelevant information** “Abutment placement: A connector piece is placed on the implant Crown placement: The final restoration, which looks like a natural tooth, is attached to the abutment”
	Q8	**Missing information** Explanation of FPD and dental implant in the answer **Incorrect information** Fixed partial denture is a less invasive option ***Examiner comments*:** FPD considered invasive procedures
	Q9	**Incorrect information** “RCT is less expensive than the extraction and subsequent implant procedure” “Implants can offer a permanent solution and often last longer than RCT-treated teeth” ***Examiner comments*** **:** Cost of RCT in molars teeth, adding the cost of the crown and possible need for Re-RCT should be considered. Success of RCT is high
	Q10	**Missing information** Dental titanium allergy, one of the main causes of rejection, is not mentioned
	Q11	**“Bite alignment:** Proper bite alignment helps distribute forces evenly on the implants” ***Examiner comments*:** Not clear what does the statement mean
	Q13	**Incorrect information** Long-term effects of dental implants on the developing fetus
	Q14	**Irrelevant information** Professional cleaning: A dentist or periodontist can clean the implant and surrounding tissues to remove bacteria and plaque Antibiotics: Antibiotics may be prescribed to treat infections Surgical treatment: In severe cases, surgery may be necessary to clean the implant or regenerate bone
	Q15	**Missing information** Numbers in each jaw Fixed or removable option

Note: It is important to note that this table presents examples illustrating why each chatbot did not achieve a score.

a Reduced Sensitivity refers to a decrease in the ability to perceive sensory stimuli.

Table 3 presents the word count across different chatbots in the different rounds. The lowest word count was obtained by Gemini, with an average word count of 174 ± 46, ranging from 101 to 271. Similarly, the range of word count for Gemini was the narrowest, ranging from 101 to 300 words. In contrast, the average word count for GPT-3.5 was the highest (230 ± 66.7), ranged from 135 to 361, and showed the widest range of variation (142–434 words in Round 1). The average count of words for Copilot was intermediate compared with the others (187 ± 57), ranging from 104 to 309. Between chatbots, there were statistical differences in word count with *p* = 0.001, 0.004, 0.002, and 0.001 for the first, second, third round, and the average of the three rounds, respectively. Within the chatbot, there were no statistical differences in word count between the three round for GPT 3.5 ( *p* = 0.392), Copilot ( *p* = 0.588), and Gemini ( *p* = 0.890).

**Table 3:** Mean, standard deviations, range, median, and interquartile ranges of the word numbers obtained by the different chatbots at different time points

Round	Chatbots									*p* -Value a
	GPT 3.5			Copilot/Bing			Google Bard/Gemini			
	Mean ± SD	Range	Median (IQR)	Mean ± SD	Range	Median (IQR)	Mean ± SD	Range	Median (IQR)	
1	237.9 ± 78.21 ^A^	142–434	212.5 (193.5- 168.5)	189.2 ± 62.78	101–337	188 (143.25–220.75)	175.0 ± 51.31	101–300	163.5 (139–210.75)	0.001
2	226.5 ± 60.11 ^A^	128–348	212.5 (185.25–274.75)	185.85 ± 54.95	104–303	187.5 (142.75–213)	170/1 ± 41.63	102–271	167.5 (138.25–197)	0.004
3	227.9 ± 73.04 ^A^	100–352	211 (177.5–301.25)	185.95 ± 55.09	104–303	186 (142.75–217.75)	177.65 ± 51.13	102–300	167.5 (138.25–210.75)	0.002
Average	230.62 ± 66.71 ^A^	135.33–361.67	212 (190.25–282.92)	187.33 ± 57.12	104–309.33	186 (143.17–217.42)	174.27 ± 46.1	101.67–271	167.67 (139.42–211)	0.001
p-Value b	0.393			0.588			0.890			

a Friedman's test between different chatbots in a fixed time point (read by rows). Capital letter superscript indicates significant difference within different chatbots based on post hoc pairwise comparisons.

b Friedman's test between the same chatbots in the different time points (read by columns); the average values were not included.

**Fig. 1:**
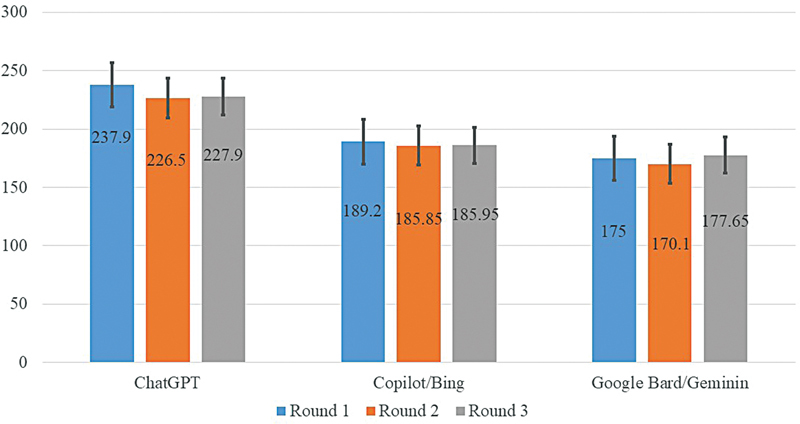
Average word numbers obtained by the different chatbots at different time points.

Fig. 1 provides a visual depiction of the word count for each chatbot in each round.

All chatbots recommended consulting a dentist for additional information: Gemini advised 42 out of 60 times, GPT-3.5 29 times, and Copilot 28 times.

**Fig. 2:**
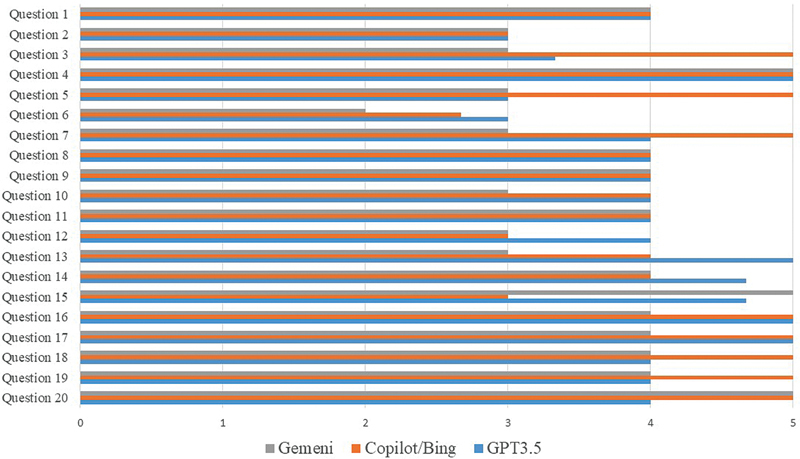
Mean scores for the responses of the chatbot to 20 frequently asked questions.

The scores for the question in the three rounds are illustrated in Fig. 2 . These scores ranged from 2 to 5. Notably, Gemini was the sole chatbot that received a score of 2 on one question (Q6); all other scores fell within the range of 3 to 5.

**Fig. 3:**
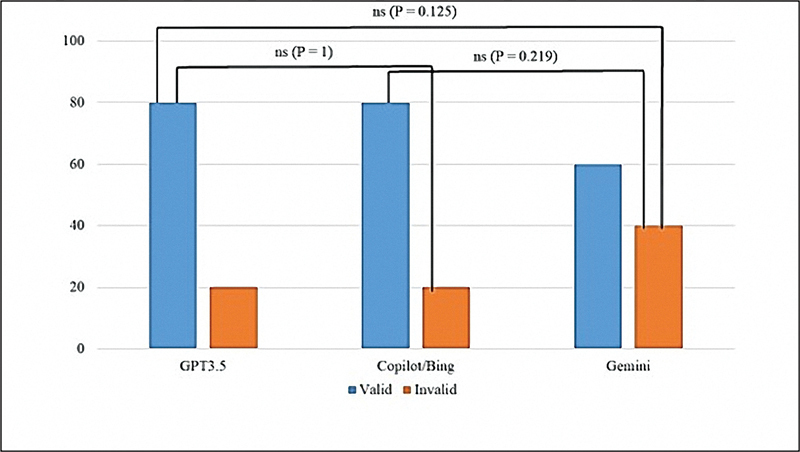
A low-threshold validity test (the significant level was at *p* > 0.05).

As shown in Fig. 3 regarding the low-threshold validity, all chatbots exhibited relatively high validity levels. GPT-3.5 and Copilot demonstrated validity rates of 80%; 16 out of 20 of their responses classified as valid. Conversely, Gemini reported a validity rate of 60% (12 out of 20). There were no statistical differences between these chatbots.

**Fig. 4:**
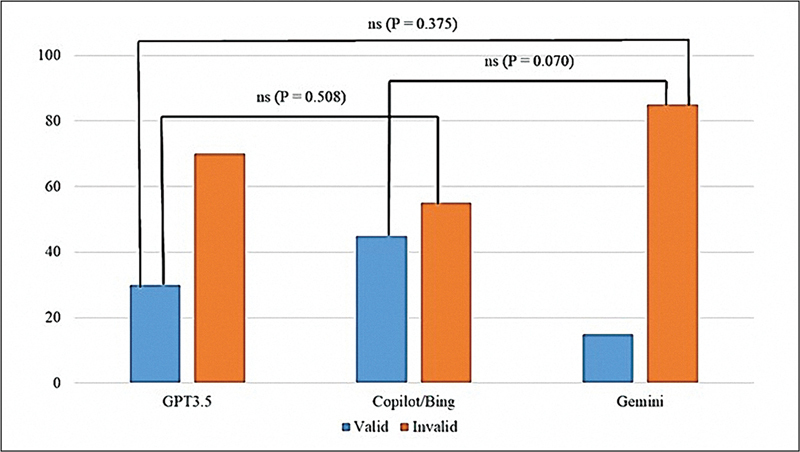
A high-threshold validity test (the significant level was at *p* > 0.05).

When evaluated using a high-threshold test ( Fig. 4 ), the validity of responses produced by all three chatbots was found to be relatively low. Copilot achieved the highest validity rate, with 45% (9 out of 20) of its responses classified as valid. In contrast, both GPT-3.5 and Gemini demonstrated lower validity rates, with only 20% (4 out of 20) and 15% (3 out of 20) of their responses, respectively, being deemed valid.

Gemini achieved perfect reliability with an *α* of 1.00, suggesting perfect consistency. Copilot also showed high reliability with an *α* of 0.975, and GPT-3.5 demonstrated high reliability with an *α* of 0.859. These results suggest that all chatbots consistently provided similar responses across repeated queries ( Table 4 ).

**Table 4:** The scores of responses obtained by the different Chatbots

Chatbot	Median (IQR)	Mean ± SD	Range	Low-threshold validity (valid responses, *N* (%))	High-threshold validity (valid responses, *N* (%))	ICC (95% CI)
ChatGPT	4 (4–4.7) ^a^	4.1 ± 0.66	3–5	16 (80)	4 (30)	0.859 (0.731–0.936)
Copilot/Bing	4 (4–5) ^a^	4.23 ± 0.82	2.67–5	16 (80)	9 (45)	0.975 (0.949–0.989)
Google Bard/Gemini	4 (3–4) ^b^	3.7 ± 0.8	2–5	12 (60)	3 (15)	1 (1–1)

Notes: Related samples Friedman's two-way analysis of variance by ranks, followed by pairwise comparison. Similar superscript small letters mean insignificant difference, while different superscript small letters indicate significant difference.

## Discussion

This study aimed to assess and compare the reliability and validity of responses provided by three AI chatbots—OpenAI's GPT-3.5, Gemini, and Copilot—when addressing FAQs in the field of dental implants. The study selected OpenAI's GPT-3.5, Gemini, and Copilot chatbots due to their advanced capabilities in natural language processing, widespread use, and accessibility through standardized APIs. These platforms are commonly utilized in both academic and practical healthcare settings, making them suitable for assessing AI responses in implant dentistry. Other chatbots were excluded due to limited public accessibility or lower recognition in the field of AI-driven medical applications.

The study's results revealed some inconsistencies between different chatbots, especially regarding the size (words count) of the texts obtained. The study also revealed some variabilities at the level of validity and reliability between these chatbots—a matter which entails working hard to rectify such variabilities and inconsistencies. In fact, the presence of such AI-obtained information may introduce a crisis of trust between the patients and the dental practitioners.

While AI chatbots have the potential to transform how laypeople acquire medical information, including information in dentistry, they also introduce risks of misinformation. Therefore, it is crucial for medical and dental associations to ensure that the information disseminated by these systems is accurate and to educate the public on both the benefits and limitations of chatbots use in healthcare settings. 19 20 Although complete regulation of chatbots may not be feasible, it remains the responsibility of researchers to verify the information these tools provide. With the increasing use of AI chatbots in healthcare, they present opportunities to enhance patient satisfaction and reduce costs, particularly in dentistry, where they can be valuable for patients' seeking information about dental implants. 23

This study marks the first evaluation of the validity and reliability of AI chatbots in addressing queries related to dental implants. It highlights the importance of carefully considering both the phrasing of the questions and the sources that inform chatbot responses, as these factors can significantly influence the quality of the information provided. In this investigation, ten questions were formulated by practicing prosthodontists, alongside ten FAQs generated by GPT-3.5, which has been trained on a vast array of datasets, including academic literature. 6 However, the challenge of designing representative and inclusive questions persists, especially in ensuring the queries adequately reflect the complexities of dental health.

AI chatbots, while demonstrating high validity at lower thresholds of assessment, tend to falter when evaluated at more stringent thresholds. This decline in performance is particularly concerning in the context of dental health, where misinformation can lead to severe consequences. 21 An example from this study involved Gemini recommending stronger anesthetics for dental implant placement in pregnant, a statement that lacked clarity and could pose risks to both patients and medical practitioners. 24 25 Such errors underscore the need for ongoing, rigorous scrutiny of chatbot-generated information to prevent the spread of potentially harmful advice.

A previous study comparing the validity and reliability of GPT-3.5, Google's Bard (now evolved into Gemini), and Bing (now evolved into Copilot) in addressing endodontic FAQs have yielded findings consistent with our study. 15 At lower validity thresholds, there were no significant differences among these chatbots. At higher thresholds, however, GPT-3.5 demonstrated superior validity. 15 In our study, although no significant differences were observed among the platforms at the higher thresholds, all chatbots displayed acceptable reliability. These variations in performance can be attributed to differences in the underlying technologies and training datasets of each systems. 26 For instance, GPT-3.5 is designed to generate human-like text based on extensive training data, 27 while Gemini and Copilot, utilize bidirectional encoder representations from transformers (natural language processing) and operate with less transparency regarding their technical foundations. 16 17 18

The reliability of chatbot responses, defined as the consistency of the information provided, is another critical factor. While Gemini exhibited a consistent output with time, GPT-3.5 and Copilot produced a wider range of responses. This variability could offer users a broader perspective but also raises concerns regarding reliability, as the inherent randomness of deep learning models may contribute to inconsistent outputs. 18

Despite the validity of chatbot responses in some areas, significant errors emerged, particularly when addressing more complex queries. For example, GPT-3.5 inaccurately suggested that oral hygiene significantly affects implant success in diabetic patients, without acknowledging that success rates are comparable to nondiabetic individuals. Other responses were either lacking in detail or incorrect, such as the recommendation of antimicrobial mouthwashes without discussing potential side effects. Gemini also faltered by omitting crucial details about implant materials, success factors, and diabetes-related risks, while Copilot's responses were similarly flawed, misrepresenting facts and providing incomplete information on important topics like gingival recession. These errors highlight the need for continuous refinement of AI chatbot technologies, particularly for complex health-related questions.

A limitation of current chatbots, including GPT-3.5, is their inability to provide references or citations for the claims they make. This lack of verifiable sources poses a significant risk, as the information provided may be unreliable or outdated. 6 Improving chatbot's ability to cite reputable sources would greatly enhance their credibility, especially in healthcare contexts. Future research should focus on incorporating insights from both clinicians and patients from diverse socioeconomic and cultural backgrounds to create more inclusive and accurate AI models.

Patients should consult a dentist for personalized advice. AI chatbots may provide inaccurate or incomplete information, particularly on complex dental issues like implants or treatments for specific conditions such as diabetes. Chatbots can be useful for basic queries, but their responses should be verified through credible sources or healthcare professionals. Dentists are encouraged to educate patients on chatbot limitations, correct misinformation, and collaborate on improving AI systems for more reliable healthcare applications. Chatbot managers should focus on enhancing accuracy, consistency, and user interfaces to ensure medical advice is properly referenced and backed by reliable sources.

The ethical use of AI in dentistry demands thoughtful evaluation of when and how to apply it, ensuring it supports patient care without undermining professional judgment, accountability, or privacy. 28 AI should be implemented only when it demonstrably enhances health outcomes and is cost-effective, underpinned by legal regulations, informed consent, and clinical oversight. 29 While AI can advance diagnostics, improve access, and reduce costs, it presents ethical concerns such as bias from non-representative data, lack of transparency, automation bias, and unequal outcomes. Dentists must be properly educated and trained to use AI responsibly, remain accountable for its application, and ensure that AI tools are rigorously tested for safety and fairness. Transparent data management, patient communication, and disclosure of any potential conflicts of interest are essential to maintain trust and ensure AI is integrated ethically and effectively into dental practice. 30

This study has the following limitations that should be taken into account when interpreting its findings. A significant limitation was the small number of evaluators—only two prosthodontists with dental implant expertise assessed the validity of chatbot responses. A larger panel of experts would have provided a more comprehensive evaluation of the reliability and accuracy of the chatbots studied. Another limitation was including only 20 FAQs which may not fully represent the complete spectrum of patient inquiries regarding dental implants. Additionally, the single-point temporal evaluation does not consider the ongoing updates and improvements in AI systems, which may render the results quickly outdated due to the rapid advancement of AI technology.

This study offers a comprehensive comparison of three leading AI chatbots using clinically relevant FAQs in implant dentistry, evaluated by prosthodontic experts. The structured, reproducible methodology enhances the reliability and applicability of the findings. The clinical significance is that AI chatbots have the potential to support patient education by delivering accessible information on implant procedures. Understanding their strengths and limitations can help clinicians guide patients in using these tools safely and effectively. The results highlight the need for continuous evaluation and refinement of AI chatbots in dental contexts. Future studies could explore prompt optimization and chatbot training to improve accuracy and clinical relevance.

## Conclusion

The texts generated over time by various chatbots exhibit instability, demonstrating variations in word count, validity, and reliability. In terms of strict validity, the information produced by these chatbots is suboptimal, potentially jeopardizing users' health if it is trusted and acted upon. Although the reliability of the data obtained is high, the suitability and usability of this information are questionable. While chatbot responses may be consistent, they may not address individual patient needs or clinical complexities. Therefore, patients should consult their dentist for accurate and personalized information.
