# Supplementary material for: Artificial Intelligence Chatbots as Sources of Implant Dentistry Information for the Public: Validity and Reliability Assessment
Source: Eur J Dent. 2025 May 20;20(2):466–76. doi: 10.1055/s-0045-1809155 (PMC13160598; doi:10.1055/s-0045-1809155)
Supplement: Supplementary file 1 — Supplementary Material [file 10-1055-s-0045-1809155-s2524086.pdf]

Supplementary Table S1 Three rounds of responses generated by evaluated chatbots to each question

Chat GPT 3.5

Q1: What is a dental implant?

A. A dental implant is a surgical component used to replace missing teeth. It consists of three main components:

1. Implant post: A screw-like structure, usually made of titanium, that is surgically inserted into the jawbone. It serves as the root of the missing tooth.
2. Abutment: A connector that attaches to the implant post, linking it to the replacement tooth or prosthesis
3. Prosthesis: The visible replacement tooth (crown, bridge, or denture) that is attached to the abutment and designed to look and function like a natural tooth.

Dental implants provide a strong, permanent foundation because they fuse with the bone through a process called **osseointegration**, making them function much like natural teeth.

B. A dental implant is a medical device used to replace missing teeth. It consists of three main parts:

1. Implant post: A small, screw-like structure, usually made of titanium, that is surgically inserted into the jawbone to act as the root of the missing tooth
2. Abutment: A connector that is placed on the implant post to hold the artificial tooth or teeth securely in place.
3. Prosthesis (crown, bridge, or denture): The visible, artificial tooth that is attached to the abutment, designed to blend in with natural teeth.

Dental implants provide a long-lasting and stable solution for tooth loss because they integrate with the bone (a process called osseointegration), making them function much like natural teeth. They are widely used in both single-tooth replacement and full-mouth reconstructions.

C. A dental implant is a medical device used to replace missing teeth. It consists of a titanium post (or screw) that is surgically inserted into the jawbone to serve as a stable foundation for a replacement tooth, bridge, or denture. Over time, the implant fuses with the bone in a process called osseointegration, providing a secure and durable base. The implant mimics the function of a natural tooth root and helps maintain the structure of the jawbone and surrounding teeth.
Dental implants are a popular option in restorative dentistry because they offer a long-lasting and natural-looking solution for tooth loss.

Q2: Is everyone eligible to get a dental implant?

A. Not everyone is eligible for dental implants, as several factors influence a person's suitability for the procedure. The main considerations include:

1. Bone health: Implants require sufficient bone density in the jaw to support the implant. Patients with significant bone loss may need bone grafts before being considered.
2. Oral health: Good gum health is crucial, as implants rely on healthy surrounding tissues. Patients with gum disease (periodontitis) may need treatment before getting implants.
3. Systemic health: Certain medical conditions, such as uncontrolled diabetes, immune disorders, and other chronic diseases, can affect healing and the long-term success of implants.
4. Age: Implants are typically placed in adults, as young individuals with developing bones may not be ideal candidates. However, there's no strict upper age limit.
5. Smoking: Smoking can impair healing and increase the risk of implant failure. Patients are often advised to quit smoking before and after the procedure.
6. Jaw size or shape: Some patients may have anatomical limitations, such as small or irregularly shaped jaws, that make implants difficult without prior interventions like grafts.
7. Medication: Certain medications, especially those affecting bone metabolism (like bisphosphonates), can influence implant success.

Oral examination and imaging studies are usually performed by dental professionals to assess eligibility. In cases where patients aren't immediately suitable, additional treatments, such as bone augmentation, may be recommended.

B. Not everyone is eligible to get a dental implant. Several factors determine whether a person is a good candidate for this procedure:

1. Bone quality and quantity: Adequate jawbone density is needed to anchor the implant. If the bone has deteriorated or is insufficient, a bone graft might be required before implant placement.
2. Gum health: Healthy gums are essential for supporting implants. People with untreated gum disease or periodontal issues may need treatment before implants can be considered.
3. Overall health: Chronic health conditions like uncontrolled diabetes, immune system disorders, or conditions affecting healing (e.g., osteoporosis) can complicate implant success. These conditions need to be well-managed.
4. Age: Implants are not typically recommended for younger individuals whose bones are still growing. For adults, there's no upper age limit, but overall health is a greater factor than age itself.
5. Smoking: Smoking can impair healing and increase the risk of implant failure. Dentists may recommend quitting or reducing smoking before considering an implant.
6. Medications: Some medications, especially those affecting bone metabolism (like bisphosphonates), can affect the success of implants.
7. Oral hygiene commitment: Patients need to be committed to maintaining excellent oral hygiene after implant placement to ensure long-term success.

C. Not everyone is eligible for a dental implant. Several factors determine a patient's eligibility, including:

1. Bone health: Dental implants need a strong and healthy jawbone for stability. If the bone is insufficient or weak, bone grafting might be necessary before implant placement.
2. Gum health: Healthy gums are essential. Patients with gum disease (periodontitis) may need treatment before an implant procedure.

 © 2025. The Author(s).

Supplementary Table S1 (*Continued*)

3. Systemic health: Certain medical conditions like uncontrolled diabetes, autoimmune diseases, or conditions affecting bone healing can impact eligibility. Such conditions can interfere with recovery or the integration of the implant into the bone.
4. Smoking: Smoking can affect healing and implant success. Dentists may recommend quitting smoking for better outcomes.
5. Age: Implants are generally not recommended for younger patients whose jaws are still developing. In older adults, there is no upper age limit as long as the person is in good health.
6. Medication: Some medications, like bisphosphonates used to treat osteoporosis, can impact bone healing and may pose a risk for implant procedures.

In cases where immediate placement isn't feasible, additional treatments (e.g., bone grafting) might be recommended to improve candidacy for dental implants. A thorough evaluation by a dental professional is necessary to determine if a patient is suitable for this treatment.

Q3: As a diabetic patient, will a dental implant be successful for me?

A. Yes, dental implants can be successful for diabetic patients, but there are some important considerations. Diabetes, especially if poorly controlled, can increase the risk of complications after dental implant surgery, such as delayed healing, infection, or even implant failure. However, if your blood sugar levels are well-managed, the risk of complications can be significantly reduced.

Some factors to keep in mind as a diabetic patient considering a dental implant:

1. Blood sugar control: Well-controlled diabetes improves healing and reduces the risk of infection. It's crucial to work closely with your doctor to maintain stable blood glucose levels before and after the surgery.
2. Bone health: Diabetes can affect bone density, which is important for implant success. Your dentist may need to assess your bone quality and consider bone grafting if necessary.
3. Oral hygiene: Good oral hygiene is critical for reducing the risk of infection and improving healing after the implant procedure.
4. Regular monitoring: Ongoing check-ups with your dentist and healthcare provider will help monitor the healing process and ensure the implant integrates properly with your jawbone.

With proper management, diabetic patients can have a successful dental implant outcome. It's essential to consult both your dentist and your doctor to ensure all health factors are addressed before proceeding with the implant.

B. Yes, as a diabetic patient, dental implants can be successful, but it's important to take special precautions to improve the outcome. The success of dental implants in diabetic patients depends largely on how well your diabetes is controlled. Here are some key factors to consider:

1. Blood sugar control: Keeping your blood glucose levels within a healthy range is essential. Poorly controlled diabetes can impair wound healing and increase the risk of infection, which may affect the success of the implant.
2. Healing process: Diabetes can slow the healing process, so your dentist or oral surgeon may need to monitor your recovery more closely after the procedure to ensure that the implant is integrating properly with your bone.
3. Bone quality: Implants require strong, healthy bone for stability. Diabetes can sometimes affect bone density and quality, which is why your dentist may perform a thorough evaluation to check the condition of your jawbone.
4. Infection risk: Diabetic patients are at a higher risk of developing infections, so maintaining excellent oral hygiene before and after the surgery is crucial. Antibiotics may be prescribed as a preventive measure.
5. Presurgery assessment: Your dentist will likely collaborate with your physician to assess your overall health and ensure that your diabetes is well-managed before proceeding with the implant.

Many diabetic patients with controlled blood sugar levels experience successful dental implants. Discuss your specific case with both your dentist and healthcare provider to make sure you're prepared for the procedure.

C. As a diabetic patient, a dental implant can still be successful, but certain precautions are important to ensure a favorable outcome. Diabetes can affect your body's ability to heal, especially if your blood sugar levels are not well controlled. Here are some factors to consider:

1. Blood sugar control: Well-managed diabetes significantly increases the chances of a successful dental implant. High blood sugar levels can impair healing and increase the risk of infection, which can lead to implant failure.
2. Bone health: Diabetes can sometimes reduce bone density and slow the healing of bone around the implant (osseointegration). If your jawbone is too weak, additional treatments like bone grafting may be needed before placing the implant.
3. Infection risk: Diabetics are more prone to infections, including in the gums and surrounding tissues. Good oral hygiene and regular dental check-ups are crucial to prevent infections like peri-implantitis (inflammation around the implant).
4. Healing time: Healing might take longer if you have diabetes, so your dentist will monitor your progress closely after the procedure.
5. Pre- and postsurgical care: Your dentist might recommend antibiotics or other treatments to reduce the risk of infection, and will likely emphasize the importance of maintaining good blood sugar control during the healing process.

By working closely with both your dentist and your physician, and ensuring that your diabetes is well controlled, the likelihood of a successful dental implant increases.

Q4. What impact does smoking have on dental implants?

A. Smoking has a significant negative impact on the success of dental implants. Here are some of the key effects:

1. Impaired healing**: Smoking reduces blood flow to the tissues, which affects the healing process after implant surgery. This can delay osseointegration, where the implant fuses with the jawbone, leading to potential failure.
2. Increased risk of peri-implantitis: Smokers have a higher risk of developing peri-implantitis, an inflammatory condition affecting the soft and hard tissues surrounding dental implants. This can lead to bone loss and implant failure.

(*Continued*)

 © 2025. The Author(s).

Supplementary Table S1 (*Continued*)

3. Decreased implant success rate: Studies have shown that the long-term success rate of implants is lower in smokers compared to non-smokers. Smoking increases the likelihood of complications such as infection and implant rejection.
4. Bone resorption: Smoking accelerates bone loss, which can reduce the stability of the implant over time. In severe cases, bone resorption may require additional procedures like bone grafting.
5. Higher failure rates: Several studies indicate that implant failure rates are significantly higher in smokers, especially in heavy smokers. The failure rate can be double that of non-smokers, depending on the duration and intensity of smoking.
6. Decreased osseointegration: Nicotine and other toxins in cigarettes affect the ability of the bone to bond with the implant. Poor osseointegration increases the likelihood of early implant failure.

For patients who smoke, it is often recommended to quit smoking before undergoing implant surgery to improve the chances of success.

B. Smoking can significantly impact the success and longevity of dental implants. Here are the main effects:

1. Impaired healing: Smoking restricts blood flow and oxygen supply to the tissues, which is crucial for healing. This can delay the osseointegration process, where the implant fuses with the jawbone.
2. Increased infection risk: Smokers are at a higher risk of postoperative infections, such as peri-implantitis, due to reduced immune response and compromised oral health.
3. Bone quality and density: Smoking can lead to decreased bone quality and density, which are essential for providing stable support for dental implants. Poor bone health can hinder the successful integration of the implant.
4. Higher failure rates: Studies have shown that dental implants in smokers have a significantly higher failure rate compared to non-smokers. This can be attributed to impaired healing and increased risk of complications.
5. Soft tissue complications: Smoking adversely affects gum health, increasing the likelihood of periodontal disease, which can compromise the supporting structures around implants.
6. Aesthetic concerns: Smoking can cause staining of teeth and surrounding tissues, affecting the aesthetic appearance of dental implants. Given these risks, dental professionals often advise patients to quit smoking before undergoing implant surgery to improve the chances of a successful outcome.

C. Smoking has a detrimental effect on dental implants, impacting both short-term healing and long-term success. Here's a detailed breakdown of the negative impacts smoking has on dental implants:

1. Impaired healing
   - Reduced blood flow: Smoking constricts blood vessels and reduces blood flow to the oral tissues, which impairs healing after implant surgery. This affects both the soft tissues and bone integration around the implant.
   - Delayed osseointegration: Osseointegration, the process where the implant bonds with the jawbone, is slower in smokers. Nicotine and other chemicals in tobacco interfere with bone formation.
2. Higher risk of infection
   - Smoking increases the likelihood of infections post-surgery. Nicotine suppresses the immune response, making it harder for the body to fight off bacteria that may cause infections around the implant site.
3. Increased risk of peri-implantitis
   - Inflammation Around Implants: Smokers are more prone to peri-implantitis, an inflammatory condition affecting the tissues around the implant. This condition can lead to bone loss and implant failure.
   - Chronic inflammation: The toxins in cigarettes can cause chronic inflammation, which puts stress on the implant and surrounding tissue, increasing the chances of failure.
4. Higher implant failure rates
   - Studies consistently show that smoking doubles or even triples the failure rate of dental implants compared to non-smokers. Smokers face an increased risk of both early failure (within the first few months) and late failure (after years of function).
   - Failure of bone grafting: For patients who require bone grafting prior to implant placement, smoking significantly reduces the success of grafting procedures due to impaired healing.
5. Bone resorption
   - Accelerated bone loss: Smoking accelerates bone resorption (loss) around the implant, reducing long-term stability. This is especially problematic in the areas where bone density is already low, such as the upper jaw.
   - Bone density reduction: The nicotine in cigarettes reduces the density of bone, making it more challenging for the implant to properly integrate and remain stable over time.
6. Decreased long-term success
   - Prosthetic complications: Even if the initial implant placement is successful, smokers face a higher rate of complications related to the prosthetic parts of the implant, such as the crown or bridge placed on the implant.
   - **Aesthetic Complications**: Smoking can lead to aesthetic problems around implants, including gum recession and staining of the surrounding tissues.

7. **Recommendations for Smokers**
   - **Quit Smoking**: Dental professionals typically advise patients to quit smoking before implant surgery and for several months after the procedure to improve the chances of success.
   - **Reduced Smoking**: In cases where quitting is not an option, reducing smoking can also positively impact healing and implant longevity.

In summary, smoking has a multi-faceted negative impact on the success of dental implants, from delaying healing to increasing the risk of implant failure. Quitting or reducing smoking is one of the best ways to ensure successful implant integration and long-term results.

Supplementary Table S1 (*Continued*)

Q5: How soon after a tooth extraction can an implant be placed?

A. The timing for placing a dental implant after a tooth extraction can vary based on several factors, including the condition of the bone, the health of the patient, and the presence of infection or other complications. Here are some general guidelines:

1. **Immediate Implant Placement**: In some cases, an implant can be placed immediately after the tooth extraction. This is often done if there is enough healthy bone to support the implant and if the extraction site is infection-free.
2. **Early Implant Placement**: If immediate placement isn't feasible, the implant may be placed a few weeks to a few months after extraction, allowing time for any healing and potential bone regeneration.
3. **Delayed Implant Placement**: In cases where significant bone loss has occurred or if the extraction site needs time to heal, implant placement may be delayed for several months or even longer. This period allows for the bone to heal and potentially regenerate, sometimes requiring bone grafting.

Typically, a dental professional will evaluate the extraction site through clinical examination and imaging (like X-rays or CT scans) to determine the best timing for implant placement. If you're considering an implant after an extraction, it is essential to discuss your specific situation with your dentist or oral surgeon.

B. The timing for placing a dental implant after a tooth extraction can vary depending on several factors, including the condition of the bone, the type of extraction performed, and the overall health of the patient. Here are some general guidelines:

1. **Immediate Implant Placement**: An implant can sometimes be placed immediately after tooth extraction if:
   - There is sufficient healthy bone to support the implant.
   - The extraction site is free of infection.
   - The tooth was removed without trauma to the surrounding bone.
2. **Early Implant Placement**: If immediate placement isn't possible, the implant may be placed anywhere from a few weeks to a few months after the extraction. This allows time for initial healing.
3. **Delayed Implant Placement**: In cases of significant bone loss or when infection is present, implant placement may be delayed for several months. This period allows for the extraction site to heal and may involve bone grafting to restore the bone before placing the implant.

Generally, it is crucial to consult with a dental professional who can assess your specific situation and recommend the best approach. They will consider factors like the health of your gums and bone, any underlying health conditions, and the complexity of the extraction.

C. The timing for placing a dental implant after a tooth extraction can vary based on several factors, including the condition of the bone and the overall health of the patient. Here are some common scenarios:

1. **Immediate Implant Placement**: An implant can often be placed immediately after the tooth is extracted if:
   - There is sufficient bone density and quality.
   - The extraction site is free of infection.
   - The tooth is removed atraumatically (without damaging surrounding tissues).
2. **Early Implant Placement**: If immediate placement isn't possible, the implant may be placed a few weeks to a few months after extraction, allowing time for the initial healing of the extraction site.
3. **Delayed Implant Placement**: In cases where there is significant bone loss or infection, the placement may be delayed for several months. This allows for proper healing and, if necessary, bone grafting procedures to rebuild the bone before the implant is placed.

Ultimately, the best course of action should be determined by a dental professional who can evaluate your specific situation through clinical examination and imaging.

Q6: Will the crown be attached right after the implant procedure?

A. Generally, a crown is not attached immediately after the implant procedure. Here's how the process typically works:

1. **Implant Placement**: The dental implant (titanium post) is placed in the jawbone during surgery.
2. **Healing Period**: After the implant is placed, a healing period is required for osseointegration, where the bone fuses with the implant. This can take several weeks to months.
3. **Abutment Placement**: Once the implant has healed, an abutment is placed on top of the implant. This connects the implant to the crown.
4. **Crown Placement**: After the gum tissue has healed around the abutment, a custom crown is attached.

In some cases, an immediate crown may be placed right after the implant surgery, but this depends on factors like the implant's stability and the specific treatment plan. Your dentist will evaluate your situation to determine the best approach.

B. Typically, a crown is not attached right away after an implant procedure. Here's how the process generally unfolds:

1. **Implant Placement**: A dental implant (titanium post) is surgically inserted into the jawbone.
2. **Healing Period**: Following the implant placement, a healing phase is necessary for osseointegration, during which the bone fuses with the implant. This process can take several weeks to months.
3. **Abutment Placement**: Once the implant has sufficiently healed, an abutment is attached to the top of the implant, serving as a connector between the implant and the crown.
4. **Crown Placement**: After the gum tissue has healed around the abutment, a custom crown is affixed.

In some instances, an immediate crown may be placed right after the implant surgery, but this is contingent on factors such as the stability of the implant and the specific treatment plan. Your dentist will assess your individual situation to determine the most suitable approach.

C. In most cases, a crown is not placed immediately following an implant procedure. The typical process is as follows:

1. **Implant Placement**: A dental implant (a titanium post) is inserted into the jawbone during the surgical procedure.
2. **Healing Period**: After the implant is positioned, it requires a healing period for osseointegration, where the bone integrates with the implant. This phase can last several weeks to months.

(*Continued*)

Supplementary Table S1 (*Continued*)

3. **Abutment Placement**: Once the implant has healed adequately, an abutment is attached to the implant, which acts as a connector for the crown.
4. **Crown Placement**: After the gum tissue has healed around the abutment, a custom crown is placed on top.

There are situations where an immediate crown may be placed at the time of the implant surgery, depending on factors like the implant's stability and the overall treatment plan. Your dentist will evaluate your specific case to determine the best course of action.

Q7: How is the recovery after dental implant implacment procedure?

A. Recovery after a dental implant placement can vary depending on individual health factors and the complexity of the procedure. However, the typical recovery process includes several stages:
Immediate Post-Procedure (First 24 Hours)
- **Swelling and Discomfort**: Some swelling, bruising, and discomfort around the implant site are common. Pain can usually be managed with prescribed or over-the-counter pain medications.
- **Bleeding**: Light bleeding is normal. Patients are advised to bite down on gauze for about 30-45 minutes to control bleeding.
First Few Days
- **Diet**: Soft foods are recommended to avoid irritating the implant site. Avoid hot drinks and alcohol.
- **Oral Hygiene**: Care must be taken not to disturb the implant site, but keeping the area clean is essential. Gentle rinsing with salt water may be suggested after 24 hours.
- **Activity Level**: It's advisable to rest and avoid strenuous activities for a few days.
First Week
- **Follow-Up Appointment**: A follow-up visit may be scheduled to monitor healing and remove any sutures, if necessary.
- **Continued Care**: Patients should continue to manage pain and swelling with medications as directed.
Weeks 2 to 6
- **Healing Process**: Osseointegration, where the bone fuses with the implant, occurs over several weeks. Patients may experience minimal discomfort during this period.
- **Gradual Return to Normal Diet**: As healing progresses, patients can gradually return to a normal diet, but should still avoid very hard or chewy foods.
Long-Term Recovery
- **Complete Healing**: Full integration of the implant into the jawbone can take several months. Follow-up appointments will be scheduled to assess the healing process.
- **Final Restoration**: Once healing is complete, the final crown or prosthetic tooth can be placed on the implant.
Important Considerations
- **Signs of Complications**: Patients should be aware of signs of infection (increased swelling, redness, or discharge) and contact their dentist if they occur.
- **Individual Variability**: Recovery times can vary based on factors such as the number of implants placed, the patient's overall health, and adherence to post-operative instructions.

Following the dentist's post-operative care instructions closely is crucial for a smooth recovery process.

B. Recovery after a dental implant placement involves several stages and varies from person to person. Here's a comprehensive overview:
Immediate Recovery (First 24 Hours)
- **Post-Operative Care**: Expect some swelling and discomfort around the implant site. Pain can be managed with prescribed medications or over-the-counter pain relievers.
- **Bleeding**: Light bleeding is normal. Patients may be instructed to bite down on gauze for 30-45 minutes to help control it.
First Few Days
- **Diet**: Stick to soft foods (e.g., yogurt, mashed potatoes) to avoid irritating the implant area. Avoid hot foods and drinks.
- **Oral Hygiene**: Maintain oral hygiene but be gentle around the implant site. Your dentist may recommend rinsing with salt water after 24 hours.
- **Activity**: Rest is important; avoid strenuous activities or heavy lifting for a few days.
First Week
- **Follow-Up Appointment**: A follow-up visit may be scheduled to assess healing and possibly remove sutures.
- **Pain Management**: Continue to manage discomfort with pain relievers as needed.
Weeks 2 to 6
- **Healing Process**: Osseointegration, where the bone fuses with the implant, occurs during this period. Discomfort should gradually decrease.
- **Dietary Gradual Return**: You can gradually reintroduce more solid foods, but still avoid very hard or sticky items
Long-Term Recovery
- **Complete Integration**: Full osseointegration can take several months. Follow-up appointments will be necessary to monitor progress.
- **Final Restoration**: Once healed, a crown or prosthetic tooth will be placed on the implant.
Important Considerations
- **Signs of Complications**: Watch for signs of infection (increased swelling, persistent pain, or discharge) and contact your dentist if these occur.
- **Individual Variability**: Recovery times can vary based on factors such as the number of implants, overall health, and adherence to post-operative care instructions.

 © 2025. The Author(s).

Supplementary Table S1 (*Continued*)

Following your dentist's guidelines closely is crucial for a smooth recovery and successful implant integration.
C. Recovery after a dental implant placement procedure typically involves several stages, and while individual experiences can vary, here's a general overview of what to expect:
Immediately After the Procedure
- **Anesthesia:** Local anesthesia is used during the procedure, so you may feel numbness in the treatment area.
- **Rest:** It's advised to rest for the remainder of the day. You may experience some bleeding, which should gradually decrease.
First Few Days
- **Swelling and Discomfort:** Swelling of the gums and face may occur, peaking around 48 hours after the procedure. Discomfort can be managed with prescribed or over-the-counter pain medications.
- **Diet:** Stick to soft foods and avoid hot liquids to minimize discomfort. Foods like yogurt, applesauce, and mashed potatoes are good choices.
Week One
- **Hygiene:** Oral hygiene is crucial. Gently rinse with warm salt water (after 24 hours) to help keep the area clean. Avoid brushing directly on the implant site for the first few days.
- **Follow-Up:** A follow-up appointment may be scheduled to monitor healing.
Weeks Two to Six
- **Healing:** Most swelling and discomfort should subside within a week or so. The implant site may still feel tender.
- **Diet Gradually Returns:** You can start reintroducing more solid foods as you feel comfortable.
Long-Term (Months)
- **Osseointegration:** The implant integrates with the jawbone over the next few months. This process is critical for the stability of the implant. Regular check-ups will be needed during this period.
- **Final Restoration:** After sufficient healing, the final prosthetic crown is placed on the implant.
Factors Affecting Recovery
- **Overall Health:** Conditions like diabetes or smoking can impact healing time.
- **Implant Location:** Recovery can vary depending on whether the implant is placed in the upper or lower jaw.
- **Bone Quality:** Adequate bone density and health are crucial for successful osseointegration.
Signs of Complications
Be aware of the following signs that may indicate complications:
- Excessive bleeding
- Severe pain not managed by medication
- Signs of infection (e.g., fever, pus)
- Implant mobility or unusual swelling

Q8: Which is better option to restore a missing tooth FPD or dental implant?

A. The choice between a fixed partial denture (FPD) and a dental implant for restoring a missing tooth depends on various factors, including the patient's specific situation, preferences, and overall dental health. Here are some considerations for each option:
Dental Implant
Advantages:
1. **Preservation of Bone:** Implants stimulate the jawbone, preventing bone loss that typically occurs with missing teeth.
2. **Longevity:** Implants can last many years, often a lifetime, with proper care.
3. **Aesthetics:** They look and feel like natural teeth and do not affect adjacent teeth.
4. **Functionality:** Implants provide excellent chewing function and do not shift like dentures.

Disadvantages:
1. **Surgical Procedure:** Requires surgery, which may be a concern for some patients.
2. **Healing Time:** There is a healing period required for osseointegration (the process where the bone fuses with the implant).
3. **Cost:** Generally more expensive than FPDs.
Fixed Partial Denture (FPD)
Advantages:
1. **Non-Surgical:** No surgical procedure is required, making it a less invasive option.
2. **Immediate Results:** Typically quicker to place than an implant since it can often be completed in a single appointment.
3. **Cost:** Generally less expensive than dental implants.

Disadvantages:
1. **Impact on Adjacent Teeth:** Requires alteration of adjacent healthy teeth to support the bridge.
2. **Bone Loss:** Does not prevent bone loss in the jaw, as it does not stimulate the bone.
3. **Longevity:** FPDs may need replacement over time, often lasting 5-15 years.
Conclusion
- **Dental Implants** are often preferred for long-term tooth replacement due to their durability and benefits in bone preservation.
- **FPDs** may be a suitable option for those who prefer a non-surgical solution or have specific circumstances that make implants less feasible.

Ultimately, the best choice should be made in consultation with a dental professional, who can assess individual needs and conditions.

(*Continued*)

 © 2025. The Author(s).

Supplementary Table S1 (*Continued*)

B. The choice between a fixed partial denture (FPD) and a dental implant for restoring a missing tooth depends on various factors, including the patient's overall dental health, budget, and personal preferences. Here's a more detailed comparison to help you determine which option might be better:
Fixed Partial Denture (FPD)
Advantages:
- **Less Invasive:** Requires minimal surgical intervention, often just tooth preparation of adjacent teeth.
- **Cost-Effective:** Generally less expensive than dental implants, especially with insurance coverage.
- **Faster Treatment:** The process can usually be completed in fewer appointments compared to implants.

Disadvantages:
- **Requires Tooth Preparation:** Involves reshaping adjacent healthy teeth, which may compromise their integrity.
- **Potential for Issues with Adjacent Teeth:** If the supporting teeth develop problems, the FPD may need to be replaced.
- **Does Not Prevent Bone Loss:** FPDs do not address the underlying bone loss that occurs after tooth loss.

Dental Implant
Advantages:
- **Preserves Bone Health:** Stimulates the jawbone, preventing resorption and maintaining bone density.
- **Longevity:** Implants generally last longer than FPDs with proper care, often many years or even a lifetime.
- **No Impact on Adjacent Teeth:** Does not require alteration of neighboring teeth, preserving their health and structure.

Disadvantages:
- **Invasive Procedure:** Requires surgical placement of the implant, which may involve a more complex procedure.
- **Higher Initial Cost:** Typically more expensive than FPDs, with costs that can vary based on the case.
- **Longer Treatment Timeline:** The process may take several months, including healing and integration time.

Conclusion
If preserving adjacent teeth and minimizing invasiveness is a priority, an FPD might be more suitable. However, if long-term durability and maintaining bone health are important, a dental implant may be the better choice. It's best to consult with a dental professional who can assess your specific situation and recommend the most appropriate option based on your individual needs and preferences.
C. The choice between a fixed partial denture (FPD) and a dental implant for restoring a missing tooth depends on various factors, including patient-specific conditions and preferences. Here's a comparison to help you decide:
Dental Implant
Advantages:
1. **Bone Preservation:** Dental implants stimulate the jawbone, preventing bone resorption that typically occurs after tooth loss.
2. **Longevity:** Implants can last a lifetime with proper care and maintenance.
3. **Aesthetic Appeal:** They look and function like natural teeth and do not affect adjacent teeth.
4. **Stability and Functionality:** Implants provide excellent chewing function and are not prone to movement like dentures.

Disadvantages:
1. **Surgical Procedure:** Requires a surgical procedure to place the implant, which may be a concern for some patients.
2. **Healing Time:** Implants require time for osseointegration (the process where the bone fuses to the implant).
3. **Cost:** Typically more expensive than FPDs.

Fixed Partial Denture (FPD)
Advantages:
1. **Non-Invasive:** No surgical procedure is required, making it a less invasive option.
2. **Immediate Results:** Can often be placed in a single appointment, providing quick restoration.
3. **Cost-Effective:** Generally less expensive than dental implants.

Disadvantages:
1. **Impact on Adjacent Teeth:** Requires alteration of adjacent healthy teeth for support, potentially compromising their integrity.
2. **Bone Loss:** Does not prevent bone loss in the jaw over time, as it does not stimulate the bone.
3. **Limited Longevity:** FPDs may need to be replaced or repaired over time, often lasting 5–15 years.

Conclusion
- **Dental Implants** are usually the better option for long-term restoration, especially for preserving bone and achieving optimal aesthetics and functionality.
- **FPDs** may be a suitable alternative for patients who prefer a non-surgical approach or have certain conditions that make implants less feasible.

Consulting with a dental professional is essential to evaluate individual circumstances and make the best choice for tooth restoration.
9. **Which is the better option: root canal treatment (RCT) or tooth extraction followed by an implant?**
A. The choice between root canal treatment (RCT) and tooth extraction followed by an implant depends on several factors, including the condition of the tooth, overall dental health, and patient preferences. Here are some considerations for both options:
Root Canal Treatment (RCT)
Pros:
- **Tooth Preservation:** RCT allows you to keep your natural tooth, which is beneficial for maintaining bone density and alignment of adjacent teeth.
- **Less Invasive:** The procedure is typically less invasive than extraction and implant placement.

 © 2025. The Author(s).

Supplementary Table S1 (*Continued*)

- **Cost-Effective:** RCT is generally less expensive than the combined costs of extraction and implant placement.
- **Immediate Use:** After RCT, the tooth can often be restored with a crown relatively quickly.
Cons:
- **Success Rates:** While RCT is generally successful, there is a chance of reinfection or treatment failure, which may require further procedures.
- **Time-Consuming:** Multiple visits may be necessary, depending on the severity of the infection.
Tooth Extraction and Implant
Pros:
- **Long-Term Solution:** Implants have a high success rate and can last many years with proper care.
- **Improved Functionality:** Implants can function similarly to natural teeth and help maintain facial structure.
- **No Root Canal Risks:** Eliminates any potential issues related to the existing tooth, such as reinfection.
Cons:
- **Invasiveness:** The extraction and subsequent implant placement are more invasive procedures.
- **Bone Loss:** Extraction can lead to bone loss over time, but implants can help mitigate this if placed promptly.
- **Higher Cost:** Generally more expensive due to the cost of the implant and any associated procedures.
- **Healing Time:** There is a longer recovery time before the implant is fully functional compared to RCT.
Factors to Consider
1. **Condition of the Tooth:** If the tooth is severely damaged or infected, extraction may be necessary. If there is enough healthy tooth structure, RCT may be preferred.
2. **Patient's Overall Health:** Certain medical conditions may influence the decision, as well as the patient's ability to undergo surgery.
3. **Personal Preference:** Some patients may prefer to retain their natural teeth, while others may prefer the idea of an implant.
Conclusion
Ultimately, the decision should be made in consultation with a dentist or endodontist who can evaluate your specific situation. They can provide insights based on the condition of the tooth, your dental health, and your long-term goals.
B. Choosing between root canal treatment (RCT) and tooth extraction followed by an implant involves weighing several important factors. Here's a detailed comparison to help you make an informed decision:
Root Canal Treatment (RCT)
Pros:
1. **Preservation of Natural Tooth:** RCT aims to save your natural tooth, which is beneficial for maintaining dental structure and function.
2. **Less Invasive:** RCT is typically less invasive than extraction and implant surgery, usually requiring only one or two visits.
3. **Cost-Effective:** Generally, RCT is less expensive than the extraction and subsequent implant procedure.
Cons:
1. **Potential for Reinfection:** There is a possibility of reinfection, which may require additional treatment or even extraction later.
2. **Multiple Appointments:** RCT can sometimes require several visits to complete.
3. **Weakened Tooth:** After RCT, the tooth may become more brittle and might require a crown for protection.
Tooth Extraction Followed by an Implant
Pros:
1. **Long-Term Solution:** Implants can offer a permanent solution and often last longer than RCT-treated teeth.
2. **Bone Health:** Implants stimulate the jawbone, helping to maintain bone density and prevent bone loss.
3. **Functionality:** Implants closely mimic natural teeth in terms of function and aesthetics.
Cons:
1. **Invasive Procedure:** The extraction and implant placement are more invasive and involve surgery.
2. **Longer Treatment Timeline:** The entire process can take several months, from extraction to healing and implant placement.
3. **Higher Cost:** This option is generally more expensive due to the costs associated with surgery and the implant itself.
Considerations
- **Condition of the Tooth:** If the tooth is severely damaged or has a poor prognosis, extraction might be the best option.
- **Patient's Overall Health:** Some medical conditions can affect the success of implants.
- **Personal Preference:** Some patients prefer to keep their natural teeth if possible.
Conclusion
Ultimately, the best option depends on the specific circumstances surrounding your dental health. It's crucial to consult with a dental professional who can evaluate your situation, consider your health history, and discuss your preferences. They can provide personalized recommendations to help you make the right choice for your oral health.
C. The choice between root canal treatment (RCT) and tooth extraction followed by an implant depends on several factors, including the condition of the tooth, your overall dental health, and personal preferences. Here's a breakdown of both options:
Root Canal Treatment (RCT)
Pros:
- **Tooth Preservation:** RCT allows you to keep your natural tooth, which is important for maintaining bone density and the alignment of surrounding teeth.
- **Less Invasive:** RCT is generally less invasive than extraction and implant surgery.
- **Cost-Effective:** RCT is typically less expensive than extraction followed by an implant.

(*Continued*)

 © 2025. The Author(s).

Supplementary Table S1 (*Continued*)

- **Immediate Restoration:** After treatment, the tooth can often be restored with a crown, allowing for quicker function.
Cons:
- **Success Rate:** While RCT is often successful, there is a chance of reinfection or the need for retreatment.
- **Multiple Visits:** RCT may require several appointments, depending on the severity of the infection.
Tooth Extraction and Implant
Pros:
- **Long-Term Solution:** Implants have a high success rate and can last many years with proper care.
- **Functional and Aesthetic:** Implants function like natural teeth and can help maintain facial structure.
- **No Root Issues:** Eliminates any potential complications associated with the existing tooth.
Cons:
- **Invasive Procedure:** Extraction and implant placement are more invasive and involve surgery.
- **Higher Cost:** Generally more expensive than RCT due to the costs of the implant and associated procedures.
- **Healing Time:** There may be a longer recovery period before the implant is fully functional compared to RCT.
Considerations
- **Condition of the Tooth:** If the tooth is severely damaged or infected beyond repair, extraction may be necessary. If there's enough healthy tooth structure, RCT may be preferable.
- **Patient's Overall Health:** Medical conditions or risk factors may influence the decision.
- **Personal Preference:** Some patients prioritize retaining their natural teeth, while others may prefer the permanence of an implant.
Conclusion
Ultimately, the decision should be made in consultation with a dentist or specialist who can assess your specific situation. They can provide tailored recommendations based on the condition of your tooth, your overall dental health, and your long-term goals.

10. **Can the body reject a dental implant?**

A. Yes, the body can reject a dental implant, although this is not as common as rejection in other types of implants (like organ transplants). The rejection of a dental implant is usually related to several factors, including:
1. **Allergic Reactions**: Some patients may have allergic reactions to the materials used in the implant, such as titanium or other alloys.
2. **Infection**: If bacteria enter the surgical site, it can lead to infection, which may compromise the implant's stability and integration with the bone.
3. **Poor Osseointegration**: For a dental implant to be successful, it must integrate properly with the jawbone. Factors like insufficient bone density, improper placement, or underlying health issues can prevent this.
4. **Autoimmune Disorders**: Patients with autoimmune diseases may have a higher risk of complications, including rejection of implants.
5. **Poor Oral Hygiene**: Neglecting oral hygiene can lead to peri-implantitis, an inflammatory condition that can lead to implant failure.
6. **Smoking**: Tobacco use can hinder healing and osseointegration, increasing the risk of implant failure.

While complete rejection is rare, complications can arise that may require the implant to be removed. It's essential for patients to follow post-operative care instructions and attend regular follow-up appointments to monitor the implant's health.

B. Yes, the body can reject a dental implant, though it's relatively rare. Rejection usually doesn't occur in the same way it does with organ transplants, but there can be complications related to the body's response to the implant. Here are some reasons why this might happen:
1. **Allergic Reactions**: Some patients may have allergies to the materials used in the implant, typically titanium or zirconia. This can lead to inflammation and failure of the implant.
2. **Infection**: Peri-implantitis, an infection around the implant site, can cause bone loss and may lead to implant failure if not properly treated.
3. **Poor Bone Integration**: For a dental implant to be successful, it needs to integrate with the jawbone through a process called osseointegration. If the bone is insufficient or the implant is placed improperly, it may not integrate well.
4. **Systemic Conditions**: Conditions such as uncontrolled diabetes, autoimmune diseases, or osteoporosis can affect the body's ability to heal and integrate the implant.
5. **Smoking and Poor Oral Hygiene**: These factors can increase the risk of complications and may lead to implant failure.

If a dental implant fails, it's important to consult with a dental professional to determine the cause and explore potential solutions.

C. Yes, the body can reject a dental implant, although outright rejection is rare. Here are some reasons why complications may occur:
1. **Allergic Reactions**: Some individuals may have allergies to the materials used in dental implants, such as titanium, leading to localized inflammation or discomfort.
2. **Infection**: If bacteria enter the surgical site, it can cause infection (peri-implantitis), jeopardizing the stability and integration of the implant with the bone.
3. **Osseointegration Failure**: Successful dental implants require proper integration with the jawbone. Factors such as insufficient bone density, improper placement, or medical conditions can hinder this process.
4. **Autoimmune Disorders**: Patients with autoimmune conditions may be at a higher risk for complications, potentially leading to implant failure.
5. **Poor Oral Hygiene**: Neglecting oral care can result in infections and inflammation, which can compromise the implant.
6. **Smoking**: Tobacco use can negatively affect healing and osseointegration, increasing the likelihood of implant complications.

Supplementary Table S1 (*Continued*)

While rejection is uncommon, complications can lead to the need for implant removal or replacement. Regular follow-ups and good oral hygiene are crucial for the success of dental implants.

11. How long do dental implants typically last?

A. Dental implants typically last between 10 to 15 years, but with proper care, they can last 20 years or more. Factors that influence their longevity include:

1. **Oral Hygiene:** Maintaining good oral hygiene is crucial for the health of the implants and surrounding tissues.
2. **Bone Health:** Sufficient bone density and quality are necessary for successful integration of the implant.
3. **Material:** Most dental implants are made of titanium, which is strong and resistant to corrosion.
4. **Lifestyle Choices:** Factors like smoking, diet, and alcohol consumption can affect healing and longevity.
5. **Health Conditions:** Certain medical conditions, such as diabetes or autoimmune disorders, can impact implant success.
6. **Dental Care:** Regular check-ups with a dentist can help monitor the health of the implants and surrounding gums.

With diligent care, many people enjoy the benefits of their dental implants for many years

B. Dental implants can last a long time, often ranging from **10 to 30 years** or even longer with proper care. Factors influencing their longevity include:

1. **Oral Hygiene:** Good dental care, including regular brushing and flossing, helps prevent infections.
2. **Bone Quality:** Adequate jawbone density is essential for the stability of the implant.
3. **Lifestyle Choices:** Smoking, excessive alcohol consumption, and poor nutrition can negatively affect healing and longevity.
4. **Professional Skill:** The expertise of the dental surgeon and the quality of the materials used play significant roles.
5. **Regular Maintenance:** Routine dental check-ups can help monitor and maintain implant health.

On average, studies indicate that dental implants have a success rate of about **95%** after **10 years**. Many people enjoy their implants for **20 years or more** with the right care.

C. Dental implants can last a long time, often ranging from **10 to 30 years** or even longer with proper care. Factors influencing their longevity include:

1. **Oral Hygiene:** Good dental care, including regular brushing and flossing, helps prevent infections.
2. **Bone Quality:** Adequate jawbone density is essential for the stability of the implant.
3. **Lifestyle Choices:** Smoking, excessive alcohol consumption, and poor nutrition can negatively affect healing and longevity.
4. **Professional Skill:** The expertise of the dental surgeon and the quality of the materials used play significant roles.
5. **Regular Maintenance:** Routine dental check-ups can help monitor and maintain implant health.

On average, studies indicate that dental implants have a success rate of about **95%** after **10 years**. Many people enjoy their implants for **20 years or more** with the right care.

12. **What are the potential risks associated with dental implants?**

A. Dental implants are a popular and generally safe solution for replacing missing teeth, but like any surgical procedure, they come with potential risks and complications. Here are some of the most common risks associated with dental implants:

1. **Infection at the Implant Site**
- **Peri-implantitis:** A form of gum disease that occurs around the implant, potentially leading to bone loss and implant failure if untreated. It is often caused by poor oral hygiene or bacterial infection.

2. **Nerve Damage**
- If the implant is placed too close to a nerve, it can cause numbness, tingling, or pain in the gums, lips, chin, or tongue. This is more common in lower jaw implants.

3. **Sinus Problems**
- Implants placed in the upper jaw can sometimes protrude into the sinus cavities, leading to sinus issues. This is more likely when there is insufficient bone height in the upper jaw.

4. **Implant Failure**
- Though rare, implants may not properly integrate with the jawbone (osseointegration), causing them to become loose and fail. Factors like smoking, poor oral hygiene, or insufficient bone quality can increase the risk.

5. **Bone Loss**
- Over time, the bone surrounding the implant may recede. This can lead to implant instability and failure, especially if peri-implantitis is present.

6. **Allergic Reactions**
- Some patients may have allergic reactions to the titanium or other materials used in implants. This can lead to inflammation and discomfort, though this is rare.

7. **Inadequate Bone for Implant Placement**
- If the patient doesn't have enough bone to support the implant, it can lead to complications. Bone grafts or sinus lifts are sometimes necessary to address this.

8. **Overloading of the Implant**
- If the implant is subjected to excessive forces too soon (due to biting or chewing), it may not heal properly, leading to failure. Following the dentist's post-surgical instructions is crucial.

9. **Rejection of the Implant**
- In rare cases, the body may reject the implant, which can be due to an immune response or poor biocompatibility of the implant material.

10. **Gum Recession**

(*Continued*)

 © 2025. The Author(s).

Supplementary Table S1 (*Continued*)

- The gums around the implant may recede over time, exposing the metal abutment and compromising aesthetics or causing sensitivity.

11. **Bleeding and Swelling**

- After surgery, some degree of bleeding and swelling is expected, but excessive bleeding or infection can occur in rare cases.

While these risks exist, they can usually be minimized with proper patient selection, surgical technique, and aftercare. Patients with good overall health, proper bone density, and excellent oral hygiene are less likely to experience complications.

B. Dental implants are generally considered safe and effective, but like any surgical procedure, they come with potential risks and complications. Here are some of the key risks associated with dental implants:

1. **Infection**: There is a risk of infection at the implant site, which can lead to complications and failure of the implant.
2. **Nerve Damage**: Implants can sometimes damage nearby nerves, leading to pain, tingling, or numbness in the gums, lips, or chin.
3. **Sinus Problems**: For implants placed in the upper jaw, there may be a risk of sinus perforation, leading to sinus infections or other issues.
4. **Implant Failure**: The implant may fail to integrate with the bone or may become loose over time, leading to the need for removal.
5. **Bone Loss**: Insufficient bone density or quality can lead to complications, including the risk of bone loss around the implant.
6. **Gum Recession**: The gums may recede around the implant, exposing the metal post and leading to aesthetic concerns or increased sensitivity.
7. **Allergic Reactions**: Some individuals may have allergic reactions to the materials used in the implant (e.g., titanium).
8. **Complications from Other Health Conditions**: Patients with certain health conditions, such as uncontrolled diabetes or autoimmune disorders, may face higher risks and complications.
9. **Peri-implantitis**: This is an inflammatory condition that affects the tissues around the implant, similar to gum disease, and can lead to bone loss.
10. **Cost and Maintenance**: Dental implants can be expensive, and ongoing maintenance and care are necessary to ensure their longevity.

It's important for individuals considering dental implants to discuss these risks with their dental professional and undergo a thorough evaluation to determine their suitability for the procedure.

C. Dental implants are generally considered safe and effective for replacing missing teeth, but they do come with potential risks and complications. Here are some of the key risks associated with dental implants:

1. **Infection**: There is a risk of infection at the implant site, which can hinder healing and may require antibiotic treatment or, in severe cases, removal of the implant.
2. **Nerve Damage**: Implants can sometimes damage nearby nerves, leading to pain, tingling, or numbness in the gums, lips, or chin.
3. **Sinus Problems**: For implants placed in the upper jaw, there may be a risk of sinus perforation, which can lead to sinus infections or other sinus-related issues.
4. **Implant Failure**: The implant may fail to integrate properly with the bone, resulting in loosening or loss of the implant. Factors such as poor bone density, infection, or excessive pressure can contribute to this risk.
5. **Bone Loss**: If there isn't enough bone to support the implant, it may lead to complications, including the risk of bone loss around the implant site.
6. **Gum Recession**: The gums may recede around the implant, which can expose the metal post and lead to aesthetic concerns or increased sensitivity.
7. **Allergic Reactions**: Some individuals may experience allergic reactions to the materials used in the implant (e.g., titanium).
8. **Complications from Other Health Conditions**: Certain health conditions, such as uncontrolled diabetes, autoimmune disorders, or smoking, can increase the risks of complications and implant failure.
9. **Peri-implantitis**: This is an inflammatory condition affecting the tissues around the implant, similar to gum disease, which can result in bone loss and implant failure if not managed properly.
10. **Cost and Maintenance**: Dental implants can be expensive, and they require ongoing maintenance and care, including regular dental visits and proper oral hygiene.

Before proceeding with dental implants, it's essential for individuals to discuss these risks with their dental professional, who can assess their individual situation and help determine the best course of action.

13. Can I get dental implant during pregnancy?

A. Getting a dental implant during pregnancy is generally not recommended unless it's an emergency situation. Here are a few reasons why dentists advise postponing elective procedures like dental implants until after childbirth:

1. **Risk of Infection and Medications**: Dental implant surgery involves a surgical procedure that may require antibiotics or pain medications, some of which may not be safe for pregnant women.
2. **Radiographs (X-rays)**: Dental implants require imaging, like X-rays, to assess the bone and surrounding structures. Although dental X-rays can be done with proper shielding, most dentists prefer to avoid any unnecessary exposure to radiation during pregnancy.
3. **Body Changes**: Hormonal changes during pregnancy can affect oral health, making gums more sensitive and prone to conditions like gingivitis. This can impact healing and increase the risk of complications.
4. **Stress and Discomfort**: Undergoing surgery can add stress, which is not ideal during pregnancy, especially in the first and third trimesters when it's best to avoid surgical procedures.

 © 2025. The Author(s).

Supplementary Table S1 (*Continued*)

If the implant is not urgent, most dentists will recommend waiting until after pregnancy. However, if you're experiencing significant pain or infection that requires immediate attention, it's essential to discuss the best options with your dentist and obstetrician.
B. Getting dental implants during pregnancy is generally not recommended, especially in the first and third trimesters. Here are some key points to consider:
1. **Timing**: The second trimester is usually considered the safest period for dental procedures, including implants. However, it's best to consult with your healthcare provider and dentist to determine the best timing based on your specific situation.
2. **Risks**: During pregnancy, there are increased risks associated with anesthesia and infection, both of which can affect the mother and the fetus.
3. **Healing**: Pregnancy can alter the immune system and hormone levels, potentially affecting the healing process after an implant surgery.
4. **Dental Health**: It's important to maintain good dental health during pregnancy, as hormonal changes can lead to issues like pregnancy gingivitis. Regular dental check-ups and cleanings are crucial.
5. **Consultation**: Always discuss your dental needs and any planned procedures with both your dentist and your obstetrician to ensure the safety of both you and your baby.

If you're considering dental implants while pregnant, it's essential to get professional advice tailored to your health and circumstances.
C. It is generally not recommended to get dental implants during pregnancy due to several important factors:
1. **Timing**: The safest time for dental procedures, including implants, is typically during the second trimester (weeks 14 to 27). However, it's still advisable to consult with your healthcare provider and dentist before proceeding.
2. **Anesthesia and Medication**: Dental implant surgery involves anesthesia, which can carry risks during pregnancy. Some medications used during the procedure may not be safe for the developing fetus.
3. **Surgical Risks**: Pregnancy can affect the immune system, potentially increasing the risk of infection or complications following surgery.
4. **Radiation Exposure**: Dental implants often require X-rays for planning, and while the exposure is minimal, it is best to avoid unnecessary radiation during pregnancy.
5. **Healing Process**: Hormonal changes during pregnancy can affect the healing process, possibly complicating recovery after surgery.
6. **Consultation**: It is crucial to discuss any dental plans with both your dentist and obstetrician. They can provide personalized advice based on your health and pregnancy status.

If you need dental work during pregnancy, it may be safer to focus on preventive care and address more invasive procedures after childbirth.

Q14: What is peri-implantitis?

A. Peri-implantitis is an inflammatory condition affecting the tissues around dental implants. It is characterized by the presence of plaque and calculus accumulation, leading to inflammation of the peri-implant mucosa and, in more advanced stages, loss of supporting bone around the implant.
Key Features of Peri-implantitis:
1. **Symptoms**: Common symptoms include redness, swelling, and bleeding of the gums around the implant, as well as potential pain or discomfort. Patients may also notice pus or a bad taste in the mouth.
2. **Risk Factors**:
   - Poor oral hygiene
   - Smoking
   - History of periodontal disease
   - Systemic conditions such as diabetes
   - Excessive loading or mechanical factors on the implant
3. **Diagnosis**: Diagnosis is typically made through clinical examination, radiographic assessment, and probing of the implant site to assess bone loss and the condition of the peri-implant tissues.
4. **Treatment**: Treatment may involve mechanical debridement to remove plaque and calculus, local or systemic antibiotics, and surgical intervention in severe cases to restore health to the peri-implant tissues.
5. **Prevention**: Maintaining good oral hygiene, regular dental check-ups, and lifestyle modifications (like quitting smoking) are crucial for preventing peri-implantitis.

Early detection and management are essential to prevent further complications, such as implant failure.
B. Peri-implantitis is an inflammatory condition affecting the tissues surrounding dental implants. It is characterized by the following features:
Definition
Peri-implantitis is defined as a pathological condition involving inflammation of the peri-implant mucosa and progressive loss of the supporting bone around a dental implant.
Key Features
1. **Inflammation**: The condition results in inflammation of the gums (peri-implant mucosa) around the implant, which can lead to further complications if untreated.
2. **Bone Loss**: A hallmark of peri-implantitis is the loss of alveolar bone that supports the implant, which can jeopardize the stability and longevity of the implant.

Risk Factors
Several factors may contribute to the development of peri-implantitis, including:

(*Continued*)

Supplementary Table S1 (*Continued*)

- Poor oral hygiene
- A history of periodontal disease
- Smoking
- Systemic diseases (e.g., diabetes, autoimmune conditions)
- Mechanical overload or excessive stress on the implant
Diagnosis
Diagnosis involves:
- **Clinical Examination**: Assessing gum health and probing depth.
- **Radiographic Evaluation**: Using X-rays to evaluate bone levels around the implant.
Treatment
Management strategies include:
- **Mechanical Debridement**: Cleaning the implant surface to remove plaque and calculus.
- **Antibiotic Therapy**: Administering local or systemic antibiotics to manage infection.
- **Surgical Intervention**: In severe cases, surgery may be necessary to restore bone and soft tissue health.
Prevention
Preventive measures are crucial and may involve:
- Maintaining good oral hygiene practices
- Regular dental check-ups
- Addressing risk factors such as smoking and systemic health issues

Early detection and management of peri-implantitis are essential to ensure the success of dental implants and prevent further complications.

C. Peri-implantitis is an inflammatory condition affecting the soft and hard tissues surrounding dental implants. It is characterized by inflammation in the gum tissues (peri-implant mucosa) and progressive bone loss around the implant, which can lead to the implant's failure if left untreated.

The condition typically begins as **peri-implant mucositis**, a reversible inflammation limited to the soft tissues without affecting the bone. However, if not managed properly, it can progress to peri-implantitis, where bone loss occurs. Common signs include:
- Redness and swelling of the gums around the implant
- Bleeding when probing the area
- Pocket formation around the implant
- Potential suppuration (pus)
- Pain or discomfort, though this is often minimal
- Progressive bone loss visible on radiographs

Peri-implantitis can result from several factors, including poor oral hygiene, excessive mechanical stress on the implant, smoking, systemic health issues (such as diabetes), and certain genetic predispositions. The condition is complex and often requires professional intervention for treatment, which may include non-surgical and surgical approaches depending on the severity.

Would you like more information on specific treatments or risk factors?

Q15: How many dental implants do I need if I want to restore my edentulous upper and lower Jaw?

A. The number of dental implants needed to restore an edentulous upper and lower jaw depends on several factors, including:
1. **Jawbone Density**: The quality and quantity of bone available for implantation can influence the number of implants required. Bone grafting might be necessary if there is significant bone loss.
2. **Type of Restoration**:
   - **Full Arch Restoration**: For a complete restoration of both the upper and lower jaw, you might need 4 to 6 implants per arch using techniques like All-on-4 or All-on-6, which allow for a full set of teeth to be anchored securely.
   - **Partial Restoration**: If only certain areas of the jaw are missing teeth, fewer implants might be needed.
3. **Dentist's Recommendations**: Your dentist or oral surgeon will assess your individual situation, including your oral health, to determine the optimal number of implants.
4. **Personal Preference**: Some patients may opt for more implants for additional stability, while others may choose a minimal approach.

It's best to consult with a dental professional who can evaluate your specific needs and provide a tailored treatment plan.

B. To restore a completely edentulous (toothless) upper and lower jaw with dental implants, the number of implants required depends on the type of prosthetic restoration you choose. Here are the most common options:
1. **All-on-4 or All-on-6 Implant System**
- **All-on-4**: This method uses **4 implants** per jaw to support a full arch of fixed teeth.
- **All-on-6**: Uses **6 implants** for increased support and stability.
- Suitable for both upper and lower jaws.
- **Upper Jaw**: 4-6 implants.
- **Lower Jaw**: 4-6 implants.

This approach is ideal for full-arch restorations with a fixed prosthesis and offers a more cost-effective and minimally invasive solution.
2. **Implant-Supported Overdentures (Removable)**
- **Upper Jaw**: Typically **4-6 implants**.
- **Lower Jaw**: Usually **2-4 implants**, since the lower jaw has denser bone, allowing fewer implants.

This option provides a removable denture that attaches to implants for improved stability compared to traditional dentures.

Supplementary Table S1 (*Continued*)

3. **Full-Mouth Individual Crowns or Bridges**<br>- If you want individual crowns or bridges to replace each missing tooth, you might need **6-10 implants per jaw**, as placing one implant per tooth is often unnecessary.<br>The specific number of implants will depend on factors such as your bone density, aesthetic goals, and the type of prosthesis. Consulting with your dentist or prosthodontist will help determine the optimal solution for your case.<br>C. The number of dental implants needed to restore an edentulous upper and lower jaw depends on several factors, including:<br>1. **Jawbone Density**: The quality and quantity of bone available for implantation can influence the number of implants required. Bone grafting might be necessary if there is significant bone loss.<br>2. **Type of Restoration**:<br>- **Full Arch Restoration**: For a complete restoration of both the upper and lower jaw, you might need 4 to 6 implants per arch using techniques like All-on-4 or All-on-6, which allow for a full set of teeth to be anchored securely.<br>- **Partial Restoration**: If only certain areas of the jaw are missing teeth, fewer implants might be needed.<br>3. **Dentist's Recommendations**: Your dentist or oral surgeon will assess your individual situation, including your oral health, to determine the optimal number of implants.<br>4. **Personal Preference**: Some patients may opt for more implants for additional stability, while others may choose a minimal approach.<br>It's best to consult with a dental professional who can evaluate your specific needs and provide a tailored treatment plan.<br>**Q16: Do I need to replace my dental implants in the future?**<br>A. Dental implants are designed to be a long-term solution, and with proper care, they can last many years or even a lifetime. However, certain factors can influence the longevity of implants and whether they may need to be replaced:<br>1. **Oral Hygiene**: Good oral care, including brushing, flossing, and regular dental checkups, is essential for maintaining the health of your implants.<br>2. **Gum and Bone Health**: Conditions like gum disease (peri-implantitis) or bone loss around the implant can cause it to loosen, potentially leading to replacement.<br>3. **Implant Materials**: The quality of the implant material and the skill of the placement also play a role in durability. Well-placed implants with high-quality materials usually don't need to be replaced.<br>4. **Wear and Tear**: Although the titanium implant itself is highly durable, the crown (the artificial tooth) may wear down over time and may require replacement after 10–15 years.<br>5. **Lifestyle Factors**: Smoking or excessive teeth grinding (bruxism) can negatively impact implant longevity.<br>Routine dental visits are crucial to assess the condition of your implants and catch any potential issues early. Do you want to explore more about implant maintenance or risks associated with long-term use?<br>B. Dental implants are designed to be a long-lasting solution for tooth replacement. However, several factors can influence their longevity, including:<br>1. **Bone Health**: The health and density of the jawbone are crucial. If the bone deteriorates over time, it may affect the stability of the implant.<br>2. **Oral Hygiene**: Good oral hygiene practices are essential. Gum disease or peri-implantitis (inflammation around the implant) can lead to implant failure.<br>3. **Lifestyle Factors**: Smoking, excessive alcohol consumption, and poor diet can negatively impact the success of dental implants.<br>4. **Wear and Tear**: While the implant itself can last many years, the crown (the visible part of the implant) may wear out and might need replacement due to normal wear or damage.<br>5. **Regular Check-ups**: Regular dental check-ups can help monitor the condition of the implant and surrounding tissues, allowing for early intervention if issues arise.<br>In summary, while dental implants are meant to be permanent, they may require replacement or maintenance over time depending on various factors. It's best to consult your dentist or oral surgeon for personalized advice and regular assessments.<br>C. Dental implants are designed to be a long-term solution for tooth replacement, but their longevity can depend on several factors:<br>1. **Implant Material**: Most dental implants are made of titanium, which is biocompatible and resistant to corrosion, giving them a long lifespan.<br>2. **Bone Health**: The health and density of the jawbone supporting the implant are crucial. If bone loss occurs, it may lead to implant failure.<br>3. **Oral Hygiene**: Maintaining good oral hygiene is essential. Poor hygiene can lead to gum disease or peri-implantitis, which can compromise the implant's stability.<br>4. **Lifestyle Factors**: Habits like smoking or excessive alcohol consumption can negatively affect the success of implants.<br>5. **Regular Check-ups**: Routine dental visits help monitor the condition of the implants and surrounding tissues, allowing for early intervention if any issues arise.<br>6. **Crown Replacement**: While the implant itself may last a lifetime, the crown (the visible part of the implant) may wear out and need replacement due to normal wear, damage, or aesthetic reasons.<br>In general, dental implants can last many years, often a lifetime, with proper care and maintenance. However, it's essential to have regular check-ups with your dentist to assess the health of your implants and surrounding tissues. If you have any concerns about your specific situation, it's best to consult with your dental professional.<br>**Q17: Can I replace my complete denture with implant-supported dental prosthesis?**<br>A. Yes, you can replace your complete denture with an implant-supported dental prosthesis, often referred to as an implant-supported denture or overdenture. Here are some key points to consider:

(*Continued*)

Supplementary Table S1 (*Continued*)

1. **Bone Health**: Sufficient bone density and volume in the jaw are crucial for the placement of dental implants. If bone loss has occurred, you may need bone grafting procedures before implantation.
2. **Number of Implants**: Generally, two to four implants are required to support an implant-supported denture. The exact number will depend on your specific case and the design of the prosthesis.
3. **Benefits**: Implant-supported dentures can improve stability, retention, and function compared to traditional complete dentures. They can also help preserve jawbone health and prevent further bone loss.
4. **Consultation**: It's essential to consult with a dental professional or oral surgeon who specializes in implant dentistry. They can assess your oral health, discuss your options, and create a personalized treatment plan.
5. **Maintenance**: Proper oral hygiene and regular dental check-ups are essential for the longevity of implant-supported dentures.

If you're considering this option, discussing it with your dentist will provide you with tailored information and guidance based on your individual situation.
B. Yes, you can replace a complete denture with an implant-supported dental prosthesis. This transition can provide several benefits, including:

1. **Improved Stability and Retention**: Implant-supported prostheses are anchored directly to the jawbone, making them more stable than traditional dentures, which can slip or shift.
2. **Enhanced Comfort**: Many patients find implant-supported prostheses more comfortable than complete dentures, as they do not rely on adhesive and do not cover the palate.
3. **Better Functionality**: With implants, you may experience improved chewing ability and speech compared to complete dentures.
4. **Preservation of Jawbone**: Implants help stimulate the jawbone, preventing bone loss that often occurs with traditional dentures.

Considerations Before Making the Switch:
- **Bone Density**: Adequate bone density is necessary for implant placement. If bone loss has occurred, you may need a bone graft.
- **Oral Health**: Good overall oral health is essential for successful implantation.
- **Cost**: Implant-supported prostheses can be more expensive than traditional dentures, so it's important to consider your budget and insurance coverage.
- **Time and Treatment**: The process may take several months, including healing time after surgery and the placement of the prosthesis.

It's best to consult with a dental professional or oral surgeon who can assess your specific situation and recommend the most appropriate treatment plan.
C. Yes, you can replace a complete denture with an implant-supported dental prosthesis. This transition offers several advantages:

1. **Improved Stability**: Implant-supported dentures are anchored to the jawbone, providing greater stability and preventing slippage compared to traditional dentures.
2. **Enhanced Comfort**: Many patients find implant-supported options more comfortable, as they do not cover the palate and do not require adhesives.
3. **Better Functionality**: These prostheses often improve chewing efficiency and speech.
4. **Bone Preservation**: Implants help stimulate the jawbone, which can prevent the bone loss that often occurs with traditional dentures.

Key Considerations
- **Bone Density**: Adequate bone density is necessary for successful implant placement. If bone loss has occurred, you might need a bone graft.
- **Oral Health**: Good oral health is essential for successful implantation.
- **Cost**: Implant-supported prostheses tend to be more expensive than traditional dentures, so it's essential to consider your budget and insurance coverage.
- **Treatment Timeline**: The process may take several months, including healing time after surgery.

Consulting with a dental professional or oral surgeon is essential to evaluate your specific needs and determine the best treatment plan.

Q18: How should I care of my dental implant?

A. Caring for your dental implant is essential to ensure its longevity and maintain good oral health. Here are some tips for proper care:

1. **Maintain Oral Hygiene**:
   - Brush your teeth at least twice a day with a soft-bristle toothbrush and non-abrasive fluoride toothpaste.
   - Floss daily to remove plaque and food particles between your teeth and around the implant.
2. **Use Antimicrobial Mouthwash**:
   - Consider using an antimicrobial or antiseptic mouthwash to help reduce bacteria in your mouth, especially after meals.
3. **Regular Dental Check-ups**:
   - Schedule regular visits with your dentist (usually every 6 months) for professional cleanings and check-ups to monitor the health of your implant and surrounding tissues.
4. **Avoid Hard Foods**:
   - Be cautious with hard foods, as they can damage the implant or surrounding teeth. Cut food into smaller pieces and chew on the opposite side if necessary.

 © 2025. The Author(s).

Supplementary Table S1 (*Continued*)

5. **Quit Smoking**:
   - If you smoke, consider quitting, as smoking can hinder healing and increase the risk of implant failure.
6. **Monitor for Problems**:
   - Keep an eye out for signs of infection or complications, such as swelling, pain, or loosening of the implant. Contact your dentist if you notice any issues.
7. **Use a Nightguard if Necessary**:
   - If you grind your teeth at night (bruxism), consider using a nightguard to protect your implant and natural teeth.
8. **Healthy Diet**:
   - Maintain a balanced diet rich in vitamins and minerals to support overall oral health.

By following these care guidelines, you can help ensure the long-term success of your dental implant.

B. Caring for your dental implant is essential to ensure its longevity and maintain your overall oral health. Here are some key steps to follow:
Daily Care
1. **Brush Regularly**:
   - Brush your teeth at least twice a day with a soft-bristled toothbrush.
   - Be gentle around the implant area to avoid irritating the gums.
2. **Floss Daily**:
   - Use dental floss or an interdental brush to clean between your teeth and around the implant.
   - Consider using a floss threader if the implant is adjacent to natural teeth.
3. **Mouthwash**:
   - Rinse with an antibacterial mouthwash to help reduce plaque and bacteria.
Dietary Considerations
   - **Eat a Balanced Diet**: Incorporate foods rich in vitamins and minerals to support healing.
   - **Avoid Hard or Sticky Foods**: These can put unnecessary pressure on the implant and may cause damage.
Regular Dental Visits
   - **Routine Check-ups**: Schedule regular dental visits (usually every six months) to monitor the implant's health.
   - **Professional Cleanings**: Get professional cleanings to remove plaque and tartar buildup.
Monitor for Issues
   - **Watch for Signs of Infection**: Be alert for swelling, redness, pain, or pus around the implant site.
   - **Check for Loose Implants**: If you notice any loosening of the implant, contact your dentist immediately.
Additional Tips
   - **Use a Night Guard**: If you grind your teeth, a night guard can protect the implant.
   - **Avoid Tobacco**: If you smoke, consider quitting, as it can hinder healing and the success of the implant.

By following these care guidelines, you can help ensure the success of your dental implant and maintain good oral health. If you have any specific concerns or questions, don't hesitate to reach out to your dentist for personalized advice.

C. Caring for your dental implant is crucial for its longevity and your overall oral health. Here are some tips for proper care:
Daily Oral Hygiene
1. **Brushing**: Brush your teeth at least twice a day with a soft-bristled toothbrush. Be gentle around the implant site to avoid irritation.
2. **Flossing**: Use dental floss or an interdental brush to clean between your teeth and around the implant. Specialized implant flossers are also available.
3. **Mouthwash**: Rinse with an antibacterial mouthwash to help reduce plaque and bacteria around the implant.
Regular Dental Visits
   - **Check-ups**: Schedule regular dental check-ups (typically every six months) to monitor the health of your implant and surrounding tissues.
   - **Professional Cleaning**: Professional dental cleanings can help maintain the health of your gums and implant.
Diet and Lifestyle
   - **Healthy Diet**: Eat a balanced diet rich in vitamins and minerals to support healing and oral health.
   - **Avoid Hard Foods**: Steer clear of hard or sticky foods that could damage your implant or surrounding teeth.
   - **Quit Smoking**: If you smoke, consider quitting, as smoking can negatively affect healing and implant success.
Recognize Problems Early
   - **Signs of Infection**: Be aware of signs of infection, such as swelling, redness, pain, or pus around the implant site. Contact your dentist if you notice any of these symptoms.
   - **Loose Implant**: If you feel your implant becoming loose, seek dental attention immediately.
Additional Tips
   - **Use a Night Guard**: If you grind your teeth at night, a night guard can help protect your implant.
   - **Avoid Whitening Products**: Some whitening products can irritate the gums or affect the implant materials.

Following these care guidelines can help ensure the success of your dental implant and maintain your oral health. If you have any specific concerns, it's always best to consult your dentist for personalized advice.

Q19: **How long does the entire dental implant process take?**

A. The entire dental implant process can take several months, typically ranging from 4 to 12 months, depending on various factors. Here's a general breakdown of the stages involved:
1. **Initial Consultation (1 visit)**: This involves a thorough examination, imaging (like X-rays or 3D scans), and discussing the treatment plan. This visit usually lasts about an hour.
2. **Implant Placement Surgery (1 visit)**: The actual surgery to place the implant typically takes 1 to 2 hours.

(*Continued*)

 © 2025. The Author(s).

Supplementary Table S1 (*Continued*)

3. **Healing Period (3 to 6 months)**: After the implant is placed, a healing period is necessary for osseointegration, where the bone grows around the implant, securing it in place. This period can vary based on individual healing rates and bone quality.
4. **Abutment Placement (1 visit)**: Once the implant has integrated, a minor surgery is performed to attach the abutment (the connector between the implant and the crown). This is usually a quick procedure and can be done in about an hour.
5. **Further Healing (2 weeks to 1 month)**: After the abutment placement, additional healing is needed before the final crown can be placed.
6. **Crown Placement (1 visit)**: Finally, the custom crown is attached to the abutment. This appointment usually takes about 1 hour.

Summary:
- **Total Time**: Approximately 4 to 12 months.
- **Factors Influencing Duration**:
- Individual healing capacity.
- Bone quality and quantity (bone grafting may be necessary).
- Overall health and any underlying medical conditions.

Patients are advised to maintain regular follow-ups with their dentist to monitor progress throughout the process.

B. The entire dental implant process can vary significantly depending on several factors, including the individual's oral health, the number of implants, and whether additional procedures (like bone grafting) are needed. Here's a general timeline:

1. **Initial Consultation**: 1 appointment (1-2 hours)
   - This includes a thorough examination and imaging (X-rays or CT scans) to assess the jawbone and overall dental health.
2. **Treatment Planning**: 1 appointment (1-2 weeks after initial consultation)
   - Detailed planning and scheduling of the procedure.
3. **Implant Placement Surgery**: 1 appointment (1-2 hours)
   - The dental implant is surgically placed into the jawbone.
4. **Healing Period (Osseointegration)**: 3 to 6 months
   - This is the time it takes for the implant to fuse with the bone. During this period, a temporary restoration may be placed if necessary.
5. **Abutment Placement**: 1 appointment (1 hour)
   - After healing, an abutment (connector) is placed on the implant, and healing usually takes an additional 1-2 weeks.
6. **Final Restoration**: 1-2 appointments (2-4 weeks)
   - Custom crowns or prostheses are fabricated and attached. This can take a few weeks, depending on laboratory fabrication time.

Total Time: Generally, the entire process can take anywhere from **5 to 12 months**, but some patients may experience a shorter or longer timeline based on their specific circumstances

C. The entire dental implant process can take several months, typically ranging from 4 to 12 months, depending on various factors. Here's a general breakdown of the stages involved:

1. **Initial Consultation (1 visit)**: This involves a thorough examination, imaging (like X-rays or 3D scans), and discussing the treatment plan. This visit usually lasts about an hour.
2. **Implant Placement Surgery (1 visit)**: The actual surgery to place the implant typically takes 1 to 2 hours.
3. **Healing Period (3 to 6 months)**: After the implant is placed, a healing period is necessary for osseointegration, where the bone grows around the implant, securing it in place. This period can vary based on individual healing rates and bone quality.
4. **Abutment Placement (1 visit)**: Once the implant has integrated, a minor surgery is performed to attach the abutment (the connector between the implant and the crown). This is usually a quick procedure and can be done in about an hour.
5. **Further Healing (2 weeks to 1 month)**: After the abutment placement, additional healing is needed before the final crown can be placed.
6. **Crown Placement (1 visit)**: Finally, the custom crown is attached to the abutment. This appointment usually takes about 1 hour.

Summary:
- **Total Time**: Approximately 4 to 12 months.
- **Factors Influencing Duration**:
- Individual healing capacity.
- Bone quality and quantity.
- Overall health and any underlying medical conditions.

Patients are advised to maintain regular follow-ups with their dentist to monitor progress throughout the process.

Q20: **Is it possible to get "Metal-Free" implants?**

A. Yes, it is possible to get "metal-free" dental implants. These implants are typically made from materials such as zirconia, which is a ceramic material that offers several advantages over traditional titanium implants. Here are some key points about metal-free implants:

1. **Material Composition**: Zirconia implants are the most common type of metal-free implant. They are known for their strength and biocompatibility, making them a suitable option for many patients.
2. **Benefits**:
   - **Biocompatibility**: Zirconia is generally well tolerated by the body, reducing the risk of allergic reactions.
   - **Aesthetic Appeal**: These implants can blend in more naturally with surrounding teeth due to their tooth-like color, making them an attractive option for anterior (front) teeth.
   - **Reduced Sensitivity**: Zirconia implants have lower thermal conductivity than metal implants, which may result in less sensitivity to temperature changes.

 © 2025. The Author(s).

Supplementary Table S1 (*Continued*)

3. **Durability**: While zirconia implants are durable, titanium implants have a long track record of proven success and superior mechanical strength. Recent advancements in zirconia technology have improved their strength and reliability.
4. **Surgical Considerations**: The procedure for placing metal-free implants is similar to that for titanium implants. However, the specific techniques and healing times may vary, so it's important to consult with a dental professional experienced in placing zirconia implants.
5. **Long-term Success**: Research on the long-term success and performance of metal-free implants is ongoing. While some studies show promising results, more data is needed to fully understand their longevity compared to titanium implants.

If you're considering metal-free implants, it's essential to discuss your options with your dentist or oral surgeon. They can help determine the best solution for your specific dental needs and preferences.
B. Yes, metal-free dental implants are available and are often made from materials like zirconia (a type of ceramic). Here's an overview of metal-free implants:
Advantages of Metal-Free Implants:
1. **Biocompatibility**: Zirconia is highly biocompatible, which means it is less likely to cause allergic reactions or inflammation in the surrounding tissues.
2. **Aesthetics**: Metal-free implants can provide a more natural appearance, especially for front teeth, as they can be more similar in color to natural teeth.
3. **Corrosion Resistance**: Unlike metal implants, zirconia does not corrode, which can be beneficial in maintaining the implant's integrity over time.
4. **Less Heat Conduction**: Zirconia has lower thermal conductivity, which can make it more comfortable for some patients.
Disadvantages:
1. **Limited Long-Term Research**: While zirconia implants have shown promising results, they may not have the extensive long-term data available for traditional titanium implants.
2. **Potential for Fracture**: Zirconia is less flexible than titanium, which may make it more susceptible to fracture under certain conditions, particularly in patients with high bite forces.
3. **Cost**: Metal-free implants can sometimes be more expensive than traditional metal implants.
Indications:
Metal-free implants may be suitable for patients who:
- Have a known metal allergy or sensitivity.
- Prefer aesthetics over metal implants in visible areas.
- Are looking for alternative materials due to personal or health reasons.
Conclusion:
Metal-free implants, especially zirconia implants, can be a viable option for certain patients. It's essential to discuss with a dental professional to evaluate the best choice based on individual needs, health conditions, and aesthetic goals.
C. Yes, it is possible to get "metal-free" dental implants. These implants are most commonly made from **zirconia**, a strong ceramic material that offers a non-metal alternative to traditional titanium implants.
Key Features of Metal-Free (Zirconia) Implants:
1. **Material Composition**: Zirconia is a ceramic material known for its strength, biocompatibility, and aesthetic benefits. It is the primary material used in metal-free implants.
2. **Benefits**:
   - **Biocompatibility**: Zirconia is generally well-tolerated by the body, reducing the risk of allergic reactions, which makes it suitable for patients with metal allergies.
   - **Aesthetic Appeal**: Zirconia implants are tooth-colored, which allows them to blend more naturally with the surrounding teeth and gums. This is especially advantageous in the front teeth or for patients with thin gums where metal might show through.
   - **Reduced Sensitivity**: Unlike metals, zirconia has lower thermal conductivity, meaning it can reduce temperature sensitivity in the mouth.
3. **Durability**: Zirconia implants are strong and durable, but titanium implants still have a longer track record and superior mechanical strength. However, advancements in zirconia implant technology are improving their reliability.
4. **Surgical Considerations**: The surgical process for placing zirconia implants is similar to that of titanium implants. Healing times and procedures might slightly vary, but a dentist with experience in zirconia implants can guide the best approach.
5. **Long-Term Success**: Although zirconia implants are gaining popularity, more long-term studies are needed to compare their longevity with titanium implants, which have decades of proven success.
Ideal Candidates:
- Patients with allergies or sensitivities to metal.
- Those seeking a more natural aesthetic appearance.
- People who prefer a non-metal option for health or personal reasons.

If you're considering metal-free implants, consult with your dentist to discuss the pros and cons based on your specific needs.

<u>Copilot/Bing</u>

Q1.What is a dental implant?
A. A dental implant is a medical device used to replace missing teeth. It consists of a titanium screw that is surgically inserted into the jawbone, acting as an artificial tooth root. Over time, the bone fuses with the implant, securing it firmly in place. Once the implant is stable, a crown (a false tooth) is attached to it, which is designed to match the color and shape of your natural teeth.
Dental implants offer several benefits:
- Natural appearance and function: They look and feel like natural teeth.

(*Continued*)

 © 2025. The Author(s).

Supplementary Table S1 (*Continued*)

- Durability: With proper care, they can last many years.
- Improved oral health: They help maintain the jawbone's integrity and prevent the shifting of adjacent teeth.

If you have any more questions about dental implants or need further details, feel free to ask!

B. A dental implant is a medical device used to replace missing teeth. It consists of a titanium screw that is surgically inserted into the jawbone, acting as an artificial tooth root. Over time, the bone fuses with the implant, securing it firmly in place. Once the implant is stable, a crown (a false tooth) is attached to it, which is designed to match the color and shape of your natural teeth.
Dental implants offer several benefits:

- Natural appearance and function: They look and feel like natural teeth.
- Durability: With proper care, they can last many years.
- Improved oral health: They help maintain the jawbone's integrity and prevent the shifting of adjacent teeth.

If you have any more questions about dental implants or need further details, feel free to ask!

C. A dental implant is a medical device used to replace missing teeth. It involves a titanium screw that is surgically placed into the jawbone, acting as an artificial tooth root. Over time, the bone integrates with the implant, securing it firmly in place. Once the implant is stable, a crown (a false tooth) is attached to it, designed to match the color and shape of your natural teeth.
Dental implants offer several benefits:

- Natural appearance and function: They look and feel like natural teeth.
- Durability: With proper care, they can last many years.
- Improved oral health: They help maintain the jawbone's integrity and prevent the shifting of adjacent teeth.

If you have any more questions about dental implants or need further details, feel free to ask!

Q2. Is everyone eligible to get a dental implant?

A. Not everyone is eligible for dental implants. Eligibility depends on several factors, including:

1. Overall Health: Candidates should be in good general health. Conditions like uncontrolled diabetes or heart disease can affect healing and implant success.
2. Oral Health: Good oral hygiene and healthy gums are crucial. Gum disease or other oral infections need to be treated before implant surgery.
3. Bone Density: Adequate jawbone density is necessary to support the implant. Bone grafting might be required if there's insufficient bone.
4. Non-Smokers: Smoking can impede healing and increase the risk of implant failure. Non-smokers are generally better candidates.
5. Age: While there's no upper age limit, younger patients whose jawbones are still growing may not be suitable candidates.

It's best to consult with a dental professional to determine if dental implants are the right option for you. Do you have any specific concerns or conditions you're wondering about?

B. Not everyone is eligible for dental implants. Eligibility depends on several factors, including:

1. Overall Health: Candidates should be in good general health. Conditions like uncontrolled diabetes or heart disease can affect healing and implant success.
2. Oral Health: Good oral hygiene and healthy gums are crucial. Gum disease or other oral infections need to be treated before implant surgery.
3. Bone Density: Adequate jawbone density is necessary to support the implant. Bone grafting might be required if there's insufficient bone.
4. Non-Smokers: Smoking can impede healing and increase the risk of implant failure. Non-smokers are generally better candidates.
5. Age: While there's no upper age limit, younger patients whose jawbones are still growing may not be suitable candidates

It's best to consult with a dental professional to determine if dental implants are the right option for you. Do you have any specific concerns or conditions you're wondering about?

C. Not everyone is eligible for dental implants. Eligibility depends on several factors, including:

1. Overall Health: Candidates should be in good general health. Conditions like uncontrolled diabetes or heart disease can affect healing and implant success.
2. Oral Health: Good oral hygiene and healthy gums are crucial. Gum disease or other oral infections need to be treated before implant surgery.
3. Bone Density: Adequate jawbone density is necessary to support the implant. Bone grafting might be required if there's insufficient bone.
4. Non-Smokers: Smoking can impede healing and increase the risk of implant failure. Non-smokers are generally better candidates.
5. Age: While there's no upper age limit, younger patients whose jawbones are still growing may not be suitable candidates.

It's best to consult with a dental professional to determine if dental implants are the right option for you. Do you have any specific concerns or conditions you're wondering about?

3. As a diabetic patient, will a dental implant be successful for me?

A. Yes, dental implants can be successful for diabetic patients, but there are some important considerations to keep in mind.
Success Rates: Studies show that the success rate for dental implants in well-controlled diabetic patients is quite high, ranging from 88.8% to 97.3% one year after placement. This is comparable to the success rates in non-diabetic patients.
Key Factors:

1. Blood sugar control: Maintaining good blood sugar control is crucial. Poorly controlled diabetes can increase the risk of complications such as infections and slower healing.
2. Regular check-ups: Frequent dental visits and close monitoring by both your dentist and your healthcare provider can help manage any potential risks.

 © 2025. The Author(s).

Supplementary Table S1 (*Continued*)

3. Oral hygiene: Good oral hygiene practices are essential to prevent infections and ensure the longevity of the implant.

Precautions:

- Presurgical assessment: Your dentist will likely conduct a thorough assessment to ensure you are a suitable candidate for the procedure.
- Post-Surgical Care: following the dentist's postoperative care instructions carefully will help in the healing process and reduce the risk of complications.

If you have any concerns, it's always best to discuss them with your dentist and healthcare provider to ensure you receive personalized advice and care. How are you feeling about the possibility of getting a dental implant?

B. Yes, dental implants can be successful for diabetic patients, but there are some important considerations to keep in mind.

Success Rates: Studies show that the success rate for dental implants in well-controlled diabetic patients is quite high, ranging from 88.8% to 97.3% one year after placement. This is comparable to the success rates in non-diabetic patients.

Key Factors:

1. Blood sugar control: Maintaining good blood sugar control is crucial. Poorly controlled diabetes can increase the risk of complications such as infections and slower healing.
2. Regular check-ups: Frequent dental visits and close monitoring by both your dentist and your healthcare provider can help manage any potential risks.
3. Oral hygiene: Good oral hygiene practices are essential to prevent infections and ensure the longevity of the implant.

Precautions:

- Presurgical assessment: Your dentist will likely conduct a thorough assessment to ensure you are a suitable candidate for the procedure.
- Postsurgical care: Following the dentist's post-operative care instructions carefully will help in the healing process and reduce the risk of complications.

If you have any concerns, it's always best to discuss them with your dentist and healthcare provider to ensure you receive personalized advice and care. How are you feeling about the possibility of getting a dental implant?

C. Yes, dental implants can be successful for diabetic patients, but there are some important considerations to keep in mind.

Success rates: Studies show that the success rate for dental implants in well-controlled diabetic patients is quite high, ranging from 88.8% to 97.3% one year after placement. This is comparable to the success rates in nondiabetic patients.

Key Factors:

1. Blood sugar control: Maintaining good blood sugar control is crucial. Poorly controlled diabetes can increase the risk of complications such as infections and slower healing.
2. Regular check-ups: Frequent dental visits and close monitoring by both your dentist and your healthcare provider can help manage any potential risks.
3. Oral hygiene: Good oral hygiene practices are essential to prevent infections and ensure the longevity of the implant.

Precautions:

- Presurgical assessment: Your dentist will likely conduct a thorough assessment to ensure you are a suitable candidate for the procedure.
- Postsurgical care: Following the dentist's post-operative care instructions carefully will help in the healing process and reduce the risk of complications.

If you have any concerns, it's always best to discuss them with your dentist and healthcare provider to ensure you receive personalized advice and care. How are you feeling about the possibility of getting a dental implant?

4. What impact does smoking have on dental implants?

A. Smoking can significantly impact the success and longevity of dental implants. Here are some key points to consider:

1. Increased risk of implant failure: Smokers have a higher rate of dental implant failure compared to non-smokers. This is due to the negative effects of nicotine and other chemicals in cigarettes, which impair blood flow and healing.
2. Impaired healing: Smoking affects the body's ability to heal after surgery. This can lead to complications during the osseointegration process, where the implant fuses with the jawbone.
3. Higher risk of infections: Smokers are more prone to infections such as peri-implantitis, an inflammatory condition that affects the tissues around the implant. This can lead to bone loss and implant failure.
4. Reduced Success Rates: Overall, the success rate of dental implants is lower in smokers. Studies have shown a dose-response relationship, meaning the more a person smokes, the higher the risk of implant failure
5. Long-term complications: Even after successful implantation, smokers may face long-term complications that can affect the stability and health of the implant.

Quitting smoking before and after getting dental implants can significantly improve the chances of a successful outcome. If you're considering dental implants and are a smoker, discussing smoking cessation strategies with your dentist can be very beneficial.

Is there anything else you'd like to know about dental implants or oral health?

B. Smoking can have several detrimental effects on dental implants. Here are some key impacts:

1. Higher failure rates: Smokers experience a higher rate of dental implant failure compared to non-smokers. This is due to the negative effects of nicotine and other chemicals in cigarettes, which impair blood flow and healing.
2. Impaired healing: Smoking affects the body's ability to heal after surgery. This can lead to complications during the osseointegration process, where the implant fuses with the jawbone.
3. Increased risk of infections: Smokers are more prone to infections such as peri-implantitis, an inflammatory condition that affects the tissues around the implant. This can lead to bone loss and implant failure.
4. Reduced success rates: Overall, the success rate of dental implants is lower in smokers. Studies have shown a dose-response relationship, meaning the more a person smokes, the higher the risk of implant failure.

(*Continued*)

 © 2025. The Author(s).

Supplementary Table S1 (*Continued*)

5. Long-term complications: Even after successful implantation, smokers may face long-term complications that can affect the stability and health of the implant.

Quitting smoking before and after getting dental implants can significantly improve the chances of a successful outcome. If you're considering dental implants and are a smoker, discussing smoking cessation strategies with your dentist can be very beneficial.
Is there anything else you'd like to know about dental implants or oral health?
C. Smoking can significantly affect the success and longevity of dental implants. Here are some key impacts:

1. Higher failure rates: Smokers have a higher rate of dental implant failure compared to non-smokers. Nicotine and other chemicals in cigarettes impair blood flow and healing, which are crucial for the success of implants.
2. Impaired healing: Smoking affects the body's ability to heal after surgery. This can lead to complications during the osseointegration process, where the implant fuses with the jawbone.
3. Increased risk of infections: Smokers are more prone to infections such as peri-implantitis, an inflammatory condition that affects the tissues around the implant. This can lead to bone loss and implant failure.
4. Reduced success rates: Overall, the success rate of dental implants is lower in smokers. Studies have shown a dose-response relationship, meaning the more a person smokes, the higher the risk of implant failure.
5. Long-term complications: Even after successful implantation, smokers may face long-term complications that can affect the stability and health of the implant.

Quitting smoking before and after getting dental implants can significantly improve the chances of a successful outcome. If you're considering dental implants and are a smoker, discussing smoking cessation strategies with your dentist can be very beneficial.
Is there anything else you'd like to know about dental implants or oral health?
Q5. How soon after a tooth extraction can an implant be placed?
A. The timing for placing a dental implant after a tooth extraction can vary based on several factors, including the condition of the tooth and surrounding bone, as well as your overall oral health. Here are the general options:

1. Immediate implant placement: In some cases, an implant can be placed immediately after the tooth is extracted. This is often referred to as "immediate implant placement." It is typically done when there is no infection or significant bone loss.
2. Early implant placement: This usually occurs within 2-3 months after extraction. This allows time for the initial healing of the gum and bone.
3. Delayed implant placement: This is done after a longer healing period, usually 4–6 months or more. This option is chosen when there is a need for significant bone healing or if bone grafting is required.

The best approach depends on your specific situation, so it's important to consult with your dentist or oral surgeon to determine the most suitable timing for your implant placement.
Do you have any other questions about dental implants or oral health?
B. The timing for placing a dental implant after a tooth extraction can vary based on several factors, including the condition of the tooth and surrounding bone, as well as your overall oral health. Here are the general options:

1. Immediate implant placement: In some cases, an implant can be placed immediately after the tooth is extracted. This is often referred to as "immediate implant placement." It is typically done when there is no infection or significant bone loss.
2. Early implant placement: This usually occurs within 2-3 months after extraction. This allows time for the initial healing of the gum and bone.
3. Delayed pmplant placement: This is done after a longer healing period, usually 4–6 months or more. This option is chosen when there is a need for significant bone healing or if bone grafting is required.

The best approach depends on your specific situation, so it's important to consult with your dentist or oral surgeon to determine the most suitable timing for your implant placement.
Do you have any other questions about dental implants or oral health?
C. The timing for placing a dental implant after a tooth extraction can vary based on several factors, including the condition of the tooth and surrounding bone, as well as your overall oral health. Here are the general options:

1. Immediate implant placement: Sometimes, an implant can be placed right after the tooth is extracted. This is known as "immediate implant placement" and is typically done when there is no infection or significant bone loss.
2. Early implant placement: This usually happens within 2-3 months after extraction, allowing time for the initial healing of the gum and bone.
3. Delayed Implant Placement: This occurs after a longer healing period, usually 4-6 months or more. This option is chosen when significant bone healing or bone grafting is needed.

The best approach depends on your specific situation, so it's important to consult with your dentist or oral surgeon to determine the most suitable timing for your implant placement.
Do you have any other questions about dental implants or oral health?
Q6. Will the crown be attached right after the implant procedure?
A. Typically, the crown is not attached immediately after the implant procedure. After the dental implant is placed, there is usually a healing period of several months to allow the implant to integrate with the jawbone, a process called osseointegration. Once the implant has fully integrated, the crown can then be attached.
Do you have any other questions about the implant process?
B. Typically, the crown is not attached immediately after the implant procedure. The process usually involves several stages:

1. Implant placement: The dental implant is surgically placed into the jawbone.
2. Healing period: The implant needs time to integrate with the jawbone, a process called osseointegration, which can take several months.
3. Abutment placement: Once the implant has integrated, an abutment is attached to the implant. This serves as the connector between the implant and the crown.

Supplementary Table S1 (*Continued*)

4. Crown attachment: Finally, the crown is attached to the abutment. This can be done using either screws or dental cement. In some cases, a temporary crown may be placed during the healing period to protect the implant and maintain aesthetics.
C. The process of getting a dental implant typically involves several stages:
1. Implant placement: The dental implant is surgically placed into the jawbone.
2. Healing period: The implant needs time to integrate with the jawbone, a process called osseointegration, which can take several months.
3. Abutment placement: Once the implant has integrated, an abutment is attached to the implant. This serves as the connector between the implant and the crown.
4. Crown attachment: Finally, the crown is attached to the abutment. This can be done using either screws or dental cement. In some cases, a temporary crown may be placed during the healing period to protect the implant and maintain aesthetics.

7. How is the recovery after the dental implant placment procedure?

A. Recovery after a dental implant procedure can vary, but here are some general stages and tips to help you understand what to expect:

Immediate aftercare
- Rest: You'll need to rest until the effects of the anesthesia wear off, which might take several hours.
- Bleeding: Some light bleeding is normal and should ease within the first day or two.
- Pain management: Over-the-counter pain relief like paracetamol or ibuprofen can help manage any discomfort.

First few days
- Swelling and bruising: It's common to experience some swelling or bruising around the implant site. Applying an ice pack for 10 minutes every hour can help reduce this.
- Diet: Stick to soft foods and avoid hot food and drinks for the first 24 hours.
- Oral hygiene: Keep your mouth clean by rinsing with a recommended mouthwash several times a day, starting 24 hours after the procedure.

First few weeks
- Activity: Avoid strenuous activities for a few days after the surgery.
- Follow-up: Attend all follow-up appointments with your dentist to ensure proper healing.

Long-term healing
- Osseointegration: This is the process where the implant fuses with the jawbone, which can take several months.
- Care: Continue to follow your dentist's advice on oral hygiene and care to ensure the implant heals successfully.

If you have any specific concerns or experience unusual symptoms, it's always best to contact your dentist.
How are you feeling about the procedure? Do you have any specific concerns or questions?

B. Recovery after a dental implant procedure typically involves several stages, each with its own set of expectations and care requirements:
1. Immediate postsurgery:
   - First 24-48 hours: You might experience swelling, bruising, minor bleeding, and discomfort. It's important to rest and avoid strenuous activities.
   - Pain management: Over-the-counter pain relief like ibuprofen or paracetamol can help manage discomfort.
2. First Week:
   - Diet: Stick to soft foods and avoid hot drinks and foods.
   - Oral hygiene: Keep your mouth clean by rinsing with a recommended mouthwash and avoid touching the implant site with your fingers or tongue.
3. Osseointegration phase:
   - Duration: This critical healing phase, where the implant fuses with the jawbone, can take 3-6 months. During this time, follow your dentist's instructions carefully to ensure successful integration.
4. Mid-term healing:
   - Check-ups: Regular dental visits are essential to monitor the healing process and address any issues.
   - Activity: Gradually resume normal activities, but avoid anything too strenuous for the first few weeks.
5. Final restoration:
   - Prosthesis fitting: Once osseointegration is complete, the final crown, bridge, or denture is fitted. This stage may involve additional appointments to ensure a perfect fit.
6. Long-term care:
   - Maintenance: Good oral hygiene and regular dental check-ups are crucial to maintain the health and longevity of your dental implants.

Recovery times can vary based on individual health, the number of implants, and the complexity of the procedure. Always follow your dentist's specific advice for the best outcome.
If you have any specific concerns or need more detailed advice, feel free to ask!

C. Recovery after a dental implant procedure involves several stages, each with specific care requirements:
1. Immediate postsurgery:
   - First 24–48 hours: You might experience swelling, bruising, minor bleeding, and discomfort. Rest and avoid strenuous activities during this period.
   - Pain management: Over-the-counter pain relief like ibuprofen or paracetamol can help manage discomfort.
2. First week:
   - Diet: Stick to soft foods and avoid hot drinks and foods.
   - Oral hygiene: Keep your mouth clean by rinsing with a recommended mouthwash and avoid touching the implant site with your fingers or tongue.

(*Continued*)

Supplementary Table S1 (*Continued*)

3. Osseointegration phase:
   - Duration: This critical healing phase, where the implant fuses with jawbone, can take 3-6 months. Follow your dentist's instructions carefully to ensure successful integration. Follow your dentist's instructions carefully to ensure successful integration.
4. Mid-term healing:
   - Check-ups: Regular dental visits are essential to monitor the healing process and address any issues.
   - Activity: Gradually resume normal activities, but avoid anything too strenuous for the first few weeks.
5. Final restoration:
   - Prosthesis fitting: Once osseointegration is complete, the final crown, bridge, or denture is fitted. This stage may involve additional appointments to ensure a perfect fit.
6. Long-term care:
   - Maintenance: Good oral hygiene and regular dental check-ups are crucial to maintain the health and longevity of your dental implants.

Recovery times can vary based on individual health, the number of implants, and the complexity of the procedure. Always follow your dentist's specific advice for the best outcome.
If you have any specific concerns or need more detailed advice, feel free to ask!

8. Which is a better option to restore a missing tooth FPD or dental implant?

A. Choosing between a fixed partial denture (FPD) and a dental implant depends on various factors, including your specific dental needs, overall health, and personal preferences. Here are some key points to consider for each option:
Dental Implants
Pros:
- Longevity and durability: Implants are known for their long lifespan and durability, often lasting a lifetime with proper care.
- Bone preservation: They help preserve the jawbone, preventing bone loss that can occur with missing teeth.
- Natural look and feel: Implants look and feel like natural teeth, providing better aesthetics and comfort.

Cons:
- Cost: Implants can be more expensive upfront compared to FPDs.
- Surgical procedure: The process involves surgery and a longer healing period.
- Time: The entire process can take several months, from implant placement to final restoration.

Fixed partial dentures (FPDs)
Pros:
- Less invasive: FPDs do not require surgery and can be completed more quickly.
- Cost-effective: Generally, FPDs are less expensive than implants.
- Immediate Results: The process is faster, providing quicker restoration of function and aesthetics.

Cons:
- Impact on adjacent teeth: FPDs require the adjacent teeth to be filed down to support the bridge, which can affect their health.
- Bone loss: Unlike implants, FPDs do not prevent bone loss in the jaw.
- Durability: FPDs may not last as long as implants and might need replacement over time.

Decision factors
- Health of adjacent teeth: If the adjacent teeth are healthy, an implant might be a better option to avoid altering them.
- Bone density: Adequate bone density is required for implants. If bone loss is significant, additional procedures like bone grafting might be necessary.
- Budget: Consider your budget and whether you can afford the higher initial cost of implants.
- Time: If you need a quicker solution, FPDs might be more suitable.

It's best to consult with your dentist to evaluate your specific situation and determine the most appropriate option for you. They can provide personalized advice based on your dental health, preferences, and financial considerations.
Do you have any other questions or need further details on any of these points?

B. Choosing between a fixed partial denture (FPD) and a dental implant depends on several factors, including your dental health, budget, and personal preferences. Here's a comparison to help you decide:
Dental implants
Pros:
- Longevity and durability: Implants can last a lifetime with proper care.
- Bone preservation: They help maintain jawbone density, preventing bone loss.
- Natural look and feel: Implants look and function like natural teeth.

Cons:
- Cost: Higher initial cost compared to FPDs.
- Surgical procedure: Requires surgery and a longer healing period.
- Time: The process can take several months from start to finish.

Fixed partial dentures (FPDs)
Pros:
- Less invasive: No surgery required, and the procedure is quicker.
- Cost-effective: Generally less expensive than implants.
- Immediate results: Faster restoration of function and aesthetics.

Cons:
- Impact on adjacent teeth: Requires filing down adjacent teeth, which can affect their health.

 © 2025. The Author(s).

Supplementary Table S1 (*Continued*)

• Bone loss: Does not prevent bone loss in the jaw.<br>• Durability: May need replacement over time, unlike implants.<br>Decision factors<br>• Health of adjacent teeth: If adjacent teeth are healthy, an implant might be better to avoid altering them.<br>• Bone density: Adequate bone density is needed for implants. If bone loss is significant, additional procedures like bone grafting might be necessary.<br>• Budget: Consider your budget and whether you can afford the higher initial cost of implants.<br>• Time: If you need a quicker solution, FPDs might be more suitable.<br>Consulting with your dentist is crucial to evaluate your specific situation and determine the best option for you. They can provide personalized advice based on your dental health, preferences, and financial considerations.<br>Do you have any other questions or need further details on any of these points?<br>C. Choosing between a fixed partial denture (FPD) and a dental implant depends on various factors, including your dental health, budget, and personal preferences. Here's a comparison to help you decide:<br>Dental implants<br>Pros:<br>• Longevity and durability: Implants can last a lifetime with proper care.<br>• Bone preservation: They help maintain jawbone density, preventing bone loss.<br>• Natural look and feel: Implants look and function like natural teeth,<br>Cons:<br>• Cost: Higher initial cost compared to FPDs.<br>• Surgical procedure: Requires surgery and a longer healing period.<br>• Time: The process can take several months from start to finish.<br>Fixed partial dentures (FPDs)<br>Pros:<br>• Less invasive: No surgery required, and the procedure is quicker.<br>• Cost-effective: Generally less expensive than implants.<br>• Immediate results: Faster restoration of function and aesthetics.<br>Cons:<br>• Impact on adjacent teeth: Requires filing down adjacent teeth, which can affect their health.[1]<br>• Bone loss: Does not prevent bone loss in the jaw.<br>• Durability: May need replacement over time, unlike implants.<br>Decision factors<br>• Health of adjacent teeth: If adjacent teeth are healthy, an implant might be better to avoid altering them.<br>• Bone density: Adequate bone density is needed for implants. If bone loss is significant, additional procedures like bone grafting might be necessary.<br>• Budget: Consider your budget and whether you can afford the higher initial cost of implants.<br>• Time: If you need a quicker solution, FPDs might be more suitable.<br>Consulting with your dentist is crucial to evaluate your specific situation and determine the best option for you. They can provide personalized advice based on your dental health, preferences, and financial considerations.<br>Do you have any other questions or need further details on any of these points?<br>**9. Which is the better option: root canal treatment (RCT) or tooth extraction followed by an implant?**<br>A. Choosing between a root canal treatment (RCT) and tooth extraction followed by an implant depends on several factors, including the condition of the tooth, overall dental health, and personal preferences. Here's a comparison to help you decide:<br>Root canal treatment (RCT)<br>Pros:<br>• Preserves natural tooth: RCT saves your natural tooth by removing the infected pulp and sealing it.<br>• Less invasive: The procedure is less invasive compared to extraction and implant placement.<br>• Cost-effective: Generally, RCT is less expensive than getting an implant.<br>• Quicker recovery: Recovery time is usually shorter compared to the implant process.<br>Cons:<br>• Potential for future issues: There is a possibility of the tooth becoming re-infected, which might require additional treatment.<br>• Weakened tooth: The treated tooth may become more brittle and might need a crown for protection.<br>Tooth extraction followed by implant<br>Pros:<br>• Longevity and durability: Implants can last a lifetime with proper care.<br>• Bone preservation: Implants help maintain jawbone density, preventing bone loss.<br>• Natural look and feel: Implants look and function like natural teeth.<br>Cons:<br>• Cost: Higher initial cost compared to RCT.<br>• Surgical procedure: Requires surgery and a longer healing period.<br>• Time: The process can take several months from extraction to final implant placement.<br>Decision factors<br>• Condition of the tooth: If the tooth can be saved and has a good chance of long-term success, RCT is usually preferred. If the tooth is too damaged, extraction and implant might be necessary.

(*Continued*)

 © 2025. The Author(s).

Supplementary Table S1 (*Continued*)

- Overall health: Your overall health and bone density can influence the success of an implant.
- Budget: Consider your budget and whether you can afford the higher initial cost of an implant.
- Time: If you need a quicker solution, RCT might be more suitable.

Consulting with your dentist is crucial to evaluate your specific situation and determine the best option for you. They can provide personalized advice based on your dental health, preferences, and financial considerations.
Do you have any other questions or need further details on any of these points?
B. Choosing between a root canal treatment (RCT) and tooth extraction followed by an implant depends on several factors, including the condition of the tooth, overall oral health, and personal preferences. Here are some key points to consider:
Root canal treatment (RCT)

- Preservation of natural tooth: RCT aims to save your natural tooth by removing the infected pulp and sealing the tooth. This helps maintain the tooth’s structure and function.
- Less invasive: The procedure is generally less invasive compared to extraction and implant placement.
- Cost and time: RCT is often less expensive and quicker than the process of extraction and implant placement.
- Success rate: Root canals have a high success rate and can last a lifetime with proper care.

Tooth extraction and implant

- Severe damage: If the tooth is too damaged or decayed to be saved, extraction followed by an implant might be necessary.
- Durability: Dental implants are highly durable and can last many years, often providing a permanent solution.
- Aesthetic and functional benefits implant can restore the appearance and function of the tooth often indistinguishable from natural teeth.
- Longer Process: The process involves multiple stages, including extraction, healing, and implant placement, which can take several months.

Considerations

- Oral health: The overall health of your gums and jawbone can influence the decision. Healthy gums and sufficient bone density are crucial for successful implants.
- Personal preference: Some people prefer to keep their natural teeth whenever possible, while others might opt for implants for their durability and aesthetic benefits.

It’s best to discuss your specific situation with your dentist, who can provide personalized advice based on a thorough examination and your dental history. They can help you weigh the pros and cons of each option to make the best decision for your oral health.
Do you have any specific concerns or questions about either procedure?
C. Choosing between a root canal treatment (RCT) and tooth extraction followed by an implant depends on several factors, including the condition of the tooth, overall oral health, and personal preferences. Here are some key points to consider:
Root canal treatment (RCT)

- Preservation of natural tooth: RCT aims to save your natural tooth by removing the infected pulp and sealing the tooth. This helps maintain the tooth’s structure and function.
- Less invasive: The procedure is generally less invasive compared to extraction and implant placement.
- Cost and time: RCT is often less expensive and quicker than the process of extraction and implant placement.
- Success rate: Root canals have a high success rate and can last a lifetime with proper care.

Tooth extraction and implant

- Severe damage: If the tooth is too damaged or decayed to be saved, extraction followed by an implant might be necessary.
- Durability: Dental implants are highly durable and can last many years, often providing a permanent solution.
- Aesthetic and functional benefits: Implants can restore the appearance and function of the tooth, often indistinguishable from natural teeth.
- <u>Longer process: The process involves multiple stages, including extraction, healing, and implant placement, which can take several months</u>.

Considerations

- Oral health: The overall health of your gums and jawbone can influence the decision. <u>Healthy gums and sufficient bone density are crucial for successful implants[1]</u>.
- <u>Personal preference: Some people prefer to keep their natural teeth whenever possible, while others might opt for implants for their durability and aesthetic benefits</u>.

It’s best to discuss your specific situation with your dentist, who can provide personalized advice based on a thorough examination and your dental history. They can help you weigh the pros and cons of each option to make the best decision for your oral health.
Do you have any specific concerns or questions about either procedure?
10. Can the body reject a dental implant?
A. Yes, the body can reject a dental implant, but it’s quite rare. Dental implants are typically made from titanium, which is generally well-tolerated by the body. However, in some cases, complications can arise that may lead to implant failure. Here are some key points:

1. Titanium allergy: A very small percentage of people may have an allergy to titanium, which can cause the body to reject the implant.
2. Infection: If the implant site becomes infected, it can lead to implant failure.
3. Poor osseointegration: This is when the implant does not properly fuse with the jawbone, causing it to become loose.
4. Gum recession: If the gums recede around the implant, it can expose the implant and lead to failure.
5. Mechanical issues: Problems with the implant itself, such as improper placement or damage, can also cause failure.

Early symptoms of potential rejection or failure include persistent pain, swelling, inflammation, and bleeding around the implant site. If you experience any of these symptoms, it’s important to contact your dentist promptly.

Supplementary Table S1 (*Continued*)

Do you have any specific concerns about dental implants?
B. Yes, the body can reject a dental implant, but it's quite rare. Dental implants are typically made from titanium, which is generally well-tolerated by the body. However, there are a few reasons why an implant might fail:

1. Titanium allergy: A very small percentage of people may have an allergy to titanium, which can cause the body to reject the implant.
2. Infection: If the implant site becomes infected, it can lead to implant failure.
3. Poor osseointegration: This is when the implant does not properly fuse with the jawbone, causing it to become loose.
4. Gum recession: If the gums recede around the implant, it can expose the implant and lead to failure.
5. Mechanical issues: Problems with the implant itself, such as improper placement or damage, can also cause failure.

Early symptoms of potential rejection or failure include persistent pain, swelling, inflammation, and bleeding around the implant site. If you experience any of these symptoms, it's important to contact your dentist promptly.
Do you have any specific concerns about dental implants?
C. Yes, the body can reject a dental implant, but it's quite rare. Dental implants are typically made from titanium, which is generally well-tolerated by the body. However, there are a few reasons why an implant might fail:

1. Titanium allergy: A very small percentage of people may have an allergy to titanium, which can cause the body to reject the implant.
2. Infection: If the implant site becomes infected, it can lead to implant failure.
3. Poor osseointegration: This is when the implant does not properly fuse with the jawbone, causing it to become loose.
4. Gum recession: If the gums recede around the implant, it can expose the implant and lead to failure.
5. Mechanical issues: Problems with the implant itself, such as improper placement or damage, can also cause failure.

Early symptoms of potential rejection or failure include persistent pain, swelling, inflammation, and bleeding around the implant site. If you experience any of these symptoms, it is important to contact your dentist promptly.
Do you have any specific concerns about dental implants?

11: How long do dental implants typically last?

A. Dental implants are designed to be a long-term solution for missing teeth. On average, the implant itself, which is the titanium post embedded in the jawbone, can last 15 to 25 years or even longer with proper care[1]. In some cases, they can last a lifetime.[1] However, the crown, bridge, or denture attached to the implant may need to be replaced every 10 to 15 years due to normal wear and tear. Factors such as good oral hygiene, regular dental check-ups, and avoiding habits like smoking can significantly impact the longevity of dental implants.
Do you have any specific concerns about dental implants?
B. Dental implants are designed to be a long-term solution for missing teeth. On average, the implant itself, which is the titanium post embedded in the jawbone, can last 15 to 25 years or even longer with proper care[1]. In some cases, they can last a lifetime. However, the crown, bridge, or denture attached to the implant may need to be replaced every 10 to 15 years due to normal wear and tear. Factors such as good oral hygiene, regular dental check-ups, and avoiding habits like smoking can significantly impact the longevity of dental implants.
Do you have any specific concerns about dental implants?
C. Dental implants are designed to be a long-term solution for missing teeth. On average, the implant itself, which is the titanium post embedded in the jawbone, can last 15 to 25 years or even longer with proper care. In some cases, they can last a lifetime. <u>However, the crown, bridge, or denture attached to the implant may need to be replaced every 10 to 15 years due to normal wear and tear</u>. <u>Factors such as good oral hygiene, regular dental check-ups, and avoiding habits like smoking can significantly impact the longevity of dental implants</u>.
Do you have any specific concerns about dental implants?

12: What are the potential risks associated with dental implants?

A. Dental implants are a reliable solution for replacing missing teeth, but they do come with some potential risks. Here are a few to be aware of:

1. Infection: This can occur at the implant site and may require treatment with antibiotics or, in severe cases, removal of the implant.
2. Nerve Damage: If the implant is placed too close to a nerve, it can cause numbness, tingling, or pain in the lips, gums, or chin.
3. Sinus Issues: For implants in the upper jaw, there's a risk of them protruding into the sinus cavities, which can lead to sinus infections.
4. Implant failure: Sometimes, the bone doesn't integrate properly with the implant, leading to a loose implant that may need to be removed.
5. Gum recession: The gum tissue around the implant can recede, exposing the metal post and causing inflammation.
6. Bone loss: Over time, the bone around the implant can deteriorate, compromising the stability of the implant.

It's important to discuss these risks with your dentist to ensure you're a good candidate for the procedure and to understand how to minimize these risks. Do you have any specific concerns or questions about dental implants?
B. Dental implants are a reliable solution for replacing missing teeth, but they do come with some potential risks. Here are a few to be aware of:

1. Infection: This can occur at the implant site and may require treatment with antibiotics or, in severe cases, removal of the implant.
2. Nerve damage: If the implant is placed too close to a nerve, it can cause numbness, tingling, or pain in the lips, gums, or chin.
3. Sinus issues: For implants in the upper jaw, there's a risk of them protruding into the sinus cavities, which can lead to sinus infections.

(*Continued*)

 © 2025. The Author(s).

Supplementary Table S1 (*Continued*)

4. Implant failure: Sometimes, the bone doesn't integrate properly with the implant, leading to a loose implant that may need to be removed.
5. Gum recession: The gum tissue around the implant can recede, exposing the metal post and causing inflammation.
6. Bone loss: Over time, the bone around the implant can deteriorate, compromising the stability of the implant.

It's important to discuss these risks with your dentist to ensure you're a good candidate for the procedure and to understand how to minimize these risks. Do you have any specific concerns or questions about dental implants?

C. Dental implants are a reliable solution for replacing missing teeth, but they do come with some potential risks. Here are a few to be aware of:

1. Infection: This can occur at the implant site and may require treatment with antibiotics or, in severe cases, removal of the implant.
2. Nerve damage: If the implant is placed too close to a nerve, it can cause numbness, tingling, or pain in the lips, gums, or chin.
3. Sinus issues: For implants in the upper jaw, there's a risk of them protruding into the sinus cavities, which can lead to sinus infections.
4. Implant failure: Sometimes, the bone doesn't integrate properly with the implant, leading to a loose implant that may need to be removed.
5. Gum recession: The gum tissue around the implant can recede, exposing the metal post and causing inflammation.
6. Bone loss: Over time, the bone around the implant can deteriorate, compromising the stability of the implant.

It's important to discuss these risks with your dentist to ensure you're a good candidate for the procedure and to understand how to minimize these risks. Do you have any specific concerns or questions about dental implants?

13. Can I get dental implant during pregnancy?

A. Getting dental implants during pregnancy is generally not recommended unless it's an urgent medical necessity. Here are a few reasons why:

1. Radiation exposure: Dental implants often require X-rays, which involve a small amount of radiation. Although dental X-rays are considered safe during pregnancy, many prefer to avoid any radiation exposure to the unborn child
2. Anesthesia concerns while local anesthesia is safe during pregnancy dental implants surgery might require stronger anesthetic which could pose risks to both the mother and the baby.[REMOVED HYPERLINK FIELD]
3. Anxiety pregnancy can already be a stressful time and undergoing elective surgery might add unnecessary anxiety which can affect both the mother and the baby.
4. Timing: Most dentist and oral surgeon recommend waiting until after pregnancy to perform elective procedure like dental implant Thank you

However, maintaining good oral health during pregnancy is crucial. Regular checkups, cleanings, and non-invasive treatments are safe and recommended. If you have any specific concerns or urgent dental issues, it's best to consult with your dentist and obstetrician to determine the safest course of action for you and your baby.

If you have any more questions or need further assistance, feel free to ask!

B.

Getting dental implants during pregnancy is generally not recommended unless it's an urgent medical necessity. Here are a few reasons why:

1. **Radiation exposure**: Dental implants often require X-rays, which involve a small amount of radiation. Although dental X-rays are considered safe during pregnancy, many prefer to avoid any radiation exposure to the unborn child
2. **Anesthesia concerns**: While local anesthesia is safe during pregnancy, dental implant surgery might require stronger anesthetics, which could pose risks to both the mother and the baby
3. **Stress and anxiety**: Pregnancy can already be a stressful time, and undergoing elective surgery might add unnecessary anxiety, which can affect both the mother and the baby.[1]
4. **Timing**: Most dentists and oral surgeons recommend waiting until after pregnancy to perform elective procedures like dental implants

However, maintaining good oral health during pregnancy is crucial. Regular checkups, cleanings, and non-invasive treatments are safe and recommended. If you have any specific concerns or urgent dental issues, it's best to consult with your dentist and obstetrician to determine the safest course of action for you and your baby.

If you have any more questions or need further assistance, feel free to ask!

C. Getting dental implants during pregnancy is generally not recommended unless it's an urgent medical necessity. Here are a few reasons why:

1. **Radiation exposure**: Dental implants often require X-rays, which involve a small amount of radiation. Although dental X-rays are considered safe during pregnancy, many prefer to avoid any radiation exposure to the unborn child.
2. **Anesthesia concerns**: While local anesthesia is safe during pregnancy, dental implant surgery might require stronger anesthetics, which could pose risks to both the mother and the baby.
3. **Stress and anxiety**: Pregnancy can already be a stressful time, and undergoing elective surgery might add unnecessary anxiety, which can affect both the mother and the baby.[1]
4. **Timing**: Most dentists and oral surgeons recommend waiting until after pregnancy to perform elective procedures like dental implants.

However, maintaining good oral health during pregnancy is crucial. Regular checkups, cleanings, and non-invasive treatments are safe and recommended. If you have any specific concerns or urgent dental issues, it's best to consult with your dentist and obstetrician to determine the safest course of action for you and your baby.

If you have any more questions or need further assistance, feel free to ask!

Supplementary Table S1 (*Continued*)

14: What is peri-implantitis?

A. Peri-implantitis is a pathological condition that affects the tissues surrounding dental implants. It is characterized by inflammation in the peri-implant mucosa (the soft tissue around the implant) and progressive loss of the supporting bone.

Key features of peri-implantitis:

- Inflammation: The tissues around the implant become red, swollen, and may bleed when probed.
- Bone loss: There is a progressive loss of the bone supporting the implant, which can be seen on radiographs.
- Symptoms: Patients might experience pain, swelling, and sometimes pus discharge around the implant. In severe cases, the implant may become loose.

Treatment:

Managing peri-implantitis can be challenging and often requires a combination of non-surgical and surgical approaches. The primary goals are to reduce inflammation and prevent further bone loss.

If you suspect you have peri-implantitis, it's important to consult with your dentist or periodontist for an accurate diagnosis and appropriate treatment plan.

Do you have any other questions about dental health?

B. Peri-implantitis is a destructive inflammatory condition that affects the tissues surrounding dental implants. It involves both the soft tissues (gums) and the hard tissues (bone) around the implant.

Key characteristics:

- Inflammation: The gums around the implant become red, swollen, and may bleed when probed.
- Bone loss: There is a progressive loss of the bone supporting the implant, which can be seen on X-rays.
- Symptoms: Patients might experience pain, swelling, and sometimes pus discharge around the implant. In severe cases, the implant may become loose.

Treatment:

Managing peri-implantitis can be challenging and often requires a combination of non-surgical and surgical approaches. The primary goals are to reduce inflammation and prevent further bone loss

If you suspect you have peri-implantitis, it's important to consult with your dentist or periodontist for an accurate diagnosis and appropriate treatment plan.

Do you have any other questions about dental health?

C. Peri-implantitis is a serious inflammatory condition that affects the tissues surrounding dental implants. It involves both the soft tissues (gums) and the hard tissues (bone) around the implant

Key characteristics:

- Inflammation: The gums around the implant become red, swollen, and may bleed when probed.
- Bone loss: There is a progressive loss of the bone supporting the implant, which can be seen on X-rays.
- Symptoms: Patients might experience pain, swelling, and sometimes pus discharge around the implant. In severe cases, the implant may become loose.

Treatment:

Managing peri-implantitis can be challenging and often requires a combination of non-surgical and surgical approaches. The primary goals are to reduce inflammation and prevent further bone loss

If you suspect you have peri-implantitis, it's important to consult with your dentist or periodontist for an accurate diagnosis and appropriate treatment plan.

Do you have any other questions about dental health?

15: How many dental implants do I need if I want to restore my edentulous upper and lower Jaw?

a. Restoring an edentulous (toothless) upper and lower jaw typically involves several dental implants. The exact number can vary based on the specific treatment plan and your oral health. Here are some common approaches:

1. Implant-supported dentures: This option usually requires 4 to 6 implants per jaw. These implants support a removable denture that snaps onto the implants, providing more stability than traditional dentures
2. Fixed implant bridges: For a more permanent solution, 6 to 8 implants per jaw are often used to support a fixed bridge. This option provides a stable and non-removable set of teeth
3. All-on-4 treatment concept: This is a popular method where 4 implants per jaw are strategically placed to support a full arch of teeth. This approach can often be done in a single day

The best option for you depends on various factors, including bone quality, overall health, and personal preferences. It's essential to consult with a dental specialist to determine the most suitable treatment plan for your needs.

Would you like more detailed information on any of these options?

b. Restoring an edentulous (toothless) upper and lower jaw typically involves several dental implants. The exact number can vary based on the specific treatment plan and your oral health. Here are some common approaches:

1. Implant-supported dentures: This option usually requires 4 to 6 implants per jaw. These implants support a removable denture that snaps onto the implants, providing more stability than traditional dentures
2. Fixed implant bridges: For a more permanent solution, 6 to 8 implants per jaw are often used to support a fixed bridge. This option provides a stable and non-removable set of teeth
3. **All-on-4 treatment concept**: This is a popular method where **4 implants per jaw** are strategically placed to support a full arch of teeth. <u>This approach can often be done in a single day</u>

The best option for you depends on various factors, including bone quality, overall health, and personal preferences. It's essential to consult with a dental specialist to determine the most suitable treatment plan for your needs.

Would you like more detailed information on any of these options?

c. Restoring an edentulous (toothless) upper and lower jaw typically involves several dental implants. The exact number can vary based on the specific treatment plan and your oral health. Here are some common approaches:

(*Continued*)

 © 2025. The Author(s).

Supplementary Table S1 (*Continued*)

1. **Implant-supported dentures**: This option usually requires **4 to 6 implants per jaw**. These implants support a removable denture that snaps onto the implants, providing more stability than traditional dentures
2. **Fixed implant bridges**: For a more permanent solution, **6 to 8 implants per jaw** are often used to support a fixed bridge. This option provides a stable and non-removable set of teeth
3. **All-on-4 treatment concept**: This is a popular method where **4 implants per jaw** are strategically placed to support a full arch of teeth. This approach can often be done in a single day

The best option for you depends on various factors, including bone quality, overall health, and personal preferences. It's essential to consult with a dental specialist to determine the most suitable treatment plan for your needs.
Would you like more detailed information on any of these options?

16. Do I need to replace my dental implants in the future?

Dental implants are designed to be a long-lasting solution for missing teeth, and with proper care, they can last many years, often decades. However, there are some situations where you might need to replace them:

1. **Implant Failure**: Although rare, implants can fail due to infection, insufficient bone support, or improper placement
2. **Wear and Tear**: The crown (the visible part of the implant) may wear down over time and need replacement, even if the implant itself remains intact
3. **Bone Loss**: Over time, bone loss around the implant can occur, which might necessitate replacement or additional procedures like bone grafting

Regular dental check-ups and good oral hygiene are crucial to maintaining the longevity of your dental implants. If you experience any issues or discomfort, it's best to consult with your dentist promptly.
How long have you had your dental implants?

B. Dental implants are designed to be a long-lasting solution for missing teeth, and with proper care, they can last many years, often decades. However, there are some situations where you might need to replace them:

1. **Implant Failure**: Although rare, implants can fail due to infection, insufficient bone support, or improper placement.
2. **Wear and Tear**: The crown (the visible part of the implant) may wear down over time and need replacement, even if the implant itself remains intact.
3. **Bone Loss**: Over time, bone loss around the implant can occur, which might necessitate replacement or additional procedures like bone grafting.

Regular dental check-ups and good oral hygiene are crucial to maintaining the longevity of your dental implants. If you experience any issues or discomfort, it's best to consult with your dentist promptly.
How long have you had your dental implants?

C. Dental implants are designed to be a long-lasting solution for missing teeth, and with proper care, they can last many years, often decades. However, there are some situations where you might need to replace them:

1. Implant Failure: Although rare, implants can fail due to infection, insufficient bone support, or improper placement
2. Wear and Tear: The crown (the visible part of the implant) may wear down over time and need replacement, even if the implant itself remains intact
3. Bone Loss: Over time, bone loss around the implant can occur, which might necessitate replacement or additional procedures like bone grafting

Regular dental check-ups and good oral hygiene are crucial to maintaining the longevity of your dental implants. If you experience any issues or discomfort, it's best to consult with your dentist promptly.
How long have you had your dental implants?

17. Can I replace my complete denture with implant supported dental prothesis?

A. Yes, you can replace your complete denture with an implant-supported dental prosthesis. This option is often recommended for several reasons:

1. Stability and Comfort: Implant-supported dentures are more stable and comfortable compared to traditional dentures. They are anchored to dental implants, which are surgically placed into your jawbone, providing a secure fit that prevents slipping and irritation
2. Improved Functionality: With implant-supported dentures, you can chew and speak more effectively. They distribute the biting force more evenly across your jaw, which can help preserve your jawbone and prevent bone loss
3. Aesthetic Benefits: These dentures look and feel more like natural teeth. They can help maintain the structure of your face and prevent the sunken appearance that can occur with traditional dentures
4. Long-Term Solution: Implant-supported dentures are a long-term solution. With proper care, they can last many years, making them a worthwhile investment

However, it's important to consult with your dentist to determine if you are a good candidate for this procedure. Factors such as bone density, overall health, and oral hygiene habits will be considered to ensure the best outcome
Would you like more detailed information on the procedure or the types of implant-supported dentures available?
B.
Yes, you can replace your complete denture with an implant-supported dental prosthesis. This option is often recommended for several reasons:

1. **Stability and Comfort**: Implant-supported dentures are more stable and comfortable compared to traditional dentures. They are anchored to dental implants, which are surgically placed into your jawbone, providing a secure fit that prevents slipping and irritation
2. **Improved Functionality**: With implant-supported dentures, you can chew and speak more effectively. They distribute the biting force more evenly across your jaw, which can help preserve your jawbone and prevent bone loss
3. **Aesthetic Benefits**: These dentures look and feel more like natural teeth. They can help maintain the structure of your face and prevent the sunken appearance that can occur with traditional dentures

 © 2025. The Author(s).

Supplementary Table S1 (*Continued*)

4. **Long-Term Solution**: Implant-supported dentures are a long-term solution. With proper care, they can last many years, making them a worthwhile investment

However, it's important to consult with your dentist to determine if you are a good candidate for this procedure. Factors such as bone density, overall health, and oral hygiene habits will be considered to ensure the best outcome
Would you like more detailed information on the procedure or the types of implant-supported dentures available?
C. Yes, you can replace your complete denture with an implant-supported dental prosthesis. This option is often recommended for several reasons:

1. Stability and Comfort: Implant-supported dentures are more stable and comfortable compared to traditional dentures. They are anchored to dental implants, which are surgically placed into your jawbone, providing a secure fit that prevents slipping and irritation
2. Improved Functionality: With implant-supported dentures, you can chew and speak more effectively. They distribute the biting force more evenly across your jaw, which can help preserve your jawbone and prevent bone loss
3. Aesthetic Benefits: These dentures look and feel more like natural teeth. They can help maintain the structure of your face and prevent the sunken appearance that can occur with traditional dentures
4. Long-Term Solution: Implant-supported dentures are a long-term solution. With proper care, they can last many years, making them a worthwhile investment

However, it's important to consult with your dentist to determine if you are a good candidate for this procedure. Factors such as bone density, overall health, and oral hygiene habits will be considered to ensure the best outcome.
Would you like more detailed information on the procedure or the types of implant-supported dentures available?
18. How should I care of my dental implant?
A. Caring for your dental implant is crucial to ensure it heals properly and lasts a long time. Here are some key tips for both immediate and long-term care:
Immediate Aftercare

1. Rest and Recovery: After the procedure, rest until the effects of the anesthesia wear off. Avoid strenuous activities for a few days.
2. Manage Bleeding: Light bleeding is normal. Bite gently on a damp gauze to help control it.
3. Soft Foods: Stick to soft foods for at least a week to avoid putting pressure on the implant.
4. Oral Hygiene: Keep your mouth clean but avoid brushing the implant site for the first few days. Use a recommended mouthwash or warm salt water rinses.
5. Pain Management: Take prescribed antibiotics and over-the-counter pain relief like paracetamol or ibuprofen if needed.

Long-Term Care

1. Maintain Oral Hygiene: Brush and floss regularly, but be gentle around the implant area.
2. Regular Check-ups: Visit your dentist regularly to monitor the implant and overall oral health.
3. Avoid Harmful Habits: Refrain from smoking and excessive alcohol consumption as they can hinder the healing process.
4. Healthy Diet: Eat a balanced diet to support overall health and healing.

If you experience any unusual symptoms like persistent pain, swelling, or bleeding, contact your dentist immediately.
Do you have any specific concerns or questions about your dental implant?
B. Caring for your dental implant properly is essential for its longevity and your overall oral health. Here are some key tips for both immediate and long-term care:
Immediate Aftercare

1. Rest and Recovery: After the procedure, rest until the effects of the anesthesia wear off. Avoid strenuous activities for a few days
2. Manage Bleeding: Light bleeding is normal. Apply gentle pressure with a damp gauze if needed
3. Swelling and Bruising: Use an ice pack on the affected area for 10 minutes every hour to reduce swelling
4. Diet: Stick to soft foods for at least a week. Avoid hot foods and drinks for the first 24 hours

Long-term Care

1. Oral Hygiene: Brush and floss daily, just like you would with natural teeth. Use a soft-bristled toothbrush and non-abrasive toothpaste.
2. Regular Check-ups: Visit your dentist regularly for cleanings and check-ups to monitor the implant.
3. Avoid Hard Foods: Refrain from chewing hard items like ice or hard candies that could damage the implant.
4. Lifestyle Choices: Limit caffeine and avoid tobacco products as they can stain your teeth and affect the implant.

Additional Tips

- Mouthwash: Use a mouthwash recommended by your dentist to keep the area clean.
- Medication: Take any prescribed antibiotics and pain relief as directed.

Following these guidelines will help ensure your dental implant remains healthy and functional. If you experience any unusual symptoms or have concerns, don't hesitate to contact your dentist. How are you feeling about your implant so far?
C. Caring for your dental implant properly is essential for its longevity and your overall oral health. Here are some key tips for both immediate and long-term care:
Immediate Aftercare

1. Rest and Recovery: After the procedure, rest until the effects of the anesthesia wear off. Avoid strenuous activities for a few days.
2. Manage Bleeding: Light bleeding is normal. Apply gentle pressure with a damp gauze if needed.
3. Swelling and Bruising: Use an ice pack on the affected area for 10 minutes every hour to reduce swelling
4. Diet: Stick to soft foods for at least a week. Avoid hot foods and drinks for the first 24 hours

(*Continued*)

 © 2025. The Author(s).

Supplementary Table S1 (*Continued*)

Long-term Care
1. Oral Hygiene: Brush and floss daily, just like you would with natural teeth. Use a soft-bristled toothbrush and non-abrasive toothpaste.
2. Regular Check-ups: Visit your dentist regularly for cleanings and check-ups to monitor the implant.
3. Avoid Hard Foods: Refrain from chewing hard items like ice or hard candies that could damage the implant.
4. Lifestyle Choices: Limit caffeine and avoid tobacco products as they can stain your teeth and affect the implant.

Additional Tips
- Mouthwash: Use a mouthwash recommended by your dentist to keep the area clean.
- Medication: Take any prescribed antibiotics and pain relief as directed.

Following these guidelines will help ensure your dental implant remains healthy and functional. If you experience any unusual symptoms or have concerns, don't hesitate to contact your dentist. How are you feeling about your implant so far?

19. How long does the entire dental implant process take?

A. The entire dental implant process typically takes several months and involves multiple stages. Here's a general timeline:
1. Initial Consultation: This is where your dentist evaluates your oral health and determines if you're a suitable candidate for implants. This can take a few days to a couple of weeks, depending on scheduling.
2. Implant Placement: The actual placement of the implant into your jawbone is usually done under local anesthesia and can take about 1-2 hours per implant.
3. Healing and Osseointegration: After the implant is placed, it needs time to fuse with the jawbone in a process called osseointegration. This can take anywhere from 3 to 6 months
4. Abutment Placement: Once the implant has integrated with the bone, an abutment is placed on top of the implant. This minor procedure usually takes about 1-2 weeks to heal
5. Crown Placement: Finally, a custom-made crown is attached to the abutment. This step can take a few weeks, as the crown needs to be fabricated and adjusted for a perfect fit.[2]

Overall, the entire process can take between 4 to 9 months, but it can vary depending on individual circumstances and any additional procedures needed, such as bone grafts
Is there a specific part of the process you're curious about?

B. The entire dental implant process typically takes several months and involves multiple stages. Here's a general timeline:
1. Initial Consultation: This is where your dentist evaluates your oral health and determines if you're a suitable candidate for implants. This can take a few days to a couple of weeks, depending on scheduling
2. Implant Placement: The actual placement of the implant into your jawbone is usually done under local anesthesia and can take about 1-2 hours per implant
3. Healing and Osseointegration: After the implant is placed, it needs time to fuse with the jawbone in a process called osseointegration. This can take anywhere from 3 to 6 months
4. Abutment Placement: Once the implant has integrated with the bone, an abutment is placed on top of the implant. This minor procedure usually takes about 1-2 weeks to heal
5. Crown Placement: Finally, a custom-made crown is attached to the abutment. This step can take a few weeks, as the crown needs to be fabricated and adjusted for a perfect fit

Overall, the entire process can take between 4 to 9 months, but it can vary depending on individual circumstances and any additional procedures needed, such as bone grafts
Is there a specific part of the process you're curious about?

C. The entire dental implant process typically takes several months and involves multiple stages. Here's a general timeline:
1. Initial Consultation: This is where your dentist evaluates your oral health and determines if you're a suitable candidate for implants. This can take a few days to a couple of weeks, depending on scheduling
2. Implant Placement: The actual placement of the implant into your jawbone is usually done under local anesthesia and can take about 1-2 hours per implant
3. Healing and Osseointegration: After the implant is placed, it needs time to fuse with the jawbone in a process called osseointegration. This can take anywhere from 3 to 6 months
4. Abutment Placement: Once the implant has integrated with the bone, an abutment is placed on top of the implant. This minor procedure usually takes about 1-2 weeks to heal
5. Crown Placement: Finally, a custom-made crown is attached to the abutment. This step can take a few weeks, as the crown needs to be fabricated and adjusted for a perfect fit.

Overall, the entire process can take between 4 to 9 months, but it can vary depending on individual circumstances and any additional procedures needed, such as bone grafts.
Is there a specific part of the process you're curious about?

20. Is It Possible to Get "Metal-Free" Implants

A. Yes, it is possible to get "metal-free" implants! These implants are typically made from materials like ceramic or zirconia, which are biocompatible and do not contain any metallic elements

Advantages of Metal-Free Implants:
- Aesthetic Appeal: They blend seamlessly with natural teeth, providing a more natural look
- Biocompatibility: They are less likely to cause allergic reactions or sensitivities
- Durability: Despite being metal-free, materials like zirconia are strong and resistant to wear

Considerations:
- Cost: Metal-free implants can be more expensive than traditional metal implants
- Strength: They may not be as strong as titanium implants, which could affect their longevity

Supplementary Table S1 (*Continued*)

Would you like to know more about the process or where you can get these implants?
B. Yes, it is possible to get "metal-free" implants! These implants are typically made from materials like ceramic or zirconia, which are biocompatible and do not contain any metallic elements.
Advantages of Metal-Free Implants:
- Aesthetic Appeal: They blend seamlessly with natural teeth, providing a more natural look.
- Biocompatibility: They are less likely to cause allergic reactions or sensitivities.
- Durability: Despite being metal-free, materials like zirconia are strong and resistant to wear

Considerations:
- Cost: Metal-free implants can be more expensive than traditional metal implants.[1]
- Strength: They may not be as strong as titanium implants, which could affect their longevity.

Would you like to know more about the process or where you can get these implants?
C. Yes, it is possible to get "metal-free" implants! These implants are typically made from materials like ceramic or zirconia, which are biocompatible and do not contain any metallic elements.
Advantages of Metal-Free Implants:
- Aesthetic Appeal: They blend seamlessly with natural teeth, providing a more natural look.
- Biocompatibility: They are less likely to cause allergic reactions or sensitivities.
- Durability: Despite being metal-free, materials like zirconia are strong and resistant to wear.

Considerations:
- Cost: Metal-free implants can be more expensive than traditional metal implants.
- Strength: They may not be as strong as titanium implants, which could affect their longevity.

Would you like to know more about the process or where you can get these implants?

Google Bard/Gemini

Q1: what is dental implant?
A. A dental implant is a titanium post that is surgically placed into the jawbone to replace a missing tooth root. It acts as a support for an artificial tooth (crown), bridge, or denture.
Here's a breakdown of the process:
1. Implant Placement: The dentist places the implant into the jawbone where the tooth root used to be.
2. Osseointegration: Over several months, the implant fuses with the jawbone, creating a strong foundation.
3. Abutment Placement: Once the implant has healed, an abutment (a connector piece) is attached to it.
4. Crown Placement: The final step is to attach the artificial tooth (crown) to the abutment.

Dental implants offer several benefits, including:
- Improved appearance: They look and feel natural.
- Enhanced function: They allow you to chew and speak more comfortably.
- Durability: With proper care, dental implants can last a lifetime.
- Stability: Unlike dentures, dental implants don't slip or shift.

Would you like to know more about the dental implant procedure, its benefits, or the risks involved?
B. A dental implant is a titanium post that is surgically placed into the jawbone to replace a missing tooth root. It acts as a support for an artificial tooth (crown), bridge, or denture.
Here's a breakdown of the process:
1. Implant Placement: The dentist places the implant into the jawbone where the tooth root used to be.
2. Osseointegration: Over several months, the implant fuses with the jawbone, creating a strong foundation.
3. Abutment Placement: Once the implant has healed, an abutment (a connector piece) is attached to it.
4. Crown Placement: The final step is to attach the artificial tooth (crown) to the abutment.

Dental implants offer several benefits, including:
- Improved appearance: They look and feel natural.
- Enhanced function: They allow you to chew and speak more comfortably.
- Durability: With proper care, dental implants can last a lifetime.
- Stability: Unlike dentures, dental implants don't slip or shift.

Would you like to know more about the dental implant procedure, its benefits, or the risks involved?
C. A dental implant is a titanium post that is surgically placed into the jawbone to replace a missing tooth root. It acts as a support for an artificial tooth (crown), bridge, or denture.
Here's a breakdown of the process:
1. Implant Placement: The dentist places the implant into the jawbone where the tooth root used to be.
2. Osseointegration: Over several months, the implant fuses with the jawbone, creating a strong foundation.
3. Abutment Placement: Once the implant has healed, an abutment (a connector piece) is attached to it.
4. Crown Placement: The final step is to attach the artificial tooth (crown) to the abutment.

Dental implants offer several benefits, including:
- Improved appearance: They look and feel natural.
- Enhanced function: They allow you to chew and speak more comfortably.
- Durability: With proper care, dental implants can last a lifetime.
- Stability: Unlike dentures, dental implants don't slip or shift.

Would you like to know more about the dental implant procedure, its benefits, or the risks involved?
2. **Is everyone eligible to get a dental implant?**
A.Not everyone is eligible for dental implants. While they're a popular and effective option for replacing missing teeth, several factors can influence your candidacy:

(*Continued*)

 © 2025. The Author(s).

Supplementary Table S1 (*Continued*)

- Oral health: You need healthy gums and sufficient jawbone density to support the implant.
- Overall health: Certain medical conditions or medications can affect your eligibility.
- Smoking: Smoking can hinder healing and increase the risk of complications.
- Jawbone development: If you're under 18, your jawbone may not be fully developed.

It's essential to consult with a dentist to determine if dental implants are a suitable option for you. They can assess your individual circumstances and provide personalized advice.

B. Not everyone is eligible for dental implants. While they're a popular and effective option for replacing missing teeth, several factors can influence your candidacy:

- Oral health: You need healthy gums and sufficient jawbone density to support the implant.
- Overall health: Certain medical conditions or medications can affect your eligibility.
- Smoking: Smoking can hinder healing and increase the risk of complications.
- Jawbone development: If you're under 18, your jawbone may not be fully developed.

It's essential to consult with a dentist to determine if dental implants are a suitable option for you. They can assess your individual circumstances and provide personalized advice.

C. Not everyone is eligible for dental implants. While they're a popular and effective option for replacing missing teeth, several factors can influence your candidacy:

- Oral health: You need healthy gums and sufficient jawbone density to support the implant.
- Overall health: Certain medical conditions or medications can affect your eligibility.
- Smoking: Smoking can hinder healing and increase the risk of complications.
- Jawbone development: If you're under 18, your jawbone may not be fully developed.

It's essential to consult with a dentist to determine if dental implants are a suitable option for you. They can assess your individual circumstances and provide personalized advice.

Q3. As a diabetic patient, will a dental implant be successful for me?

A. Yes, diabetic patients can often be successful candidates for dental implants. However, the success rate can be influenced by how well your diabetes is managed.

Here are some factors to consider:

- Blood sugar control: Well-controlled blood sugar levels can significantly improve the success rate of dental implants. Regular monitoring and adherence to your treatment plan are essential.
- Oral health: Maintaining good oral hygiene is crucial for preventing complications. Brush twice daily, floss regularly, and see your dentist for regular check-ups.
- Gum health: Periodontal disease, a common complication of diabetes, can affect the success of dental implants. It's important to address any gum issues before implant placement.

It's essential to consult with your dentist and discuss your specific situation. They can assess your overall health and determine if dental implants are a suitable option for you.

B. Yes, diabetic patients can often be successful candidates for dental implants. However, the success rate can be influenced by how well your diabetes is managed.

Here are some factors to consider:

- Blood sugar control: Well-controlled blood sugar levels can significantly improve the success rate of dental implants. Regular monitoring and adherence to your treatment plan are essential.
- Oral health: Maintaining good oral hygiene is crucial for preventing complications. Brush twice daily, floss regularly, and see your dentist for regular check-ups.
- Gum health: Periodontal disease, a common complication of diabetes, can affect the success of dental implants. It's important to address any gum issues before implant placement.

It's essential to consult with your dentist and discuss your specific situation. They can assess your overall health and determine if dental implants are a suitable option for you.

C. Yes, diabetic patients can often be successful candidates for dental implants. However, the success rate can be influenced by how well your diabetes is managed.

Here are some factors to consider:

- Blood sugar control: Well-controlled blood sugar levels can significantly improve the success rate of dental implants. Regular monitoring and adherence to your treatment plan are essential.
- Oral health: Maintaining good oral hygiene is crucial for preventing complications. Brush twice daily, floss regularly, and see your dentist for regular check-ups.
- Gum health: Periodontal disease, a common complication of diabetes, can affect the success of dental implants. It's important to address any gum issues before implant placement.

It's essential to consult with your dentist and discuss your specific situation. They can assess your overall health and determine if dental implants are a suitable option for you.

Q4: **What impact does smoking have on dental implants?**

A. Smoking significantly impacts the success of dental implants. Here's why:

- Delayed healing: Nicotine reduces blood flow, hindering the healing process and making it difficult for the implant to fuse with the bone.
- Increased risk of infection: Smoking weakens the immune system, making it more susceptible to infections around the implant site.
- Higher risk of implant failure: Due to delayed healing and increased infections, the chances of the implant failing are significantly higher in smokers.
- Gum disease: Smoking is a major risk factor for gum disease, which can lead to bone loss and further jeopardize the success of dental implants.

 © 2025. The Author(s).

Supplementary Table S1 (*Continued*)

If you're considering dental implants, it's strongly recommended that you quit smoking before the procedure. Even after the procedure, continuing to smoke can increase the risk of complications and shorten the lifespan of your implants.<br>If you're struggling to quit, consider seeking professional help from a smoking cessation program or therapist.<br>B. Smoking significantly impacts the success of dental implants. Here's why:<br>• Delayed healing: Nicotine reduces blood flow, hindering the healing process and making it difficult for the implant to fuse with the bone.<br>• Increased risk of infection: Smoking weakens the immune system, making it more susceptible to infections around the implant site.<br>• Higher risk of implant failure: Due to delayed healing and increased infections, the chances of the implant failing are significantly higher in smokers.<br>• Gum disease: Smoking is a major risk factor for gum disease, which can lead to bone loss and further jeopardize the success of dental implants.<br>If you're considering dental implants, it's strongly recommended that you quit smoking before the procedure. Even after the procedure, continuing to smoke can increase the risk of complications and shorten the lifespan of your implants.<br>If you're struggling to quit, consider seeking professional help from a smoking cessation program or therapist.<br>C. Smoking significantly impacts the success of dental implants. Here's why:<br>• Delayed healing: Nicotine reduces blood flow, hindering the healing process and making it difficult for the implant to fuse with the bone.<br>• Increased risk of infection: Smoking weakens the immune system, making it more susceptible to infections around the implant site.<br>• Higher risk of implant failure: Due to delayed healing and increased infections, the chances of the implant failing are significantly higher in smokers.<br>• Gum disease: Smoking is a major risk factor for gum disease, which can lead to bone loss and further jeopardize the success of dental implants.<br>If you're considering dental implants, it's strongly recommended that you quit smoking before the procedure. Even after the procedure, continuing to smoke can increase the risk of complications and shorten the lifespan of your implants.<br>If you're struggling to quit, consider seeking professional help from a smoking cessation program or therapist.<br>Q5. **How soon after a tooth extraction can an implant be placed?**<br>A.The timing of implant placement after a tooth extraction can vary depending on several factors, including:<br>• Jawbone density: If your jawbone is strong enough, an implant can sometimes be placed immediately after the extraction.<br>• Gum health: Healthy gums are crucial for successful implant placement.<br>• Type of extraction: The complexity of the extraction can influence healing time.<br>• Individual healing process: Everyone heals at a different pace.<br>Generally, there are three main timing options:<br>1. Immediate implant placement: This involves placing the implant at the same time as the extraction. It's often possible when the extraction is straightforward and the jawbone is healthy.<br>2. Early implant placement: This happens a few weeks or months after the extraction, once the initial healing has occurred.<br>3. Delayed implant placement: This involves waiting several months or even a year for the extraction site to fully heal before placing the implant.<br>It's important to consult with your dentist to determine the best timing for your specific situation. They will assess your oral health and recommend the most appropriate approach.<br>B. The timing of implant placement after a tooth extraction can vary depending on several factors, including:<br>• Jawbone density: If your jawbone is strong enough, an implant can sometimes be placed immediately after the extraction.<br>• Gum health: Healthy gums are crucial for successful implant placement.<br>• Type of extraction: The complexity of the extraction can influence healing time.<br>• Individual healing process: Everyone heals at a different pace.<br>Generally, there are three main timing options:<br>1. Immediate implant placement: This involves placing the implant at the same time as the extraction. It's often possible when the extraction is straightforward and the jawbone is healthy.<br>2. Early implant placement: This happens a few weeks or months after the extraction, once the initial healing has occurred.<br>3. Delayed implant placement: This involves waiting several months or even a year for the extraction site to fully heal before placing the implant.<br>It's important to consult with your dentist to determine the best timing for your specific situation. They will assess your oral health and recommend the most appropriate approach.<br>C.The timing of implant placement after a tooth extraction can vary depending on several factors, including:<br>• Jawbone density: If your jawbone is strong enough, an implant can sometimes be placed immediately after the extraction.<br>• Gum health: Healthy gums are crucial for successful implant placement.<br>• Type of extraction: The complexity of the extraction can influence healing time.<br>• Individual healing process: Everyone heals at a different pace.<br>Generally, there are three main timing options:<br>1. Immediate implant placement: This involves placing the implant at the same time as the extraction. It's often possible when the extraction is straightforward and the jawbone is healthy.<br>2. Early implant placement: This happens a few weeks or months after the extraction, once the initial healing has occurred.<br>3. Delayed implant placement: This involves waiting several months or even a year for the extraction site to fully heal before placing the implant.

(*Continued*)

Supplementary Table S1 (*Continued*)

It's important to consult with your dentist to determine the best timing for your specific situation. They will assess your oral health and recommend the most appropriate approach.<br>**Q6: Will the crown be attached right after the implant procedure?**<br>a. In most cases, the crown will not be attached right after the implant procedure.<br>Here's why:<br>• Osseointegration: The implant needs time to fuse with your jawbone. This process, called osseointegration, is crucial for the implant's long-term stability. It typically takes several months.<br>• Healing: After the implant surgery, your gums and surrounding tissues need time to heal. Attaching a crown too soon could disrupt this healing process.<br>It's important to follow your dentist's instructions carefully during this healing period. This includes maintaining good oral hygiene and avoiding certain foods or activities that could irritate the area.<br>If you have any specific questions about your dental implant procedure, please consult with your dentist. They can provide you with personalized information based on your individual situation.<br>B. In most cases, the crown will not be attached right after the implant procedure.<br>Here's why:<br>• Osseointegration: The implant needs time to fuse with your jawbone. This process, called osseointegration, is crucial for the implant's long-term stability. It typically takes several months.<br>• Healing: After the implant surgery, your gums and surrounding tissues need time to heal. Attaching a crown too soon could disrupt this healing process.<br>It's important to follow your dentist's instructions carefully during this healing period. This includes maintaining good oral hygiene and avoiding certain foods or activities that could irritate the area.<br>If you have any specific questions about your dental implant procedure, please consult with your dentist. They can .provide you with personalized information based on your individual situation.<br>C. In most cases, the crown will not be attached right after the implant procedure.<br>Here's why:<br>• Osseointegration: The implant needs time to fuse with your jawbone. This process, called osseointegration, is crucial for the implant's long-term stability. It typically takes several months.<br>• Healing: After the implant surgery, your gums and surrounding tissues need time to heal. Attaching a crown too soon could disrupt this healing process.<br>It's important to follow your dentist's instructions carefully during this healing period. This includes maintaining good oral hygiene and avoiding certain foods or activities that could irritate the area.<br>If you have any specific questions about your dental implant procedure, please consult with your dentist. They can provide you with personalized information based on your individual situation.<br>**Q7: How is the recovery after dental implant implacment procedure?**<br>A. Recovery After Dental Implant Placement<br>Dental implant placement is a surgical procedure that requires a healing period. While the experience can vary from person to person, here's a general overview of what to expect:<br>Initial Weeks:<br>• Discomfort and Swelling: This is normal and can be managed with over-the-counter pain medication.<br>• Bleeding: Some mild bleeding is expected, but excessive bleeding should be reported to your dentist.<br>• Soft Food Diet: Eating soft foods will help protect the healing area.<br>• Oral Hygiene: Gentle brushing and flossing around the implant site are essential.<br>Months Later:<br>• Osseointegration: The implant will integrate with your jawbone, forming a strong foundation.<br>• Abutment Placement: A connector piece is placed on the implant.<br>• Crown Placement: The final restoration, which looks like a natural tooth, is attached to the abutment.<br>The entire healing process can take several months. It's important to follow your dentist's instructions carefully and attend regular check-ups.<br>Would you like to know more about the specific steps involved in dental implant placement or have any other questions?<br>B. Recovery After Dental Implant Placement<br>Dental implant placement is a surgical procedure that requires a healing period. While the experience can vary from person to person, here's a general overview of what to expect:<br>Initial Weeks:<br>• Discomfort and Swelling: This is normal and can be managed with over-the-counter pain medication.<br>• Bleeding: Some mild bleeding is expected, but excessive bleeding should be reported to your dentist.<br>• Soft Food Diet: Eating soft foods will help protect the healing area.<br>• Oral Hygiene: Gentle brushing and flossing around the implant site are essential.<br>Months Later:<br>• Osseointegration: The implant will integrate with your jawbone, forming a strong foundation.<br>• Abutment Placement: A connector piece is placed on the implant.<br>• Crown Placement: The final restoration, which looks like a natural tooth, is attached to the abutment.<br>The entire healing process can take several months. It's important to follow your dentist's instructions carefully and attend regular check-ups.

 © 2025. The Author(s).

Supplementary Table S1 (*Continued*)

Would you like to know more about the specific steps involved in dental implant placement or have any other questions?
C. Recovery After Dental Implant Placement
Dental implant placement is a surgical procedure that requires a healing period. While the experience can vary from person to person, here's a general overview of what to expect:
Initial Weeks:

- Discomfort and Swelling: This is normal and can be managed with over-the-counter pain medication.
- Bleeding: Some mild bleeding is expected, but excessive bleeding should be reported to your dentist.
- Soft Food Diet: Eating soft foods will help protect the healing area.
- Oral Hygiene: Gentle brushing and flossing around the implant site are essential.

Months Later:

- Osseointegration: The implant will integrate with your jawbone, forming a strong foundation.
- Abutment Placement: A connector piece is placed on the implant.
- Crown Placement: The final restoration, which looks like a natural tooth, is attached to the abutment.

The entire healing process can take several months. It's important to follow your dentist's instructions carefully and attend regular check-ups.
Would you like to know more about the specific steps involved in dental implant placement or have any other questions?
Q8: Which is better option to restore a missing tooth FPD or dental implant?
A. The choice between a Fixed Partial Denture (FPD) and a dental implant for restoring a missing tooth depends on several factors, including:
Factors favoring FPD:

- Cost: FPDs are generally less expensive than implants.
- Quick procedure: FPDs can be completed in fewer appointments than implants.
- No surgery: FPDs do not require invasive surgery.

Factors favoring dental implant:

- Long-term durability: Implants are designed to last for many years or even decades.
- Natural feel: Implants function and look like natural teeth.
- Preservation of adjacent teeth: FPDs require the preparation of adjacent teeth, while implants do not.
- Bone stimulation: Implants help to stimulate bone growth, which can prevent jawbone atrophy.

Ultimately, the best option for you will depend on your individual needs and preferences. It is important to consult with a qualified dentist to discuss the pros and cons of each option and determine which is the best fit for you.
Here are some additional factors to consider:

- Your oral health: If you have good oral health, you may be a good candidate for either FPD or implant. However, if you have gum disease or other oral health problems, you may need to address these issues before proceeding with either treatment.
- Your lifestyle: If you are an active person who participates in sports or other activities that put your teeth at risk, an implant may be a better option because it is more durable than an FPD.
- Your aesthetic goals: If you are concerned about the appearance of your teeth, an implant may be a better option because it can provide a more natural-looking result.

It is important to note that both FPDs and implants are effective ways to restore a missing tooth. However, it is important to choose the option that is right for you based on your individual needs and preferences.
B. The choice between a Fixed Partial Denture (FPD) and a dental implant for restoring a missing tooth depends on several factors, including:
FPD (Fixed Partial Denture)

- Pros: Less expensive, often quicker procedure, no surgery required.
- Cons: Requires preparing adjacent teeth, may not be as durable, doesn't stimulate bone growth.

Dental Implant

- Pros: Long-lasting, natural feel, preserves adjacent teeth, stimulates bone growth.
- Cons: More expensive, more invasive procedure, may require additional procedures like bone grafting.

Ultimately, the best option for you will depend on your individual circumstances and preferences.
Here are some factors to consider when making your decision:

- Cost: FPDs are generally less expensive than implants.
- Oral health: Your overall oral health, including bone density and gum health, can influence which option is suitable.
- Lifestyle: If you have an active lifestyle or bite down hard, implants may be more durable.
- Aesthetic goals: Implants often provide a more natural-looking result.

It's crucial to consult with a qualified dentist to discuss your specific needs and get their professional recommendation. They can evaluate your situation and provide personalized advice.
C. The choice between a Fixed Partial Denture (FPD) and a dental implant for restoring a missing tooth depends on several factors, including:
Factors favoring FPD:

- Cost: FPDs are generally less expensive than implants.
- Quick procedure: FPDs can be completed in fewer appointments than implants.
- No surgery: FPDs do not require invasive surgery.

Factors favoring dental implant:

- Long-term durability: Implants are designed to last for many years or even decades.
- Natural feel: Implants function and look like natural teeth.
- Preservation of adjacent teeth: FPDs require the preparation of adjacent teeth, while implants do not.

(*Continued*)

 © 2025. The Author(s).

Supplementary Table S1 (*Continued*)

- Bone stimulation: Implants help to stimulate bone growth, which can prevent jawbone atrophy.

Ultimately, the best option for you will depend on your individual needs and preferences. It is important to consult with a qualified dentist to discuss the pros and cons of each option and determine which is the best fit for you.
Here are some additional factors to consider:

- Your oral health: If you have good oral health, you may be a good candidate for either FPD or implant. However, if you have gum disease or other oral health problems, you may need to address these issues before proceeding with either treatment.
- Your lifestyle: If you are an active person who participates in sports or other activities that put your teeth at risk, an implant may be a better option because it is more durable than an FPD.
- Your aesthetic goals: If you are concerned about the appearance of your teeth, an implant may be a better option because it can provide a more natural-looking result.

It is important to note that both FPDs and implants are effective ways to restore a missing tooth. However, it is important to choose the option that is right for you based on your individual needs and preferences.
Q9: **Which is the better option: root canal treatment (RCT) or tooth extraction followed by an implant?**
A. The choice between root canal treatment (RCT) and tooth extraction followed by an implant depends on several factors, including:
RCT:

- Pros: Preserves the natural tooth, less invasive, often less expensive than an implant.
- **Cons:** May require multiple visits, may not be successful in all cases, can be painful.

Tooth Extraction and Implant:

- **Pros:** Provides a long-lasting, durable restoration, can improve aesthetics and function.
- **Cons:** More invasive, more expensive, requires more time for healing, may not be suitable for everyone (e.g., those with certain medical conditions or insufficient bone).

Ultimately, the best option for you will depend on your specific circumstances and preferences. It's important to consult with a qualified dentist who can assess your situation and recommend the most appropriate treatment.
Here are some additional factors to consider:

- Cost: Implants are generally more expensive than RCTs.
- Time: Implants require more time for healing and placement than RCTs.
- Risk: Implants have a higher success rate than RCTs, but they also involve more risk.
- Appearance: Implants are generally considered to be more natural-looking than RCTs.
- Comfort: Implants are generally more comfortable than RCTs.

By carefully considering these factors, you can make an informed decision about the best way to treat your tooth
B. The choice between root canal treatment (RCT) and tooth extraction followed by an implant depends on several factors, including:
RCT:

- Pros: Preserves the natural tooth, less invasive, often less expensive than an implant.
- Cons: May require multiple visits, may not be successful in all cases, can be painful.

Tooth Extraction and Implant:

- Pros: Provides a long-lasting, durable restoration, can improve aesthetics and function.
- Cons: More invasive, more expensive, requires more time for healing, may not be suitable for everyone (e.g., those with certain medical conditions or insufficient bone).

Ultimately, the best option for you will depend on your specific circumstances and preferences. It's important to consult with a qualified dentist who can assess your situation and recommend the most appropriate treatment.
Here are some additional factors to consider:

- Cost: Implants are generally more expensive than RCTs.
- Time: Implants require more time for healing and placement than RCTs.
- Risk: Implants have a higher success rate than RCTs, but they also involve more risk.
- Appearance: Implants are generally considered to be more natural-looking than RCTs.
- Comfort: Implants are generally more comfortable than RCTs.

By carefully considering these factors, you can make an informed decision about the best way to treat your tooth
C. The choice between root canal treatment (RCT) and tooth extraction followed by an implant depends on several factors, including:
RCT:

- Pros: Preserves the natural tooth, less invasive, often less expensive than an implant.
- Cons: May require multiple visits, may not be successful in all cases, can be painful.

Tooth Extraction and Implant:

- Pros: Provides a long-lasting, durable restoration, can improve aesthetics and function.
- Cons: More invasive, more expensive, requires more time for healing, may not be suitable for everyone (e.g., those with certain medical conditions or insufficient bone).

Ultimately, the best option for you will depend on your specific circumstances and preferences. It's important to consult with a qualified dentist who can assess your situation and recommend the most appropriate treatment.
Here are some additional factors to consider:

- Cost: Implants are generally more expensive than RCTs.
- Time: Implants require more time for healing and placement than RCTs.
- Risk: Implants have a higher success rate than RCTs, but they also involve more risk.
- Appearance: Implants are generally considered to be more natural-looking than RCTs.

Supplementary Table S1 (*Continued*)

- Comfort: Implants are generally more comfortable than RCTs.

By carefully considering these factors, you can make an informed decision about the best way to treat your tooth

Q10. Can the body reject a dental implant?

A.Yes, the body can reject a dental implant. However, this is a relatively rare occurrence. Dental implants are typically made of titanium, a biocompatible material that is less likely to be rejected by the body compared to other materials.

There are a few reasons why a body might reject a dental implant:

- Infection: Bacteria can infect the area around the implant, leading to inflammation and potential rejection.
- Poor oral hygiene: Not taking proper care of your oral health can increase the risk of infection and other complications.
- Jawbone issues: If your jawbone is not strong enough or healthy enough to support the implant, it may fail.

If you notice any signs of dental implant rejection, such as pain, swelling, or loose teeth, it's important to see your dentist right away. They can diagnose the problem and recommend appropriate treatment.

B. Yes, the body can reject a dental implant. However, this is a relatively rare occurrence. Dental implants are typically made of titanium, a biocompatible material that is less likely to be rejected by the body compared to other materials.

There are a few reasons why a body might reject a dental implant:

- Infection: Bacteria can infect the area around the implant, leading to inflammation and potential rejection.
- Poor oral hygiene: Not taking proper care of your oral health can increase the risk of infection and other complications.
- Jawbone issues: If your jawbone is not strong enough or healthy enough to support the implant, it may fail.

If you notice any signs of dental implant rejection, such as pain, swelling, or loose teeth, it's important to see your dentist right away. They can diagnose the problem and recommend appropriate treatment.

C. Yes, the body can reject a dental implant. However, this is a relatively rare occurrence. Dental implants are typically made of titanium, a biocompatible material that is less likely to be rejected by the body compared to other materials.

There are a few reasons why a body might reject a dental implant:

- Infection: Bacteria can infect the area around the implant, leading to inflammation and potential rejection.
- Poor oral hygiene: Not taking proper care of your oral health can increase the risk of infection and other complications.
- Jawbone issues: If your jawbone is not strong enough or healthy enough to support the implant, it may fail.

If you notice any signs of dental implant rejection, such as pain, swelling, or loose teeth, it's important to see your dentist right away. They can diagnose the problem and recommend appropriate treatment.

Q11: How long do dental implants typically last?

A.Dental implants are designed to be a permanent solution. With proper care, they can last for many years, often a lifetime.

Here are some factors that can influence their lifespan:

- Oral hygiene: Regular brushing, flossing, and professional cleanings are crucial for maintaining implant health.
- Jawbone density: A strong jawbone is essential for successful implant integration.
- Smoking: Smoking can hinder healing and increase the risk of complications.
- Bite force: Excessive bite force can put stress on implants.
- Implant quality and placement: The quality of the implant and the skill of the dentist performing the procedure are significant factors.

Typically, the lifespan of dental implants ranges from 10 to 30 years. However, with good care, many people can expect their implants to last much longer.

If you're considering dental implants, it's important to consult with a qualified dentist to discuss your specific needs and expectations.

B. Dental implants are designed to be a permanent solution. With proper care, they can last for many years, often a lifetime.

Here are some factors that can influence their lifespan:

- Oral hygiene: Regular brushing, flossing, and professional cleanings are crucial for maintaining implant health.
- Jawbone density: A strong jawbone is essential for successful implant integration.
- Smoking: Smoking can hinder healing and increase the risk of complications.
- Bite force: Excessive bite force can put stress on implants.
- Implant quality and placement: The quality of the implant and the skill of the dentist performing the procedure are significant factors.

Typically, the lifespan of dental implants ranges from 10 to 30 years. However, with good care, many people can expect their implants to last much longer.

If you're considering dental implants, it's important to consult with a qualified dentist to discuss your specific needs and expectations.

C. Dental implants are designed to be a permanent solution. With proper care, they can last for many years, often a lifetime.

Here are some factors that can influence their lifespan:

- Oral hygiene: Regular brushing, flossing, and professional cleanings are crucial for maintaining implant health.
- Jawbone density: A strong jawbone is essential for successful implant integration.
- Smoking: Smoking can hinder healing and increase the risk of complications.
- Bite force: Excessive bite force can put stress on implants.
- Implant quality and placement: The quality of the implant and the skill of the dentist performing the procedure are significant factors.

Typically, the lifespan of dental implants ranges from 10 to 30 years. However, with good care, many people can expect their implants to last much longer.

If you're considering dental implants, it's important to consult with a qualified dentist to discuss your specific needs and expectations.

(*Continued*)

 © 2025. The Author(s).

Supplementary Table S1 (*Continued*)

Q12: **What are the potential risks associated with dental implants?**
A.While dental implants are generally considered a safe and effective treatment, there are potential risks involved.These risks can vary depending on individual factors and the quality of the procedure.
Here are some potential complications associated with dental implants:
- Infection: Infections can occur around the implant site, especially if proper oral hygiene is not maintained.
- Peri-implantitis: This is a type of gum disease that can develop around implants. It can lead to bone loss and implant failure if not treated promptly.
- Nerve damage: In some cases, implants can damage nearby nerves, causing pain or numbness.
- Sinus perforation: If implants are placed in the upper jaw, there is a risk of perforating the maxillary sinus.
- Implant failure: Implants may fail if they do not integrate properly with the jawbone.
- Aesthetic concerns: In rare cases, implants may not look or feel natural.

B. While dental implants are generally considered a safe and effective treatment, there are potential risks involved.These risks can vary depending on individual factors and the quality of the procedure.
Here are some potential complications associated with dental implants:
- Infection: Infections can occur around the implant site, especially if proper oral hygiene is not maintained.
- Peri-implantitis: This is a type of gum disease that can develop around implants. It can lead to bone loss and implant failure if not treated promptly.
- Nerve damage: In some cases, implants can damage nearby nerves, causing pain or numbness.
- Sinus perforation: If implants are placed in the upper jaw, there is a risk of perforating the maxillary sinus.
- Implant failure: Implants may fail if they do not integrate properly with the jawbone.
- Aesthetic concerns: In rare cases, implants may not look or feel natural.

It’s important to discuss these potential risks with your dentist before deciding to undergo dental implant treatment. They can assess your individual risk factors and help you make an informed decision.
C. While dental implants are generally considered a safe and effective treatment, there are potential risks involved.These risks can vary depending on individual factors and the quality of the procedure.
Here are some potential complications associated with dental implants:
- Infection: Infections can occur around the implant site, especially if proper oral hygiene is not maintained.
- Peri-implantitis: This is a type of gum disease that can develop around implants. It can lead to bone loss and implant failure if not treated promptly.
- Nerve damage: In some cases, implants can damage nearby nerves, causing pain or numbness.
- Sinus perforation: If implants are placed in the upper jaw, there is a risk of perforating the maxillary sinus.
- Implant failure: Implants may fail if they do not integrate properly with the jawbone.
- Aesthetic concerns: In rare cases, implants may not look or feel natural.

It’s important to discuss these potential risks with your dentist before deciding to undergo dental implant treatment. They can assess your individual risk factors and help you make an informed decision.
It’s important to discuss these potential risks with your dentist before deciding to undergo dental implant treatment. They can assess your individual risk factors and help you make an informed decision.
Q13: Can I get Dental implant during pregnancy?
A. Generally, it’s recommended to avoid dental implant surgery during pregnancy.
Here’s why:
- Risk of complications: Pregnancy can weaken the immune system and increase the risk of infection and other complications after surgery.
- Medication limitations: Some medications used during dental implant procedures may not be safe for pregnant women.
- Discomfort: Pregnancy can make dental procedures more uncomfortable.
- Potential impact on the baby: While there is limited research, some concerns exist about the potential long-term effects of dental implants on the developing fetus.

If you have a dental emergency during pregnancy, consult with your dentist and obstetrician. They can discuss the risks and benefits and determine the best course of action for you and your baby.
It’s usually best to postpone elective dental procedures like dental implants until after pregnancy. This will allow for a safer and more successful treatment.
B. Generally, it’s recommended to avoid dental implant surgery during pregnancy.
Here’s why:
- Risk of complications: Pregnancy can weaken the immune system and increase the risk of infection and other complications after surgery.
- Medication limitations: Some medications used during dental implant procedures may not be safe for pregnant women.
- Discomfort: Pregnancy can make dental procedures more uncomfortable.
- Potential impact on the baby: While there is limited research, some concerns exist about the potential long-term effects of dental implants on the developing fetus.

If you have a dental emergency during pregnancy, consult with your dentist and obstetrician. They can discuss the risks and benefits and determine the best course of action for you and your baby.
It’s usually best to postpone elective dental procedures like dental implants until after pregnancy. This will allow for a safer and more successful treatment.
C. Generally, it’s recommended to avoid dental implant surgery during pregnancy.
Here’s why:
- Risk of complications: Pregnancy can weaken the immune system and increase the risk of infection and other complications after surgery.

Supplementary Table S1 (*Continued*)

• Medication limitations: Some medications used during dental implant procedures may not be safe for pregnant women.<br>• Discomfort: Pregnancy can make dental procedures more uncomfortable.<br>• Potential impact on the baby: While there is limited research, some concerns exist about the potential long-term effects of dental implants on the developing fetus.<br>If you have a dental emergency during pregnancy, consult with your dentist and obstetrician. They can discuss the risks and benefits and determine the best course of action for you and your baby.<br>It's usually best to postpone elective dental procedures like dental implants until after pregnancy. This will allow for a safer and more successful treatment.<br>**Q14: What is peri-implantitis?**<br>A. Peri-implantitis is a destructive inflammatory process that affects the soft and hard tissues surrounding dental implants.It's similar to gum disease in natural teeth.<br>Here's a breakdown:<br>• Soft tissue inflammation: The gums around the implant become red, swollen, and may bleed easily.<br>• Bone loss: Over time, the bone supporting the implant can deteriorate, leading to implant instability.<br>• Bacteria buildup: Bacteria can accumulate on the implant surface and beneath the gums, contributing to inflammation and bone loss.<br>Causes:<br>• Poor oral hygiene: Not brushing and flossing regularly allows bacteria to build up.<br>• Smoking: Smoking can impair blood flow to the gums and weaken the immune system.<br>• Diabetes: Uncontrolled diabetes can increase the risk of infections, including peri-implantitis.<br>• Other risk factors: Factors like bruxism (teeth grinding) and certain medications can also contribute.<br>Symptoms:<br>• Red, swollen, or tender gums around the implant<br>• Bleeding when brushing or flossing<br>• Bad taste or odor<br>• Loose implant<br>• Pain or sensitivity around the implant<br>Treatment:<br>• Professional cleaning: A dentist or periodontist can clean the implant and surrounding tissues to remove bacteria and plaque.<br>• Antibiotics: Antibiotics may be prescribed to treat infections.<br>• Surgical treatment: In severe cases, surgery may be necessary to clean the implant or regenerate bone.<br>Prevention:<br>• Good oral hygiene: Brush and floss regularly, and use a mouthwash as recommended by your dentist.<br>• Regular dental checkups: See your dentist for regular checkups to monitor the health of your implants.<br>• Control risk factors: Manage underlying conditions like diabetes and avoid smoking.<br>If you have any concerns about your dental implants or suspect you may have peri-implantitis, it's important to consult with a dentist or periodontist for proper diagnosis and treatment.<br>B. Peri-implantitis is a destructive inflammatory process that affects the soft and hard tissues surrounding dental implants.It's similar to gum disease in natural teeth.<br>Here's a breakdown:<br>• Soft tissue inflammation: The gums around the implant become red, swollen, and may bleed easily.<br>• Bone loss: Over time, the bone supporting the implant can deteriorate, leading to implant instability.<br>• Bacteria buildup: Bacteria can accumulate on the implant surface and beneath the gums, contributing to inflammation and bone loss.<br>Causes:<br>• Poor oral hygiene: Not brushing and flossing regularly allows bacteria to build up.<br>• Smoking: Smoking can impair blood flow to the gums and weaken the immune system.<br>• Diabetes: Uncontrolled diabetes can increase the risk of infections, including peri-implantitis.<br>• Other risk factors: Factors like bruxism (teeth grinding) and certain medications can also contribute.<br>Symptoms:<br>• Red, swollen, or tender gums around the implant<br>• Bleeding when brushing or flossing<br>• Bad taste or odor<br>• Loose implant<br>• Pain or sensitivity around the implant<br>Treatment:<br>• Professional cleaning: A dentist or periodontist can clean the implant and surrounding tissues to remove bacteria and plaque.<br>• Antibiotics: Antibiotics may be prescribed to treat infections.<br>• Surgical treatment: In severe cases, surgery may be necessary to clean the implant or regenerate bone.<br>Prevention:<br>• Good oral hygiene: Brush and floss regularly, and use a mouthwash as recommended by your dentist.<br>• Regular dental checkups: See your dentist for regular checkups to monitor the health of your implants.<br>• Control risk factors: Manage underlying conditions like diabetes and avoid smoking.

(*Continued*)

 © 2025. The Author(s).

Supplementary Table S1 (*Continued*)

If you have any concerns about your dental implants or suspect you may have peri-implantitis, it's important to consult with a dentist or periodontist for proper diagnosis and treatment.
C. Peri-implantitis is a destructive inflammatory process that affects the soft and hard tissues surrounding dental implants.It's similar to gum disease in natural teeth.
Here's a breakdown:
- Soft tissue inflammation: The gums around the implant become red, swollen, and may bleed easily.
- Bone loss: Over time, the bone supporting the implant can deteriorate, leading to implant instability.
- Bacteria buildup: Bacteria can accumulate on the implant surface and beneath the gums, contributing to inflammation and bone loss.

Causes:
- Poor oral hygiene: Not brushing and flossing regularly allows bacteria to build up.
- Smoking: Smoking can impair blood flow to the gums and weaken the immune system.
- Diabetes: Uncontrolled diabetes can increase the risk of infections, including peri-implantitis.
- Other risk factors: Factors like bruxism (teeth grinding) and certain medications can also contribute.

Symptoms:
- Red, swollen, or tender gums around the implant
- Bleeding when brushing or flossing
- Bad taste or odor
- Loose implant
- Pain or sensitivity around the implant

Treatment:
- Professional cleaning: A dentist or periodontist can clean the implant and surrounding tissues to remove bacteria and plaque.
- Antibiotics: Antibiotics may be prescribed to treat infections.
- Surgical treatment: In severe cases, surgery may be necessary to clean the implant or regenerate bone.

Prevention:
- Good oral hygiene: Brush and floss regularly, and use a mouthwash as recommended by your dentist.
- Regular dental checkups: See your dentist for regular checkups to monitor the health of your implants.
- Control risk factors: Manage underlying conditions like diabetes and avoid smoking.

If you have any concerns about your dental implants or suspect you may have peri-implantitis, it's important to consult with a dentist or periodontist for proper diagnosis and treatment.
Q15: . How many dental implants do I need if I want to restore my edntolus upper and lower Jaw?
A.The number of dental implants needed to restore your edentulous upper and lower jaws will depend on several factors, including:
- The condition of your jawbone: If your jawbone has deteriorated due to bone loss, you may need more implants to provide adequate support for your prosthesis.
- The type of prosthesis you want: A fixed bridge or denture will require a different number of implants than a removable denture.
- Your personal preferences: You may have a preference for a certain number of implants or a specific type of prosthesis.

Here are some common options:
- All-on-4: This technique uses four implants per jaw to support a full arch of teeth.
- All-on-6: This technique uses six implants per jaw to support a full arch of teeth.
- Traditional implants: This technique uses multiple implants to support a fixed bridge or denture.

It is important to consult with a qualified dentist to determine the best number of implants for your individual needs. They will be able to assess your jawbone, discuss your options, and recommend a treatment plan that is right for you.
Here are some additional resources that you may find helpful:
- Treatment of the edentulous patient: https://pubmed.ncbi.nlm.nih.gov/30825989/
- Implant rehabilitation in the edentulous jaw: https://www.ncbi.nlm.nih.gov/pmc/articles/PMC7396035/
- Number of implants maxilla and mandible: https://www.for.org/en/treat/treatment-guidelines/edentulous/treatment-options/implant-retainedsupported-general/number-implants-maxilla-and-mandible
  I hope this information is helpful!

B. The number of dental implants needed to restore your edentulous upper and lower jaws will depend on several factors, including:
- The condition of your jawbone: If your jawbone has deteriorated due to bone loss, you may need more implants to provide adequate support for your prosthesis.
- The type of prosthesis you want: A fixed bridge or denture will require a different number of implants than a removable denture.
- Your personal preferences: You may have a preference for a certain number of implants or a specific type of prosthesis.

Here are some common options:
- All-on-4: This technique uses four implants per jaw to support a full arch of teeth.
- All-on-6: This technique uses six implants per jaw to support a full arch of teeth.
- Traditional implants: This technique uses multiple implants to support a fixed bridge or denture.

It is important to consult with a qualified dentist to determine the best number of implants for your individual needs. They will be able to assess your jawbone, discuss your options, and recommend a treatment plan that is right for you.
Here are some additional resources that you may find helpful:

Supplementary Table S1 (*Continued*)

- Treatment of the edentulous patient: https://pubmed.ncbi.nlm.nih.gov/30825989/
- Implant rehabilitation in the edentulous jaw: https://www.ncbi.nlm.nih.gov/pmc/articles/PMC7396035/
- Number of implants maxilla and mandible: https://www.for.org/en/treat/treatment-guidelines/edentulous/treatment-options/implant-retainedsupported-general/number-implants-maxilla-and-mandible

I hope this information is helpful!
C. The number of dental implants needed to restore your edentulous upper and lower jaws will depend on several factors, including:

- The condition of your jawbone: If your jawbone has deteriorated due to bone loss, you may need more implants to provide adequate support for your prosthesis.
- The type of prosthesis you want: A fixed bridge or denture will require a different number of implants than a removable denture.
- Your personal preferences: You may have a preference for a certain number of implants or a specific type of prosthesis.

Here are some common options:

- All-on-4: This technique uses four implants per jaw to support a full arch of teeth.
- All-on-6: This technique uses six implants per jaw to support a full arch of teeth.
- Traditional implants: This technique uses multiple implants to support a fixed bridge or denture.

It is important to consult with a qualified dentist to determine the best number of implants for your individual needs. They will be able to assess your jawbone, discuss your options, and recommend a treatment plan that is right for you.
Here are some additional resources that you may find helpful:

- Treatment of the edentulous patient: https://pubmed.ncbi.nlm.nih.gov/30825989/
- Implant rehabilitation in the edentulous jaw: https://www.ncbi.nlm.nih.gov/pmc/articles/PMC7396035/
- Number of implants maxilla and mandible: https://www.for.org/en/treat/treatment-guidelines/edentulous/treatment-options/implant-retainedsupported-general/number-implants-maxilla-and-mandible

I hope this information is helpful!
Q16: Do I need to replace my dental implants in the future?
A. The lifespan of dental implants can vary, but they can last for many years with proper care. While some implants have lasted for decades, factors like oral hygiene, bite, and overall health can influence their longevity.
Here are some factors that might affect the lifespan of your dental implants:

- Oral hygiene: Maintaining good oral hygiene, including brushing, flossing, and regular dental check-ups, is crucial for the health of your implants and surrounding tissues.
- Bite: If you have a misaligned bite, it can put extra stress on your implants and increase the risk of wear and tear.
- Overall health: Chronic health conditions or certain medications can affect the health of your gums and bones, which can impact the longevity of your implants.

It's important to see your dentist regularly for check-ups to monitor the condition of your implants and address any potential issues early on. Your dentist can provide more specific information based on your individual situation and help you determine when to schedule follow-up appointments.
Would you like to know more about the importance of dental implant maintenance or the signs of potential problems?
B. The lifespan of dental implants can vary, but they can last for many years with proper care. While some implants have lasted for decades, factors like oral hygiene, bite, and overall health can influence their longevity.
Here are some factors that might affect the lifespan of your dental implants:

- Oral hygiene: Maintaining good oral hygiene, including brushing, flossing, and regular dental check-ups, is crucial for the health of your implants and surrounding tissues.
- Bite: If you have a misaligned bite, it can put extra stress on your implants and increase the risk of wear and tear.
- Overall health: Chronic health conditions or certain medications can affect the health of your gums and bones, which can impact the longevity of your implants.

It's important to see your dentist regularly for check-ups to monitor the condition of your implants and address any potential issues early on. Your dentist can provide more specific information based on your individual situation and help you determine when to schedule follow-up appointments.
Would you like to know more about the importance of dental implant maintenance or the signs of potential problems?
C. The lifespan of dental implants can vary, but they can last for many years with proper care. While some implants have lasted for decades, factors like oral hygiene, bite, and overall health can influence their longevity.
Here are some factors that might affect the lifespan of your dental implants:

- Oral hygiene: Maintaining good oral hygiene, including brushing, flossing, and regular dental check-ups, is crucial for the health of your implants and surrounding tissues.
- Bite: If you have a misaligned bite, it can put extra stress on your implants and increase the risk of wear and tear.
- Overall health: Chronic health conditions or certain medications can affect the health of your gums and bones, which can impact the longevity of your implants.

It's important to see your dentist regularly for check-ups to monitor the condition of your implants and address any potential issues early on. Your dentist can provide more specific information based on your individual situation and help you determine when to schedule follow-up appointments.
Would you like to know more about the importance of dental implant maintenance or the signs of potential problems?
Q17: Can I replace my complete denture with implant supported dental prothesis?
A. Yes, you can replace your complete denture with implant-supported dental prostheses.
Implant-supported dentures offer several advantages over traditional dentures, including:

- Improved stability: Implants anchor directly to your jawbone, providing a more secure fit and reducing slippage.

(*Continued*)

Supplementary Table S1 (*Continued*)

- Enhanced comfort: The stability provided by implants can make eating, speaking, and smiling more comfortable.
- Better chewing ability: Implants can improve your ability to chew a wider variety of foods.
- Improved aesthetics: Implant-supported dentures can look and feel more natural than traditional dentures.

However, it's important to note that implant-supported dentures require a surgical procedure. This involves placing dental implants into your jawbone and allowing them to heal. The healing process can take several months.
To determine if implant-supported dentures are right for you, it's best to consult with a qualified dentist or oral surgeon. They can assess your oral health and discuss the potential benefits and risks of this procedure.
Would you like to know more about the process of getting implant-supported dentures?
B. Yes, you can replace your complete denture with implant-supported dental prostheses.
Implant-supported dentures offer several advantages over traditional dentures, including:

- Improved stability: Implants anchor directly to your jawbone, providing a more secure fit and reducing slippage.
- Enhanced comfort: The stability provided by implants can make eating, speaking, and smiling more comfortable.
- Better chewing ability: Implants can improve your ability to chew a wider variety of foods.
- Improved aesthetics: Implant-supported dentures can look and feel more natural than traditional dentures.

However, it's important to note that implant-supported dentures require a surgical procedure. This involves placing dental implants into your jawbone and allowing them to heal. The healing process can take several months.
To determine if implant-supported dentures are right for you, it's best to consult with a qualified dentist or oral surgeon. They can assess your oral health and discuss the potential benefits and risks of this procedure.
Would you like to know more about the process of getting implant-supported dentures?
C. Yes, you can replace your complete denture with implant-supported dental prostheses.
Implant-supported dentures offer several advantages over traditional dentures, including:

- Improved stability: Implants anchor directly to your jawbone, providing a more secure fit and reducing slippage.
- Enhanced comfort: The stability provided by implants can make eating, speaking, and smiling more comfortable.
- Better chewing ability: Implants can improve your ability to chew a wider variety of foods.
- Improved aesthetics: Implant-supported dentures can look and feel more natural than traditional dentures.

However, it's important to note that implant-supported dentures require a surgical procedure. This involves placing dental implants into your jawbone and allowing them to heal. The healing process can take several months.
To determine if implant-supported dentures are right for you, it's best to consult with a qualified dentist or oral surgeon. They can assess your oral health and discuss the potential benefits and risks of this procedure.
Would you like to know more about the process of getting implant-supported dentures?
Q18: How should I care of my dental implant?
A. Caring for Your Dental Implant: A Guide
Dental implants are a significant investment in your oral health. Proper care can help them last for many years. Here's a breakdown of how to maintain your dental implant:
Daily Oral Hygiene

- Brush twice daily: Use a soft-bristled toothbrush and a fluoride toothpaste. Be sure to brush around and under the implant crown.
- Floss daily: Use floss or interdental brushes to clean between your teeth and around the implant.
- Use a mouthwash: A mouthwash can help kill bacteria that can cause plaque and gum disease.

Regular Dental Check-ups

- Schedule regular check-ups: Visit your dentist for routine check-ups to monitor the health of your implant.
- Professional cleanings: These cleanings can remove plaque and tartar buildup that can lead to gum disease.

Additional Tips

- Avoid smoking: Smoking can impair healing and increase the risk of complications.
- Limit alcohol consumption: Excessive alcohol use can irritate the gums.
- Eat a balanced diet: A healthy diet provides the nutrients your body needs to maintain oral health.
- Avoid hard foods: Hard foods can damage the implant crown.
- Wear a mouthguard: If you grind your teeth at night, a mouthguard can protect your implant.

By following these guidelines, you can help ensure the long-term success of your dental implant. If you have any questions or concerns, don't hesitate to consult with your dentist.
Would you like to know more about dental implant maintenance or have other questions?
B. Caring for Your Dental Implant: A Guide
Dental implants are a significant investment in your oral health. Proper care can help them last for many years. Here's a breakdown of how to maintain your dental implant:
Daily Oral Hygiene

- Brush twice daily: Use a soft-bristled toothbrush and a fluoride toothpaste. Be sure to brush around and under the implant crown.
- Floss daily: Use floss or interdental brushes to clean between your teeth and around the implant.
- Use a mouthwash: A mouthwash can help kill bacteria that can cause plaque and gum disease.

Regular Dental Check-ups

- Schedule regular check-ups: Visit your dentist for routine check-ups to monitor the health of your implant.
- Professional cleanings: These cleanings can remove plaque and tartar buildup that can lead to gum disease.

Additional Tips

- Avoid smoking: Smoking can impair healing and increase the risk of complications.
- Limit alcohol consumption: Excessive alcohol use can irritate the gums.

 © 2025. The Author(s).

Supplementary Table S1 (*Continued*)

- Eat a balanced diet: A healthy diet provides the nutrients your body needs to maintain oral health.
- Avoid hard foods: Hard foods can damage the implant crown.
- Wear a mouthguard: If you grind your teeth at night, a mouthguard can protect your implant.

By following these guidelines, you can help ensure the long-term success of your dental implant. If you have any questions or concerns, don't hesitate to consult with your dentist.
Would you like to know more about dental implant maintenance or have other questions?
C. Caring for Your Dental Implant: A Guide
Dental implants are a significant investment in your oral health. Proper care can help them last for many years. Here's a breakdown of how to maintain your dental implant:
Daily Oral Hygiene

- Brush twice daily: Use a soft-bristled toothbrush and a fluoride toothpaste. Be sure to brush around and under the implant crown.
- Floss daily: Use floss or interdental brushes to clean between your teeth and around the implant.
- Use a mouthwash: A mouthwash can help kill bacteria that can cause plaque and gum disease.

Regular Dental Check-ups

- Schedule regular check-ups: Visit your dentist for routine check-ups to monitor the health of your implant.
- Professional cleanings: These cleanings can remove plaque and tartar buildup that can lead to gum disease.

Additional Tips

- Avoid smoking: Smoking can impair healing and increase the risk of complications.
- Limit alcohol consumption: Excessive alcohol use can irritate the gums.
- Eat a balanced diet: A healthy diet provides the nutrients your body needs to maintain oral health.
- Avoid hard foods: Hard foods can damage the implant crown.
- Wear a mouthguard: If you grind your teeth at night, a mouthguard can protect your implant.

By following these guidelines, you can help ensure the long-term success of your dental implant. If you have any questions or concerns, don't hesitate to consult with your dentist.
Would you like to know more about dental implant maintenance or have other questions?
Q19|:How long does the entire dental implant process take?
A. The entire dental implant process typically takes 6 to 9 months. However, this can vary depending on several factors, including:

- Bone quality: If your jawbone is weak or has insufficient density, you may need a bone graft before the implant can be placed.
- Number of implants: The more implants you need, the longer the process will take.
- Type of implant: Some implants may require additional steps or healing time.
- Individual healing time: Everyone heals at a different rate.

It's important to consult with your dentist to get a more accurate estimate for your specific situation. They can assess your needs and provide you with a personalized timeline.
B. The entire dental implant process typically takes 6 to 9 months. However, this can vary depending on several factors, including:

- Bone quality: If your jawbone is weak or has insufficient density, you may need a bone graft before the implant can be placed.
- Number of implants: The more implants you need, the longer the process will take.
- Type of implant: Some implants may require additional steps or healing time.
- Individual healing time: Everyone heals at a different rate.

It's important to consult with your dentist to get a more accurate estimate for your specific situation. They can assess your needs and provide you with a personalized timeline.
C. The entire dental implant process typically takes 6 to 9 months. However, this can vary depending on several factors, including:

- Bone quality: If your jawbone is weak or has insufficient density, you may need a bone graft before the implant can be placed.
- Number of implants: The more implants you need, the longer the process will take.
- Type of implant: Some implants may require additional steps or healing time.
- Individual healing time: Everyone heals at a different rate.

It's important to consult with your dentist to get a more accurate estimate for your specific situation. They can assess your needs and provide you with a personalized timeline.
Q20: 20. Is It Possible to Get "Metal-Free" Implants
A. Yes, it is possible to get metal-free implants.
These implants are typically made from zirconia, a ceramic material that is highly biocompatible and offers several advantages over traditional metal implants, including:

- No metal allergies: Zirconia is non-metallic, making it a good option for those with metal sensitivities or allergies.
- Natural appearance: Zirconia is white in color, closely resembling the appearance of natural teeth, especially when viewed through the gums.
- Durability: Zirconia is a strong and durable material, capable of withstanding the forces of chewing.
- Biocompatibility: Zirconia is well-tolerated by the body and promotes bone growth around the implant.

If you're considering metal-free implants, it's important to consult with a qualified dentist who can assess your individual needs and determine if they are the right option for you.
B. Yes, it is possible to get metal-free implants.
These implants are typically made from zirconia, a ceramic material that is highly biocompatible and offers several advantages over traditional metal implants, including:

(*Continued*)

 © 2025. The Author(s).

Supplementary Table S1 (*Continued*)

- No metal allergies: Zirconia is non-metallic, making it a good option for those with metal sensitivities or allergies.
- Natural appearance: Zirconia is white in color, closely resembling the appearance of natural teeth, especially when viewed through the gums.
- Durability: Zirconia is a strong and durable material, capable of withstanding the forces of chewing.
- Biocompatibility: Zirconia is well-tolerated by the body and promotes bone growth around the implant.

If you're considering metal-free implants, it's important to consult with a qualified dentist who can assess your individual needs and determine if they are the right option for you.
C. Yes, it is possible to get metal-free implants.
These implants are typically made from zirconia, a ceramic material that is highly biocompatible and offers several advantages over traditional metal implants, including:

- No metal allergies: Zirconia is non-metallic, making it a good option for those with metal sensitivities or allergies.
- Natural appearance: Zirconia is white in color, closely resembling the appearance of natural teeth, especially when viewed through the gums.
- Durability: Zirconia is a strong and durable material, capable of withstanding the forces of chewing.
- Biocompatibility: Zirconia is well-tolerated by the body and promotes bone growth around the implant.

If you're considering metal-free implants, it's important to consult with a qualified dentist who can assess your individual needs and determine if they are the right option for you.

Supplementary Table S2 Intraclass correlation between different chatbots in each round

Round	ICC	95% CI
1	0.393	0.117–0.663
2	0.358	0.083–0.637
3	0.344	0.069–0.626

 © 2025. The Author(s).
